# Trends in treatment-seeking for fever in children under five years old in 151 countries from 1990 to 2020

**DOI:** 10.1371/journal.pgph.0002134

**Published:** 2023-08-23

**Authors:** Michele Nguyen, Paulina A. Dzianach, Paul E. C. W. Castle, Susan F. Rumisha, Jennifer A. Rozier, Joseph R. Harris, Harry S. Gibson, Katherine A. Twohig, Camilo A. Vargas-Ruiz, Donal Bisanzio, Ewan Cameron, Daniel J. Weiss, Samir Bhatt, Peter W. Gething, Katherine E. Battle

**Affiliations:** 1 Asian School of the Environment, Nanyang Technological University, Singapore, Singapore; 2 Child Health Analytics, Telethon Kids Institute, Nedlands, WA, Australia; 3 Ordnance Survey, Southampton, United Kingdom; 4 Big Data Institute, Li Ka Shing Centre for Health Information and Discovery, University of Oxford, Oxford, United Kingdom; 5 RTI International, Washington, District of Columbia, United States of America; 6 Division of Epidemiology and Public Health, School of Medicine, University of Nottingham, Nottingham, United Kingdom; 7 Faculty of Health Sciences, Curtin University, Bentley, WA, Australia; 8 Department of Infectious Disease Epidemiology, Imperial College, St Mary’s Hospital, London, United Kingdom; 9 Department of Public Health, Section of Epidemiology, University of Copenhagen, Copenhagen, Denmark; 10 Institute for Disease Modeling, Bill & Melinda Gates Foundation, Seattle, Washington, United States of America; African Population and Health Research Center, KENYA

## Abstract

Access to medical treatment for fever is essential to prevent morbidity and mortality in individuals and to prevent transmission of communicable febrile illness in communities. Quantification of the rates at which treatment is accessed is critical for health system planning and a prerequisite for disease burden estimates. In this study, national data on the proportion of children under five years old with fever who were taken for medical treatment were collected from all available countries in Africa, Latin America, and Asia (n = 91). We used generalised additive mixed models to estimate 30-year trends in the treatment-seeking rates across the majority of countries in these regions (n = 151). Our results show that the proportions of febrile children brought for medical treatment increased steadily over the last 30 years, with the greatest increases occurring in areas where rates had originally been lowest, which includes Latin America and Caribbean, North Africa and the Middle East (51 and 50% increase, respectively), and Sub-Saharan Africa (23% increase). Overall, the aggregated and population-weighted estimate of children with fever taken for treatment at any type of facility rose from 61% (59–64 95% CI) in 1990 to 71% (69–72 95% CI) in 2020. The overall population-weighted average for fraction of treatment in the public sector was largely unchanged during the study period: 49% (42–58 95% CI) sought care at public facilities in 1990 and 47% (44–52 95% CI) in 2020. Overall, the findings indicate that improvements in access to care have been made where they were most needed, but that despite rapid initial gains, progress can plateau without substantial investment. In 2020 there remained significant gaps in care utilisation that must be factored in when developing control strategies and deriving disease burden estimates.

## Introduction

Improved access to treatment for febrile diseases not only results in decreased morbidity and mortality of patients but is also essential to prevent onward transmission of infectious diseases [1–3]. Effective treatment depends on the proportion of infections that are treated by health professionals, which in turn depends on the likelihood an individual will seek treatment. The latter is driven by a range of factors, including education, income, proximity to care, pre-existing relationships with the treatment provider, the likelihood of receiving a diagnosis, and the availability of effective drugs [4,5].

Adequate access to medical care not only improves individual treatment outcomes and prevents onward transmission of communicable diseases, but it also serves as a means to monitor disease burden and risk. Measures of local incidence reported from points of care can be used to generate fine scale maps of risk across a country or region [4]. At the national level, assembled case reports from a routine surveillance system can inform global estimates of the burden of disease. Both local and national estimates, however, must be adjusted for treatment-seeking behaviours to account for cases that do not present at a facility within the surveillance system [6,7]. Another key factor that leads to under-estimates of the ‘true’ burden of disease is whether patient records make their way into the national routine surveillance system. Records may fail to enter national databases due to, for example, lapses in facility reporting or failure to adequately extend national surveillance efforts to the private sector healthcare providers [8]. The latter of which may be estimated as a ‘reporting completeness’ metric, while the former is captured in surveys that ask respondents about which type of care they sought for fever.

Existing work quantifying treatment-seeking rates for fever across multiple regions was either limited to malaria-endemic countries [9] or did not produce country-specific time series [10]. This study builds on the existing methodology [9] by widening the geographic scope to more countries, regardless of malaria endemicity, by greatly expanding the number of response datasets, and improving the uncertainty estimates associated with the results. The aim of this study was to estimate the proportion of children under five for whom treatment was sought for fever from 1990 to 2020 in 151 countries. The percentages for seeking treatment at any location where legitimate medication may be obtained (public or private), and the fraction of treatment sought in the public sector were determined for each country-year in the time series. The distinction between public and overall treatment-seeking rates may aid in creating adjustments of patient records from national routine surveillance systems. A suite of modelled outputs was developed, including maps and time series. These outputs facilitated analyses of changing treatment-seeking propensity and preference through time. Observed patterns illustrate where greatest gains have been made and set benchmarks for improvements still to come.

## Materials and methods

### Data assembly

#### Data sources

**Fig 1:**
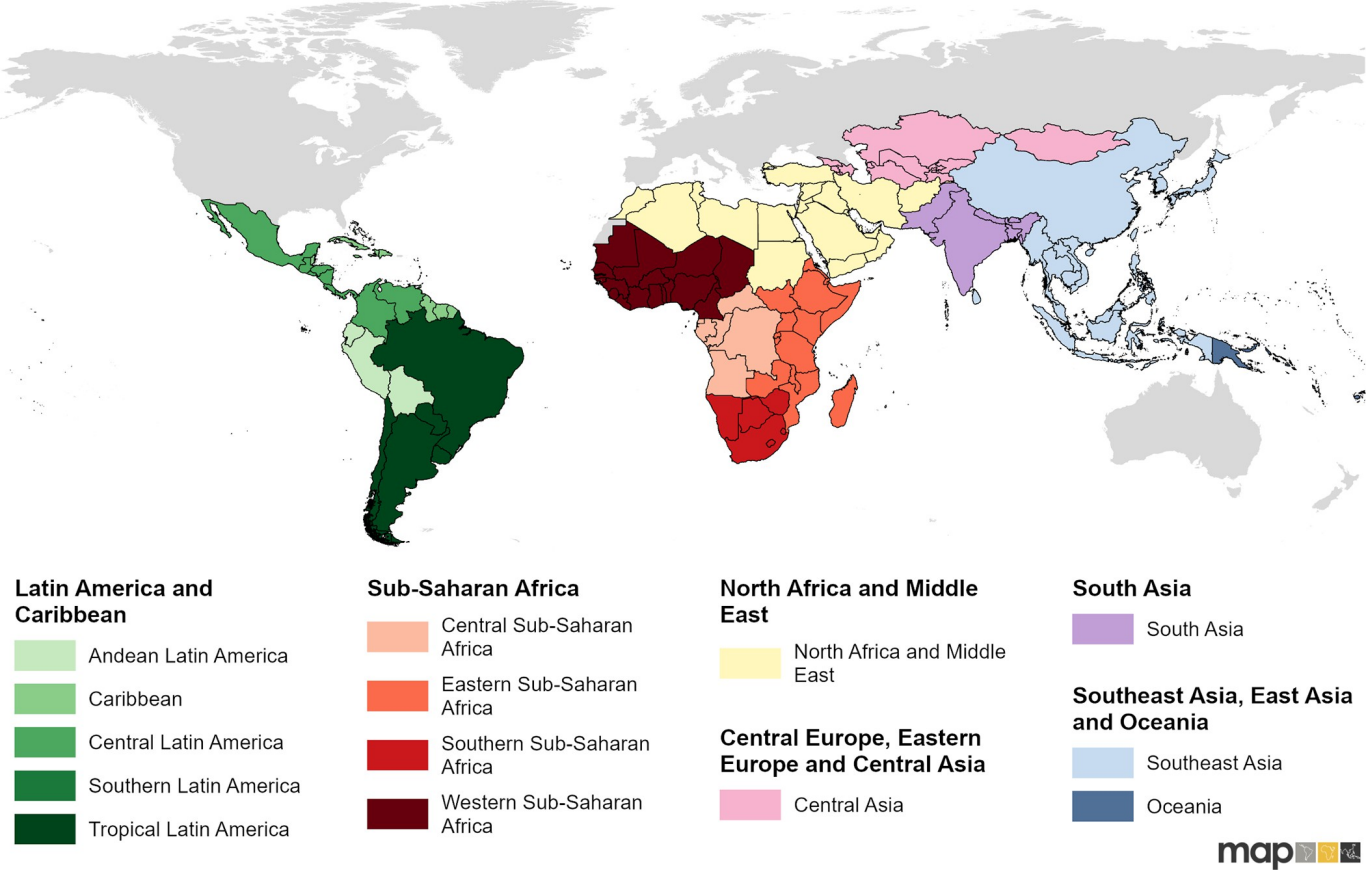
Geographical scope of the study. The scope of this study with geographical classifications for the modelling analysis- the classifications were taken from IHME Global Burden of Disease study [13], with slight modifications. Global national shapefile obtained from the Malaria Atlas Project (MAP; https://malariaatlas.org/) and available for download through the malariaAtlas R package.

Data on **treatment-seeking behaviours** (meaning the decision to seek care and where) were gathered from Demographic Health Surveys (DHS), AIDS Indicator Surveys (AIS), Malaria Indicator Surveys (MIS), and Multiple Indicator Cluster Surveys (MICS) conducted between 1990 and 2021 in Africa, Latin America, and Asia (Russia, Australasia, and Western Sahara were excluded from this analysis) [11,12]. The geographical scope of the study is depicted in Fig 1. The individual responses for the following questions were extracted from these surveys, which relate to children under five years of age: “Has (NAME) been ill with fever at any time in the last 2 weeks?”, “Did you seek advice or treatment for the illness from any source?”, and “Where did you seek advice or treatment?”. **Fever** was defined as any instance where the caretaker of the child answered “YES” to the first question. Response codes from the third question were manually classified into public points of care, such as government hospitals, clinics, or community health workers, and any medical treatment, which included all public treatment as well as any other locations where it is expected that people can obtain legitimate medication: private and non-governmental organisation (NGO) health facilities, privately owned pharmacies, and other retail facilities. Friends, family, and traditional or homeopathic healers were not considered as medical treatment.

#### Data processing

Individual responses grouped by year were aggregated to a national level, using the sampling weights provided in the survey datasets [11,12]. **Any medical treatment rates** were determined from the proportion of children under five years old reported to have fever in the past two weeks by their caretakers, for whom medical treatment at any sector was sought. **Public treatment fractions** were calculated as the proportion of children under five years old who were taken to seek care in the public sector, relative to all children taken for medical treatment. All national surveys which included the questions listed above and were gathered in the study region were included in this analysis. A total of 304 surveys were extracted, which covered 91 out of 151 modelled countries. Out of all included surveys, 179 were from Sub-Saharan Africa, 42 from Latin America and Caribbean, 28 from Southeast Asia, East Asia, and Oceania, 20 from South Asia, 20 from North Africa and Middle East, and 15 from Central Asia (see S3 Table for the list of the surveys). Overall, treatment-seeking data from 654,460 children under five years old who experienced fever symptoms was gathered for this model.

### Ethics

The analysis was based on publicly available datasets from the DHS Program and MICS websites [11,12]. The permission to access the data was obtained through the DHS and MICS websites and therefore no further ethical approval was necessary. The DHS program specifies that procedures and questionnaires were reviewed and approved by ICF Institutional Review Board (IRB). Country-specific DHS survey protocols were “reviewed by the ICF IRB and typically by an IRB in the host country. ICF IRB ensures that the survey complies with the U.S. Department of Health and Human Services regulations for the protection of human subjects (45 CFR 46), while the host country IRB ensures that the survey complies with laws and norms of the nation” [14].

### Model approach

#### Modelling framework

Statistical modelling was implemented in the R programming language [15] with the mgcv package [16]. Generalised additive mixed models (GAMMs) [17] were applied to smooth and estimate treatment-seeking rates for the 151 countries for the years 1990 to 2020. These models allowed for regional intercepts and non-linear trends, non-linear effects of the socio-economic covariates, and country random effects.

#### Covariate selection

We used covariates developed by the Institute for Health Metrics and Evaluation (IHME) for the Global Burden of Disease Study [13] (see S1 Appendix for more details). The covariates used in the model were identified as strong indicators of treatment-seeking rates within a previous literature review and statistical analysis [9]. Box-Cox plots were used to determine whether covariates required normalisation, and square or logarithmic transformations were performed accordingly. The covariates were then standardised to the same scale before modelling to ensure comparable coefficient sizes. From the set of considered covariates, a subset was selected based on the Akaike Information Criterion (AIC) and p-values (Table 1), using the ‘MuMIn’ R package [18].

**Table 1:** Covariates selected for the treatment-seeking models.

Stratification	Covariate	Transformation	Source
Any medical treatment	Proportion of pregnant women receiving any antenatal care from a skilled provider	square	IHME [13]
	Health expenditure per capita (USD)	log	IHME [13]
Public fraction	Percent of women giving birth in a health facility	-	IHME [13]
	Hospital beds per 1000 people	log	IHME [13]
	Urbanicity	-	IHME [13]

#### Model development

Two separate GAMMs were constructed–one for any medical treatment and one for the fraction of people seeking treatment at a public health facility (public fraction). The public treatment-seeking rates (S1 Table) were later computed by multiplying the two estimates. Each GAMM took the following form:

$$logit(Y)=\beta_{country}+\beta_{region}+s_{region}(year)+\sum s_{k}(x_{k,})+\epsilon ,$$ (1)

where *Y* denotes the estimated any medical treatment-seeking rate or the estimated public fraction. *β*_*region*_ is a region-specific intercept, *β*_*country*_ is the country random effect, *x*_*k*_ is the mean of the *k*^th^ covariate, and *ϵ* denotes the Gaussian random error. Splines, which are denoted by *s*, allowed for non-linear region-specific temporal trends and effects of the covariates. To prevent overfitting and to facilitate interpretation, the maximum basis dimensions of the splines were set to five for the region-specific trends and three for the non-linear covariate effects. The logit transformation was chosen to map the rates and fractions from (0, 1) to the real numbers for modelling purposes.

To match countries without data to a similar country with data in the same region, a nearest-neighbour algorithm was performed on the principal components of the identified covariates in the years 2000, 2010 and 2020. The random effect of the matched unit was used for prediction. Similarly, the differences in the covariates between 2000, 2010 and 2020 were used to determine the regional temporal trend for units for which the available data spanned a period of less than 5 years.

In order to avoid unrealistic predictions at the tails of the study period, the temporal trend *s*_*region*_*(year)* was fixed for the years before the earliest data point in the dataset that was available for the given region, and for years 2019–2021, an average *s*_*region*_*(2019–2021)* temporal trend was estimated.

#### Model uncertainty

To account for the uncertainty in the treatment-seeking survey data as well as the covariates, the models were run one hundred times. Each run sampled from the range of the 95% confidence intervals (CIs) of the observed treatment-seeking rates and covariates. By drawing one hundred realisations from each model run, we obtained 10,000 realisations of the any medical treatment-seeking rate and public fraction for each country-year pair, from which we computed the 95% CIs. Further details on the modelling process can be found in S1 Appendix.

## Results

### Trends in treatment-seeking in 1990–2020

The proportion of children under five years old with fever within the past two weeks who accessed medical treatment increased in all study regions between 1990 and 2020 (Table 2). Overall, the aggregated and population-weighted estimate for the 151 countries included in this analysis rose from 61% (59–64 95% CI) in 1990 to 71% (69–72%) in 2020. The largest increases in overall treatment-seeking from 1990–2020 were estimated for Latin America and Caribbean (47% to 71%) and for North Africa and Middle East (44% to 66%).

**Table 2:** Population-weighted treatment-seeking aggregated by study regions, in any medical points of care and public points of care, with 95% confidence intervals.

Region	Year	Any medical treatment % (95% CI)	Public fraction % (95% CI)
Central Asia	1990	45(41–50)	97(91–99)
	2005	47(43–50)	95(89–99)
	2020	55(52–59)	95(88–99)
Latin America and Caribbean	1990	47(41–54)	54(45–63)
	2005	66(62–70)	67(61–73)
	2020	71(68–74)	77(71–81)
North Africa and Middle East	1990	44(41–47)	61(55–68)
	2005	61(58–63)	52(47–56)
	2020	66(63–68)	44(38–50)
South Asia	1990	67(60–74)	27(16–45)
	2005	70(63–76)	20(12–31)
	2020	78(71–82)	24(14–38)
Southeast Asia, East Asia, and Oceania	1990	73(68–78)	51(36–69)
	2005	77(74–80)	46(35–56)
	2020	80(76–83)	44(37–53)
Sub-Saharan Africa	1990	48(45–50)	66(59–74)
	2005	58(56–60)	58(53–63)
	2020	59(57–62)	62(57–66)
Combined	1990	61(59–64)	49(42–58)
	2005	68(65–70)	44(40–49)
	2020	71(69–72)	47(44–52)

**Fig 2:**
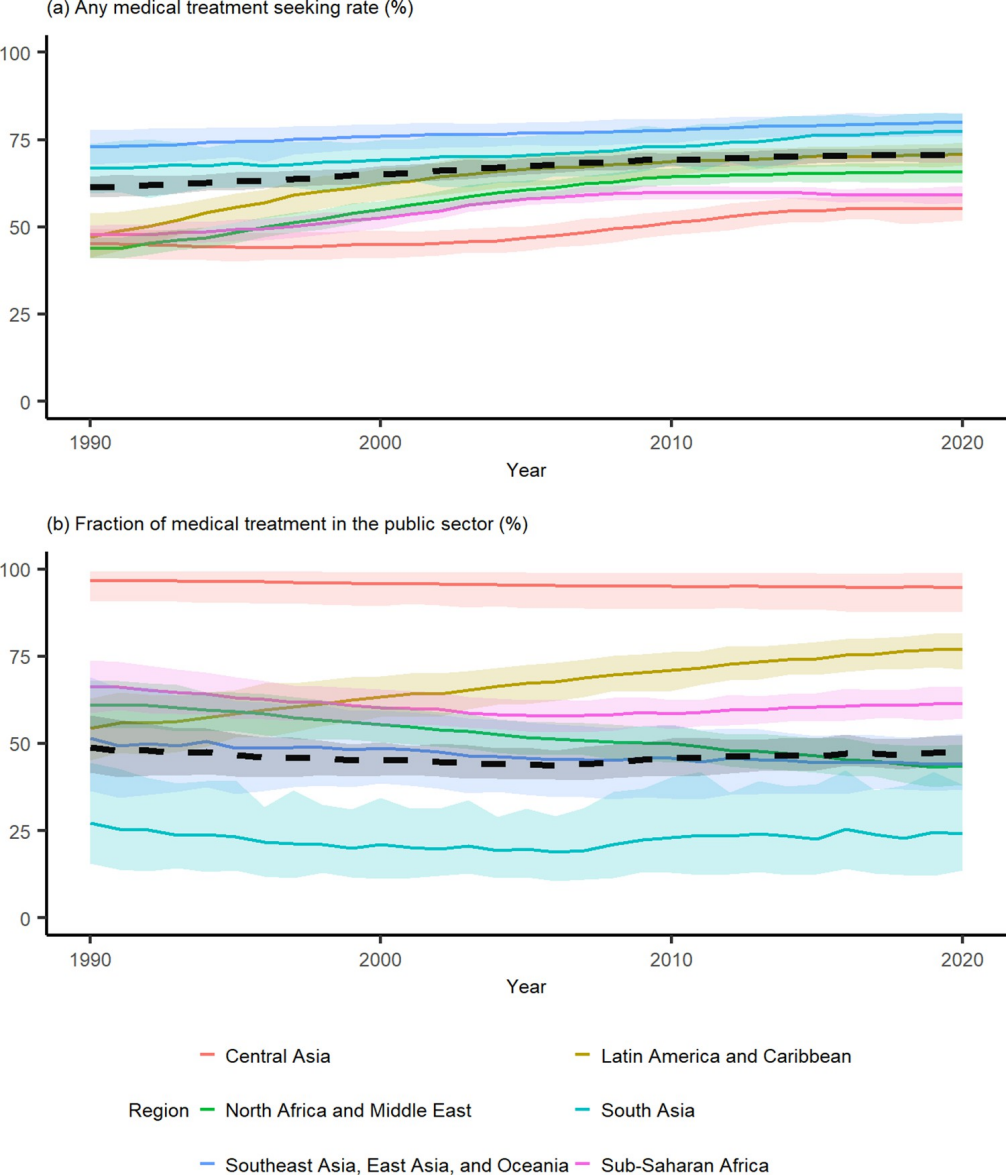
Regional treatment-seeking rates for 1990–2020. Population averaged estimates for (a) Any medical treatment-seeking rate and (b) Fraction of medical treatment in the public sector. The coloured envelopes denote the 95% uncertainty intervals. The black dashed line represents combined estimates for all 151 countries included in this study.

Fig 2(A) illustrates the temporal trend in regional treatment-seeking estimates in the study period for any medical treatment. Notably, we see that South Asia and Southeast Asia, East Asia, and Oceania regions were estimated to have the highest overall treatment-seeking rates throughout the study period. The remaining regions, in contrast, were estimated to experience steeper improvements in their treatment-seeking rates in the last 30 years. Among these study regions, the majority of the improvements were achieved between the 1990–2010 period. This finding aligns with the South and Southeast Asia, East Asia, and Oceania regional examples that indicate gains are harder to achieve with higher established rates of treatment-seeking. Numerically, this makes sense because the pool of individuals who are unwilling or unable to seek treatment shrinks as the national treatment-seeking rates rise. It should be noted that there was significant heterogeneity in treatment-seeking rates within the Southeast Asia, East Asia, and Oceania region. In particular, in Oceania, mean treatment-seeking rates below 50% were estimated for Papua New Guinea and Solomon Islands in 2020, which is significantly lower than the regional average and lower than in all other countries within this area. On the other hand, the countries classified as high income within the region (Japan, Brunei, South Korea, and Singapore) [19,20] had a higher estimated medical treatment-seeking rate than the regional average, with mean national treatment-seeking rates ranging between 83% and 85% in 2020. Other countries with similarly high treatment-seeking rates in the region were Taiwan, Cambodia, Maldives, and Indonesia.

The population-weighted treatment-seeking rate across all of Sub-Saharan Africa increased from 48% (45–50%) to 59% (57–62%) from the start to the end of the study period. The average estimated treatment-seeking rates increased in the study period for Eastern and Western Sub-Saharan Africa (Table 3). In Western Sub-Saharan Africa, the rate for any medical treatment-seeking increased from 42% (36–48%) to 62% (57–67%) in the 30-year study period, and in Eastern Sub-Saharan Africa, this rate increased from 47% (44–50%) to 60% (57–62%). The data which contributed the most to the estimated increase in treatment-seeking in Western Sub-Saharan Africa came from Sierra Leone, Senegal, Nigeria, Niger, Mali, Côte d’Ivoire, Cameroon, and Burkina Faso. In Eastern Sub-Saharan Africa, the upwards trend was mostly driven by Zambia, Tanzania, Rwanda, Mozambique, and Ethiopia (see country-level plots in S1 Fig). In contrast, in Central Sub-Saharan Africa and Southern Sub-Saharan Africa, the model predicted a slight but not statistically significant decline in the mean rate. In Southern Sub-Saharan Africa, while data from Lesotho suggests a steady upward trend, the observed trend in Namibia was flat, while the data from Zimbabwe suggested a drop in treatment-seeking rates within the study period (S1 Fig).

**Table 3:** Population-weighted treatment-seeking aggregated by individual regions in Sub-Saharan Africa, in any medical points of care and public points of care, with 95% confidence intervals.

Region	Year	Any medical treatment % (95% CI)	Public fraction % (95% CI)
Central Sub-Saharan Africa	1990	59(49–68)	60(43–78)
	2005	60(50–70)	61(44–76)
	2020	50(42–58)	59(45–74)
Eastern Sub-Saharan Africa	1990	47(44–50)	70(60–77)
	2005	53(50–55)	63(58–69)
	2020	60(57–62)	71(65–77)
Southern Sub-Saharan Africa	1990	63(58–68)	73(55–86)
	2005	61(56–65)	67(49–80)
	2020	59(55–64)	67(53–79)
Western Sub-Saharan Africa	1990	42(36–48)	64(49–77)
	2005	62(59–65)	51(42–58)
	2020	62(57–67)	54(47–62)

**Fig 3:**
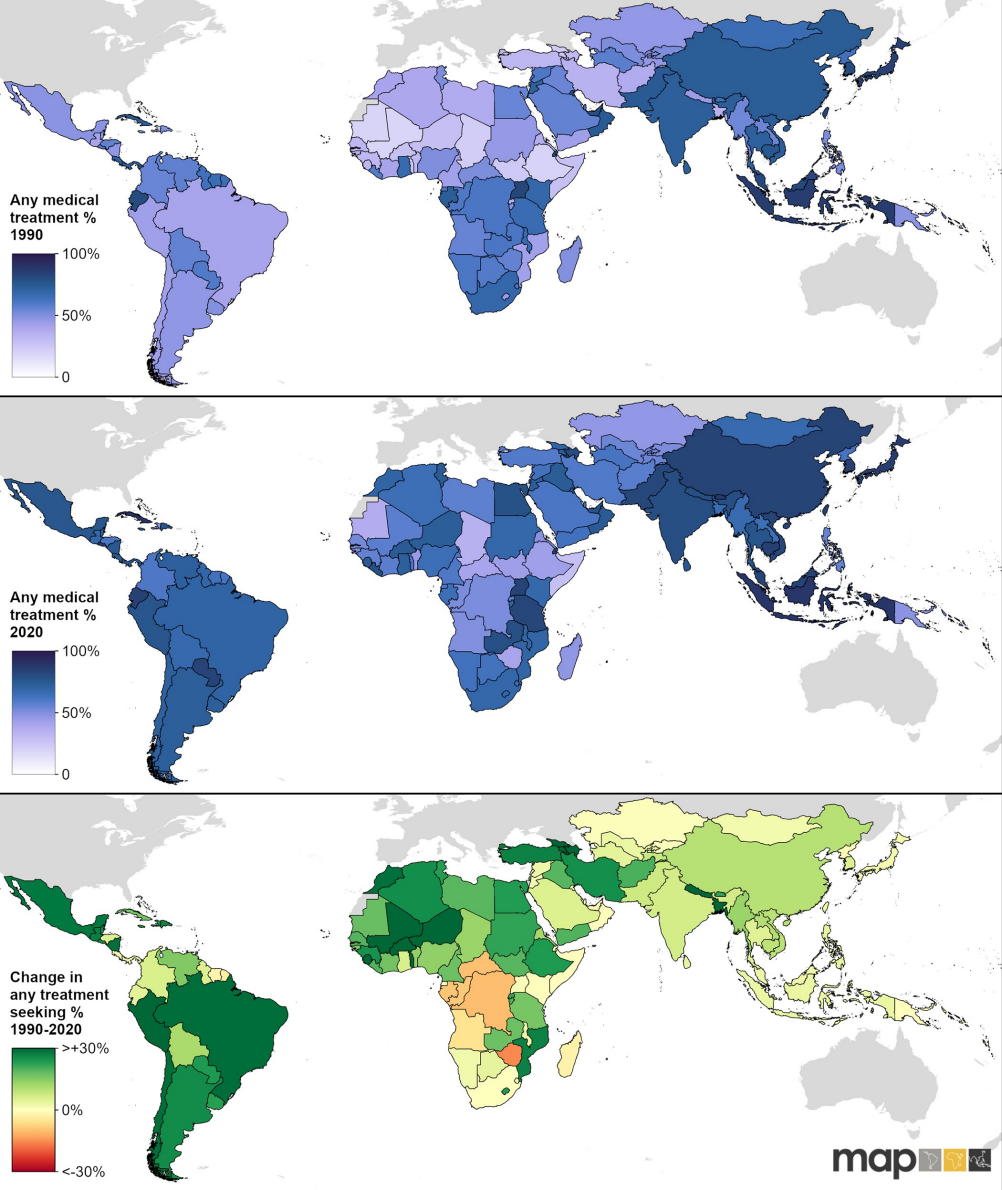
Country-level any medical treatment-seeking rates. Maps showing estimated any medical treatment-seeking rates in 1990 (top panel), 2020 (middle panel), and the absolute change in any medical treatment-seeking rates between 1990 and 2020 (bottom panel). Global national shapefile obtained from the Malaria Atlas Project (MAP; https://malariaatlas.org/) and available for download through the malariaAtlas R package.

Fig 3 illustrates national level treatment rates in 1990 and 2020. From this illustration, we can observe that the countries in the northern parts of Africa were estimated to have the lowest treatment-seeking rates relative to other parts of the world in 1990—particularly Mauritania, Mali, and Ethiopia. Latin America and the Caribbean and Central Asia also stood out as having lower treatment-seeking rates compared to other parts of Asia and Southern Sub-Saharan Africa. We estimate that treatment-seeking was more equitable in 2020. In 1990, 13/151 countries had an estimated mean treatment-seeking rate below 30%, and this proportion dropped to only 1/151 in 2020. However, some North African countries continue to have concerningly low treatment-seeking rates in 2020, in particular Somalia, Mauritania, Chad, Ethiopia, South Sudan and Central African Republic. The lowest overall treatment-seeking rate in 2020 was estimated for Somalia, with a rate of just 28% (19–38). Another concerning finding are countries (20/151) that experienced a decline in treatment-seeking over the study period. Among these, Zimbabwe had the greatest estimated decline from 55% (46–65) in 1990 to 40% (30–49) in 2020.

In 1990, the four countries with the highest estimated treatment-seeking rates were Brunei, Indonesia, Japan, and Singapore, and only 10/151 countries had an estimated mean treatment-seeking rate of over 80% that year. In 2020, the number of countries with a mean treatment-seeking rate of over 80% increased to 22/151. While this list comprised primarily of countries in the Asian region, Cuba had the highest estimated treatment-seeking rate of 89% (83–94).

### Trends in proportion of treatment in the public sector in 1990–2020

The overall population-weighted average for the proportion of medical treatment sought in the public sector did not change much in the study period–it was 49% (42–58 95% CI) in 1990 and 47% (44–52 95% CI) in 2020 (Table 2). However, regionally, we observed significant shifts in terms of preferences of the public sector over the private sector. In particular, in Latin America and the Caribbean, we saw a rise from 54% (45–63) of treatment sought in the public sector in 1990, to 77% (71–81) in 2020, indicating a substantial increase in preference towards the public sector (Fig 2(B), Table 2). In all countries within this region in 2020, the estimated mean fraction of treatment accessed at public facilities was over 50%. In all other regions, preference for the public sector either decreased or stayed relatively unchanged (Fig 2(B), Table 2). The biggest regional decrease was observed in North Africa and the Middle East, where we estimated a drop from 61% (55–68) of care sought in the public sector in 1990 to 44% (38–50) of care sought in the public sector in 2020 (Fig 2(B), Table 2). From Fig 2, we can see that while South Asia was estimated to have relatively high medical treatment rates for fever (Fig 2(A)), it also had the lowest proportion of overall medical treatment accessed at public points of medical care (Fig 2(B)). These findings suggest a strong reliance for seeking healthcare from private sources, which has been reported previously in this region [21]. The proportion of medical attention sought at public health facilities was by far the highest in Central Asia, which we define as consisting of Armenia, Azerbaijan, Georgia, Kazakhstan, Kyrgyzstan, Mongolia, Tajikistan, Turkmenistan, and Uzbekistan. We estimate that the public fraction in this region was 97% (91–99) in 1990 and 95% (88–99) in 2020 (Table 2). In Africa and the Middle East, public treatment for fever was estimated to be more common in 59/68 countries in 1990, and in 53/68 countries in 2020. In Southeast Asia, East Asia, and Oceania, however, public treatment for child fever was more common than treatment in the private sector for 22/35 countries in 2020 –out of those, all 15 countries analysed from Oceania had an estimated public sector fraction of over 80% in 2020, showing a strong public sector preference in contrast with the rest of the region (S2 Fig, S1 Table).

**Fig 4:**
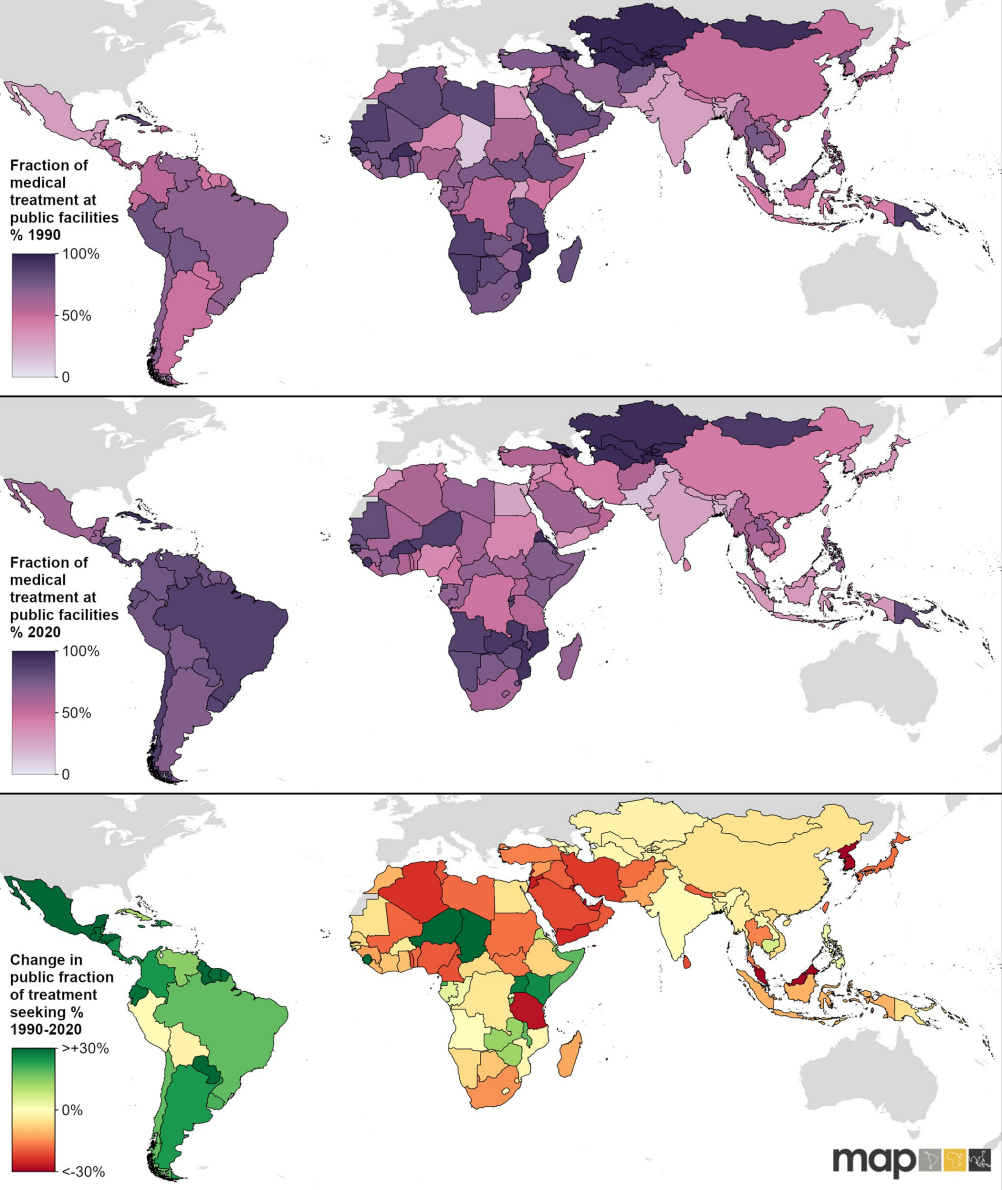
Country-level public sector fraction amongst those seeking medical care. Fraction of medical treatment accessed at public facilities in 1990 (top panel) and 2020 (middle panel) and the absolute change in the public sector utilisation preference between 1990 and 2020 (bottom panel). Global national shapefile obtained from the Malaria Atlas Project (MAP; https://malariaatlas.org/) and available for download through the malariaAtlas R package.

Countries with the lowest fraction of treatment at public facilities in 1990 across the whole study region were Chad, Bangladesh, Uganda, Pakistan, Guatemala, India, and Mexico, which all had public treatment-seeking rates below 30%. In 2020, countries with the fraction of treatment at public facilities below 30% were Pakistan, Bangladesh, Egypt, South Korea, India, and Nepal (Fig 4, S1 Table).

## Discussion

Our analysis of treatment-seeking rates of children under five years old with fever suggests that the treatment-seeking rates have improved substantially over the last 30 years and were much more equitable in 2020 than in 1990. Countries with lowest treatment-seeking rates in 1990 achieved the greatest improvements in treatment-seeking by 2020. There is still much more to be done in terms of equity, however, as we estimated that the highest national treatment-seeking rate (89%, 83–94) was more than triple that of the lowest national treatment-seeking rate (28%, 19–38) in 2020. Furthermore, low or decreasing proportions of treatment-seeking within the public sector in many of the countries is a cause of concern for low-income populations who may struggle to make out-of-pocket payments to access healthcare in the private sector.

Regionally, a few distinct patterns emerged. In Latin America and the Caribbean, treatment-seeking rates started relatively low in the 1990s and experienced a significant increase, with large proportions of treatment-seeking in the public sector. This finding is in line with a strong, region-wide commitment to achieve universal health coverage, which was initiated in the late 1980s [22,23]. The Southeast Asia, East Asia, and Oceania and South Asian regions had the highest total treatment-seeking rates and a strong preference for seeking care for fever in private or informal settings, although Oceania stood out relative to the rest of the region in terms of relatively lower treatment-seeking rates in Papua New Guinea and Solomon Islands, and a stronger preference for treatment in the public sector. The region with the lowest proportions of treatment-seeking from government facilities was South Asia, even though the overall treatment-seeking rates were relatively high in this region. Central Asia had the highest proportion of public treatment-seeking, with over 97% of treatment sought from government facilities throughout the study period. This outcome is likely due to the legacy of most countries in this region being post-Soviet states. The largest drop in the preference of treatment in the public sector was observed in North Africa and the Middle East region, consistent with a report published in 2016 indicating a rapid growth of the private sector in this region, which indicated the shortcomings of the public sector in terms of coverage and quality of care in lower and middle-income countries within the region as a significant factor contributing to this phenomenon [24].

The implications of such patterns at regional and national level are varied and dependent on how interventions within national strategies are approached. For malaria endemic countries, preference for private care could impede control efforts as they may provide less effective or lower quality treatments and are far less likely to confirm malaria cases [25–28]. Sub-optimal treatment regimens and treatment without diagnosis not only draw out the potential for transmission and waste valuable drugs, but also open the door for the potential spread of drug-resistance. In addition, treatment of suspected malaria cases could leave the true cause of fever untreated [29,30].

The national and regional estimates generated from this analysis indicate that the private and informal sectors are an important source of treatment for fevers. In aggregate, it was estimated that 49% (42–58) of care was sought within government run facilities in 1990. This was virtually unchanged in 2020, with an estimate of 47% (44–52). Recent studies indicate that public facilities are more likely to follow national policies for first-line treatment for malaria [31]. Conversely, drug shops and pharmacies may be more affordable, but often provide ineffective monotherapies [32]. A study in Kenya showed that nearly 40% of anti-malarial drugs were distributed by unregistered pharmacies and in Madhya Pradesh, India, 46% of care was sought in unregistered and unregulated informal settings [27,28]. These studies illustrate the need to improve the governance of the private sector healthcare providers in order to achieve adequate surveillance and the universal application of best practices for case management.

Individuals’ rationales for seeking treatment in informal settings or deferring care altogether is likely to vary both across and within countries, but certain factors are consistently identified. Patients will seek care at a centre based on its proximity, the manner of the health care providers, the perceived quality of the medicines and care (i.e., does the patient think they will be cured), timeliness of service, and the cost of care [4]. In our analysis, the fraction of care accessed from public sources was strongly correlated with urbanicity, the number of hospital beds per capita, and the proportion of births delivered at health facilities (Table 1 and S1 Appendix). Making treatment accessible and effective on the provider side is essential as socio-economic status and remoteness are consistently cited as barriers to treatment. Continued investment is therefore needed to bolster treatment options using approaches such as adding points of care (e.g., through the use of community health workers) while maintaining or improving the affordability and quality of treatments [33].

## Limitations

The limitations of our approach include the inherent challenges of relying upon caregiver reporting of both fevers and treatment-seeking behaviours. For example, children’s caregivers might answer “NO” for having sought care for the child’s fever, but then seek it after the interview took place. Furthermore, treatment-seeking for fevers may be low even where health systems are accessible and strong if the majority of fevers are caused by something manageable at home–this is particularly relevant in non-malaria-endemic countries. Nonetheless, evidence from the literature suggests the sensitivity and specificity of fever and treatment recall in household surveys is high enough to warrant continued use of this data source to generate assessments of treatment coverage [34].

Another important limitation is that for countries without data from DHS or MICS programs, estimates relied heavily on the identified covariates and regional trends informed by countries that did have available survey data. For instance, high income countries in the Asia Pacific region (Japan, Brunei, Singapore, and South Korea) did not have any available surveys, and so any differences between estimates in these countries and other countries in this region are driven by differences in the covariates and the modelled relationship between the covariates and the treatment-seeking rates. Finally, due to the small number of surveys after 2019, the estimates for 2020 treatment-seeking rates are likely to not reflect the disruptions to healthcare utilisation caused by the COVID-19 pandemic.

## Conclusion

The results shown here can help determine how best to prioritise policy changes to improve health care access and treatment for fever. Enumerating treatment-seeking rates, trends, and public care preference identifies countries most in need of concerted efforts to improve treatment-seeking for fever. Knowing which health care sectors are preferred in various parts of the world helps determine where limited funding should first be allocated. The temporal trends can also help in assessing the efficacy of measures put in place to improve treatment-seeking. Such assessments could help identify the methods that were most successful in improving treatment-seeking rates, which could inform policies and strategies in other countries. In summary, understanding where quality assured treatment is not being provided or treatment is deferred all together is essential for continued progress in childhood fever management.

## Supporting information

S1 Fig Any treatment plots. Country-level time series of any medical treatment-seeking rates for children under 5 years old.
(PDF)

S2 Fig Public fraction plots. Country-level time series of fraction of treatment in the public sector for children under 5 years old.
(PDF)

S1 Table Country-level results table. Country-level estimates for any medical treatment, public treatment, and public fraction for treatment-seeking of children under 5 years old.
(XLSX)

S2 Table Matched trends and random effects to units without data. (XLSX)

S3 Table List of the surveys included in this study. (CSV)

S1 Appendix Modelling methods. A detailed description of the modelling process employed to derive the estimates presented in this study.
(DOCX)
