# Supplementary material for: Trends in treatment-seeking for fever in children under five years old in 151 countries from 1990 to 2020
Source: PLOS Glob Public Health. 2023 Aug 23;3(8):e0002134. doi: 10.1371/journal.pgph.0002134 (PMC10446233; doi:10.1371/journal.pgph.0002134)
Supplement: S2 Fig — Country-level time series of fraction of treatment in the public sector for children under 5 years old. (PDF) [file pgph.0002134.s002.pdf]

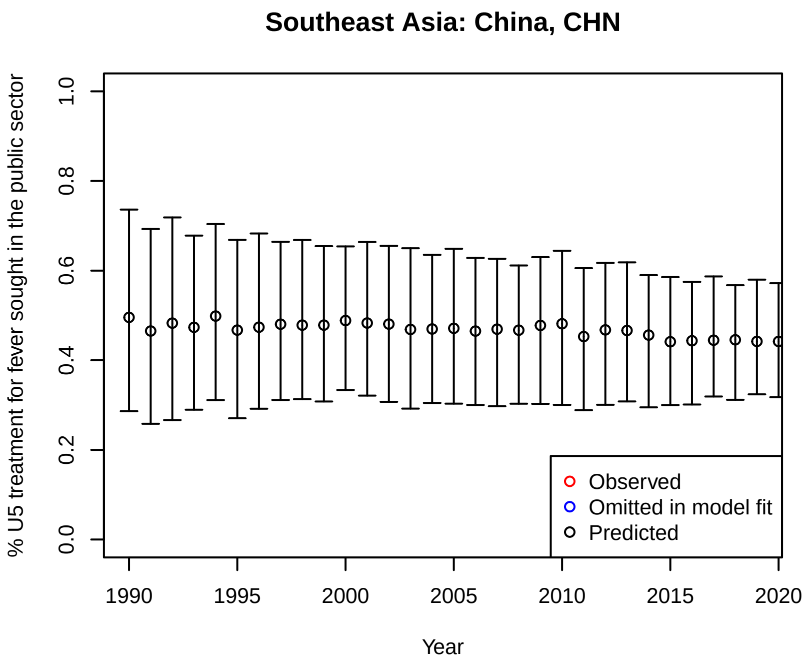
Southeast Asia: China, CHN
% U5 treatment for fever sought in the public sector
0.0
0.2
0.4
0.6
0.8
1.0
1990
1995
2000
2005
2010
2015
2020
Year
Observed
Omitted in model fit
Predicted

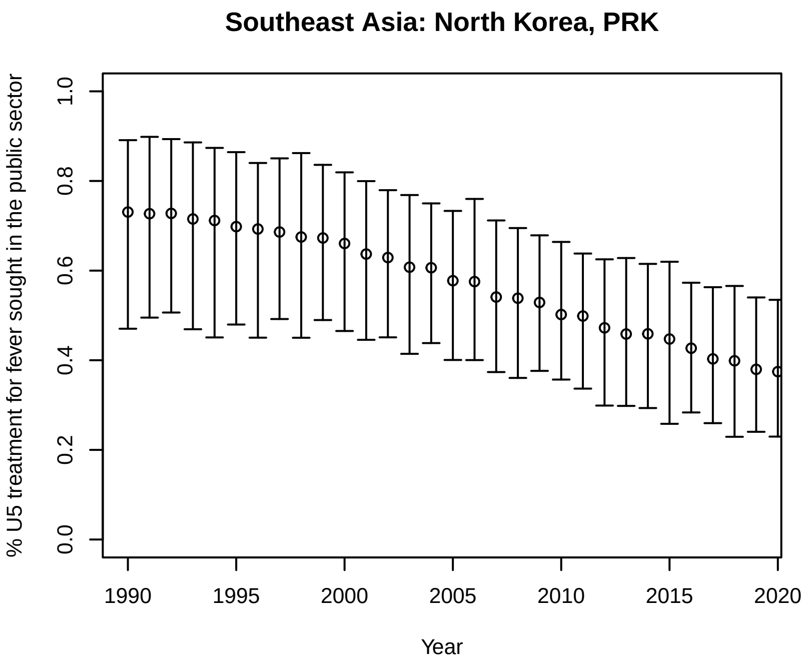
Southeast Asia: North Korea, PRK
% U5 treatment for fever sought in the public sector
0.0
0.2
0.4
0.6
0.8
1.0
1990
1995
2000
2005
2010
2015
2020
Year

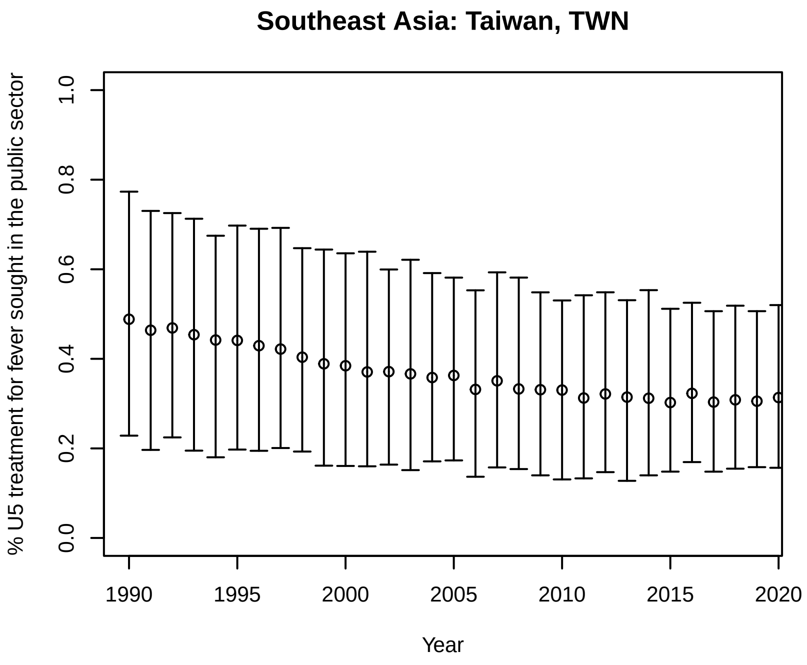
Southeast Asia: Taiwan, TWN
% U5 treatment for fever sought in the public sector
0.0
0.2
0.4
0.6
0.8
1.0
1990
1995
2000
2005
2010
2015
2020
Year

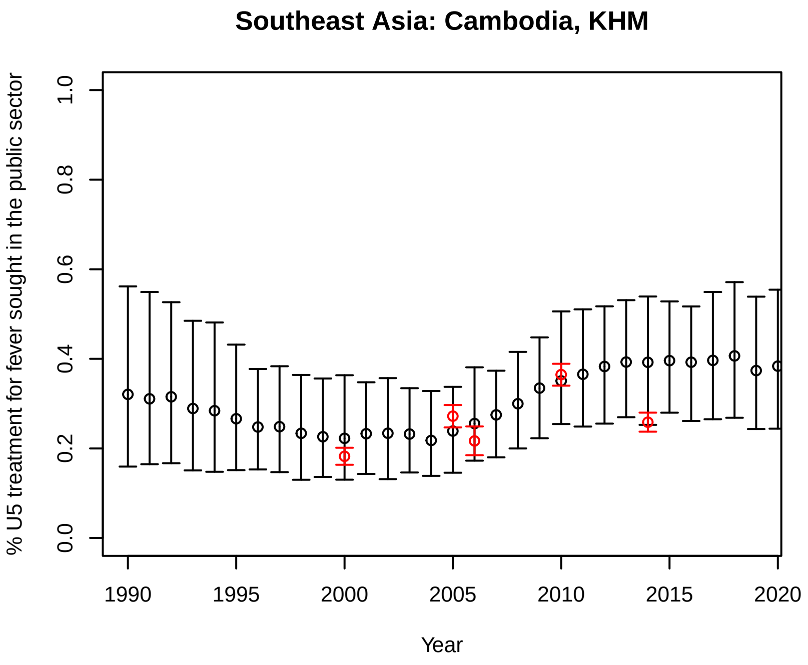
Southeast Asia: Cambodia, KHM
% U5 treatment for fever sought in the public sector
0.0
0.2
0.4
0.6
0.8
1.0
1990
1995
2000
2005
2010
2015
2020
Year

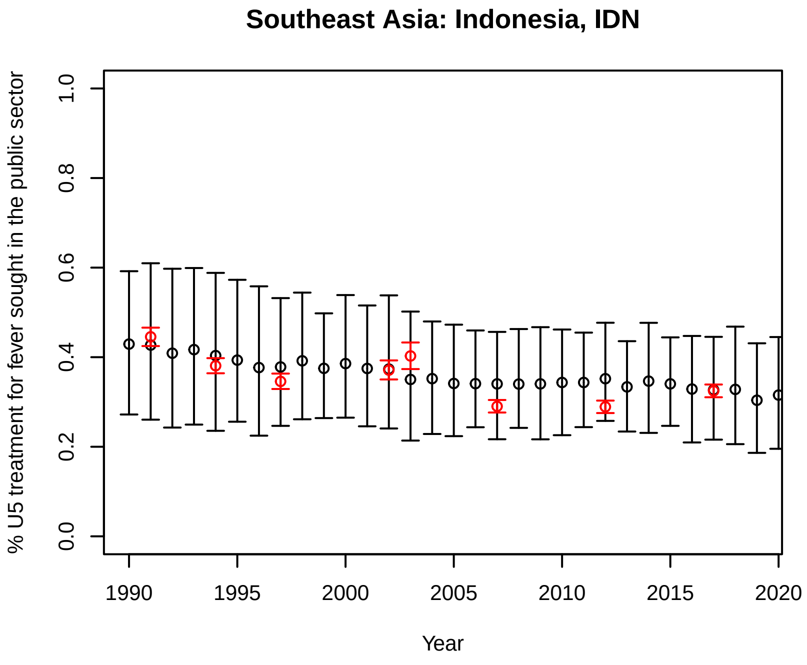
Southeast Asia: Indonesia, IDN
% U5 treatment for fever sought in the public sector
0.0
0.2
0.4
0.6
0.8
1.0
1990
1995
2000
2005
2010
2015
2020
Year

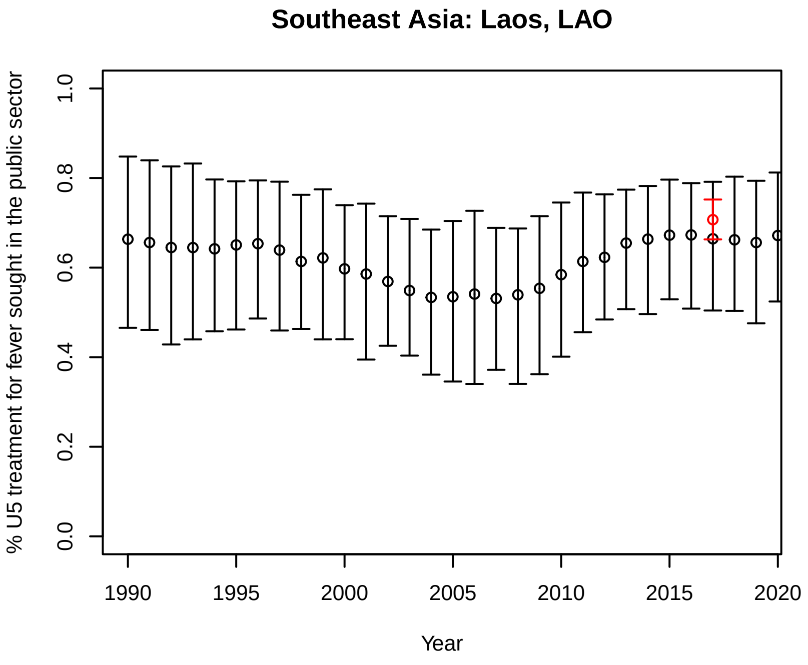
Southeast Asia: Laos, LAO
% U5 treatment for fever sought in the public sector
0.0
0.2
0.4
0.6
0.8
1.0
1990
1995
2000
2005
2010
2015
2020
Year

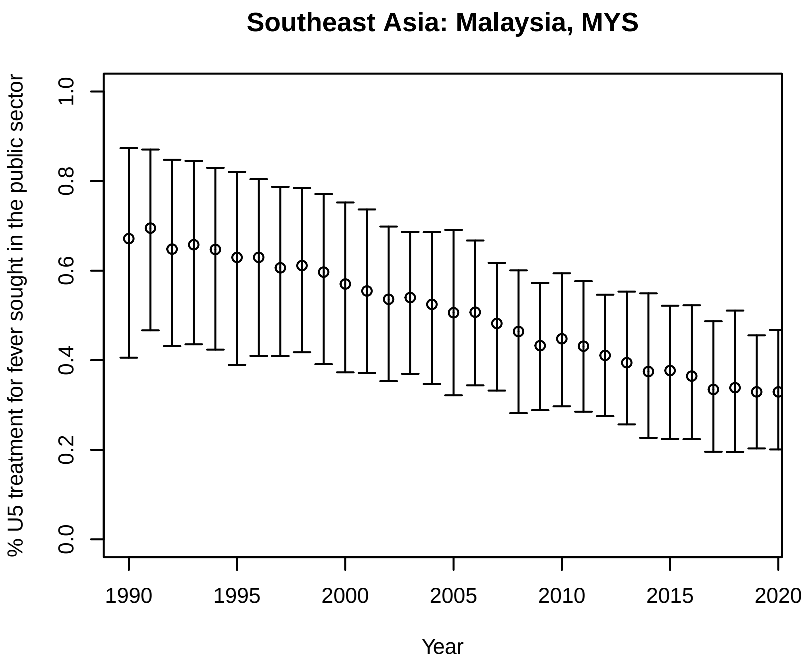
Southeast Asia: Malaysia, MYS
% U5 treatment for fever sought in the public sector
0.0
0.2
0.4
0.6
0.8
1.0
1990
1995
2000
2005
2010
2015
2020
Year

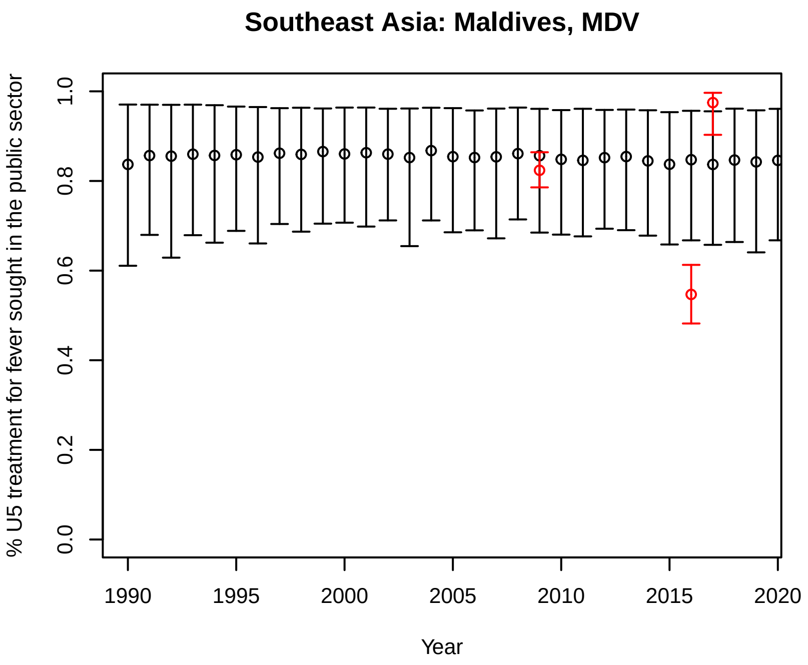
Southeast Asia: Maldives, MDV
% U5 treatment for fever sought in the public sector
0.0
0.2
0.4
0.6
0.8
1.0
1990
1995
2000
2005
2010
2015
2020
Year

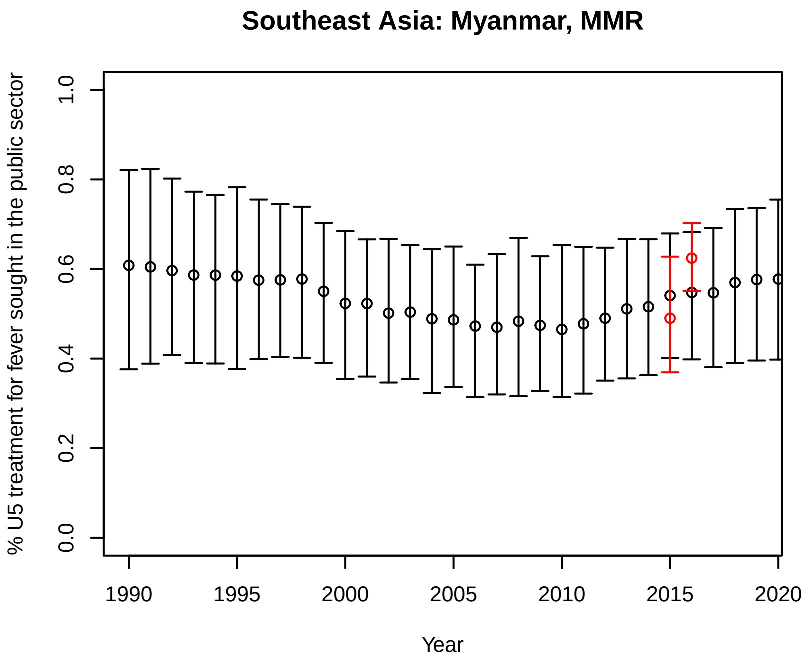
Southeast Asia: Myanmar, MMR
% U5 treatment for fever sought in the public sector
0.0
0.2
0.4
0.6
0.8
1.0
1990
1995
2000
2005
2010
2015
2020
Year

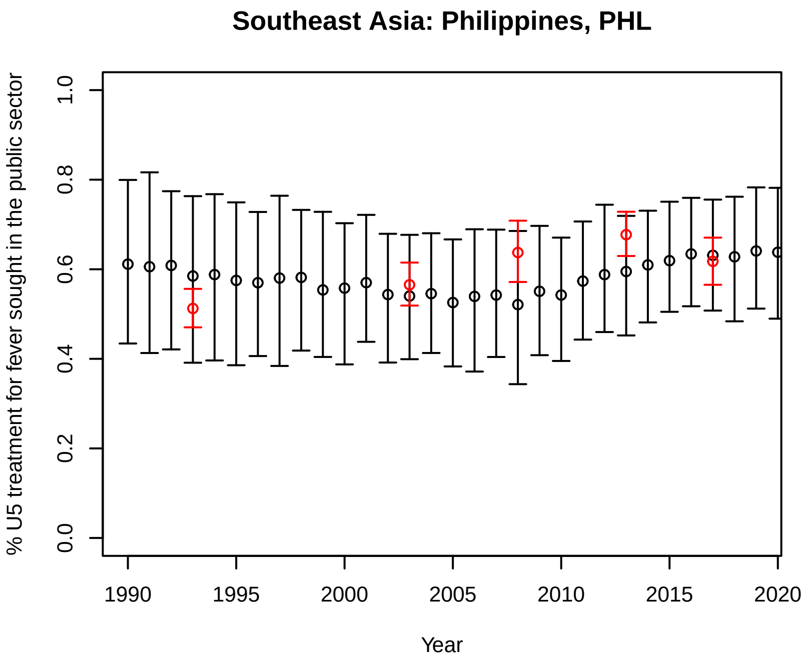
Southeast Asia: Philippines, PHL
% U5 treatment for fever sought in the public sector
0.0
0.2
0.4
0.6
0.8
1.0
1990
1995
2000
2005
2010
2015
2020
Year

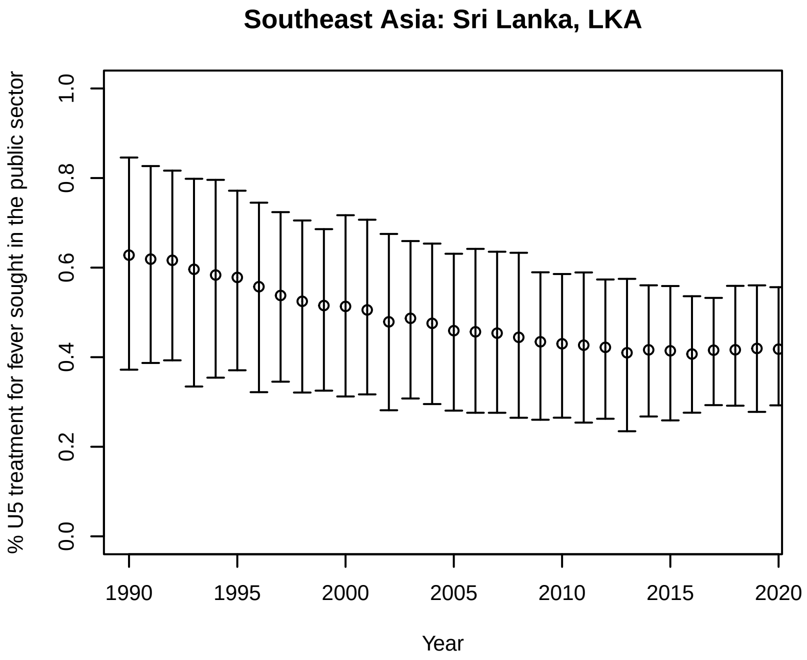
Southeast Asia: Sri Lanka, LKA
% U5 treatment for fever sought in the public sector
0.0
0.2
0.4
0.6
0.8
1.0
1990
1995
2000
2005
2010
2015
2020
Year

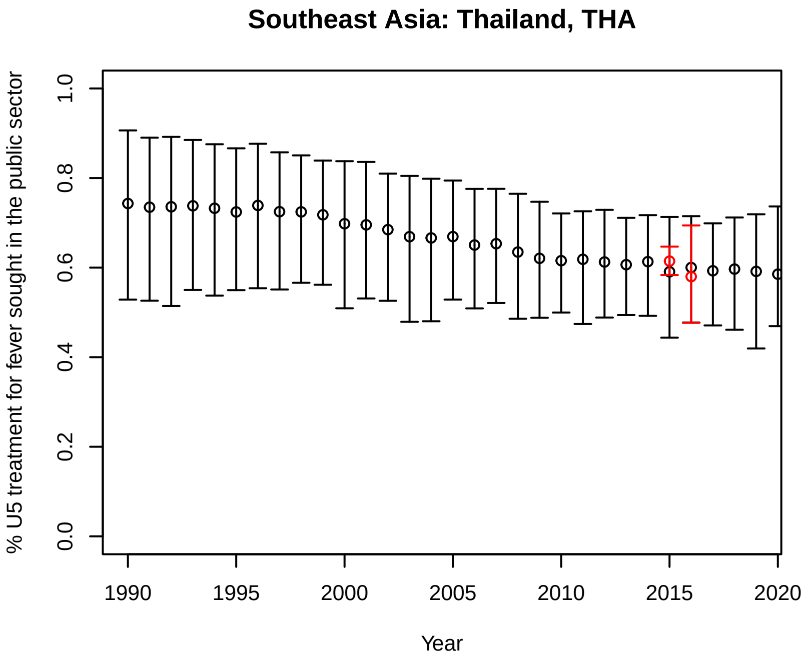
Southeast Asia: Thailand, THA
% U5 treatment for fever sought in the public sector
0.0
0.2
0.4
0.6
0.8
1.0
1990
1995
2000
2005
2010
2015
2020
Year

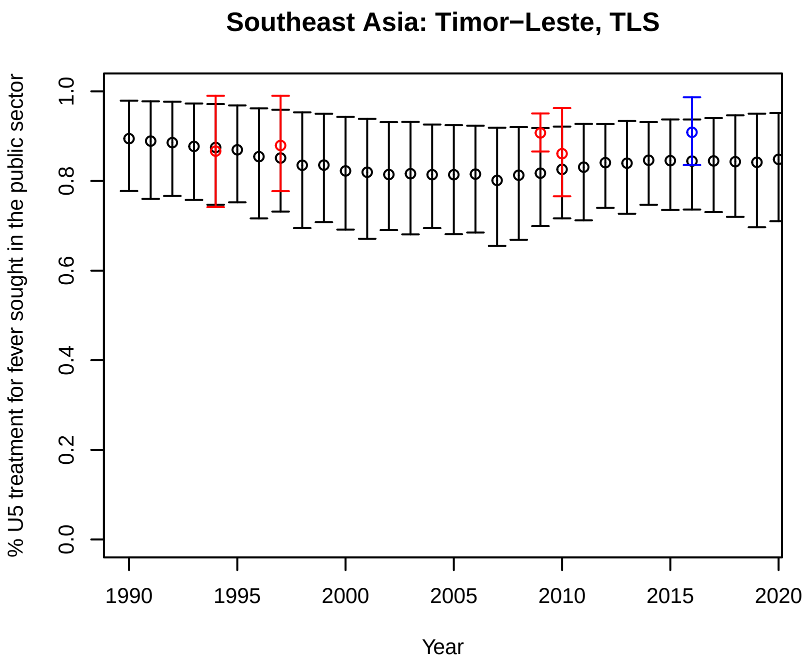
Southeast Asia: Timor−Leste, TLS
% U5 treatment for fever sought in the public sector
Year
0.0
0.2
0.4
0.6
0.8
1.0
1990
1995
2000
2005
2010
2015
2020

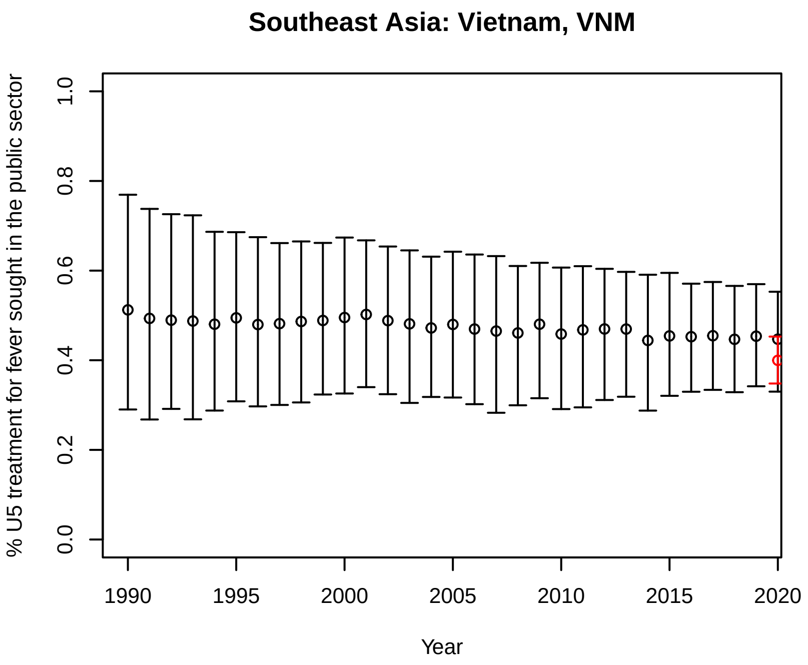
Southeast Asia: Vietnam, VNM
% U5 treatment for fever sought in the public sector
Year
0.0
0.2
0.4
0.6
0.8
1.0
1990
1995
2000
2005
2010
2015
2020

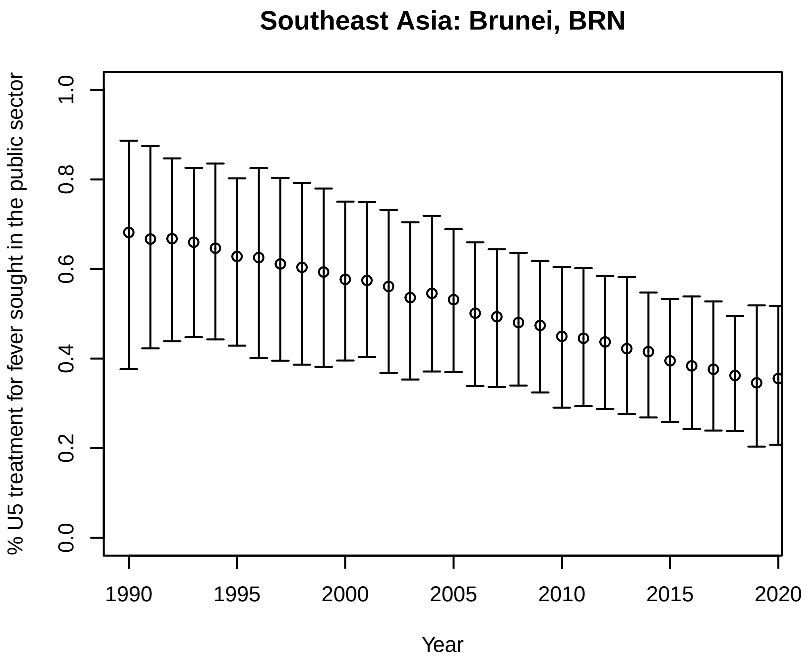
Southeast Asia: Brunei, BRN
% U5 treatment for fever sought in the public sector
Year
0.0
0.2
0.4
0.6
0.8
1.0
1990
1995
2000
2005
2010
2015
2020

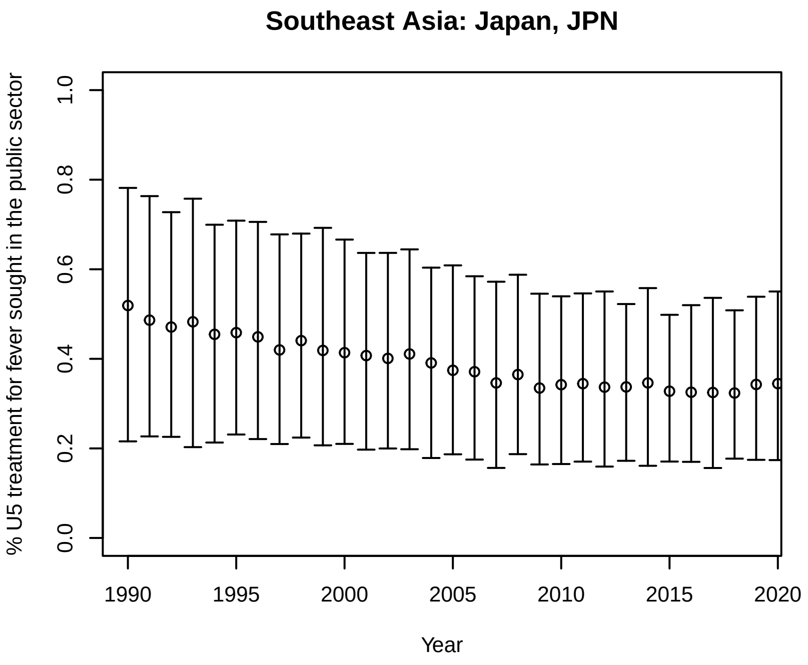
Southeast Asia: Japan, JPN
% U5 treatment for fever sought in the public sector
Year
0.0
0.2
0.4
0.6
0.8
1.0
1990
1995
2000
2005
2010
2015
2020

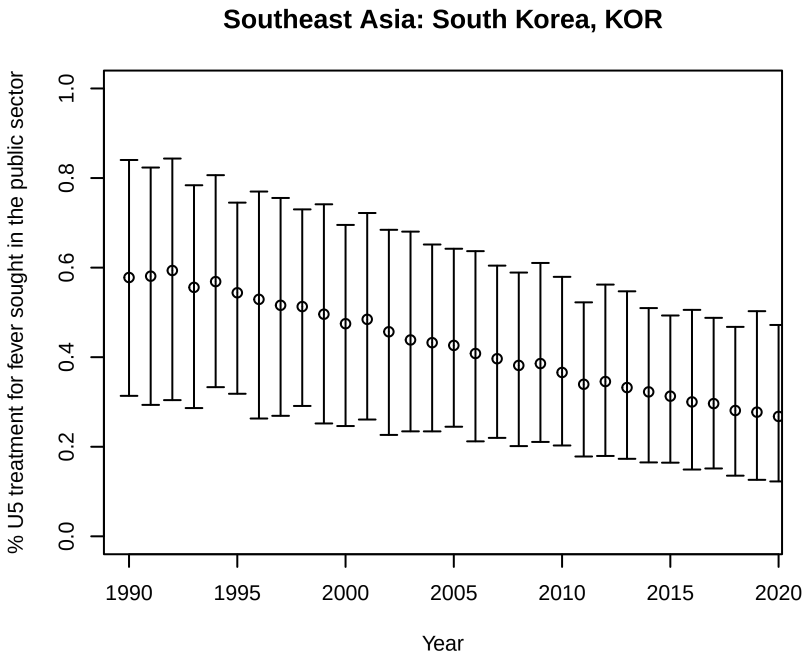
Southeast Asia: South Korea, KOR
% U5 treatment for fever sought in the public sector
Year
0.0
0.2
0.4
0.6
0.8
1.0
1990
1995
2000
2005
2010
2015
2020

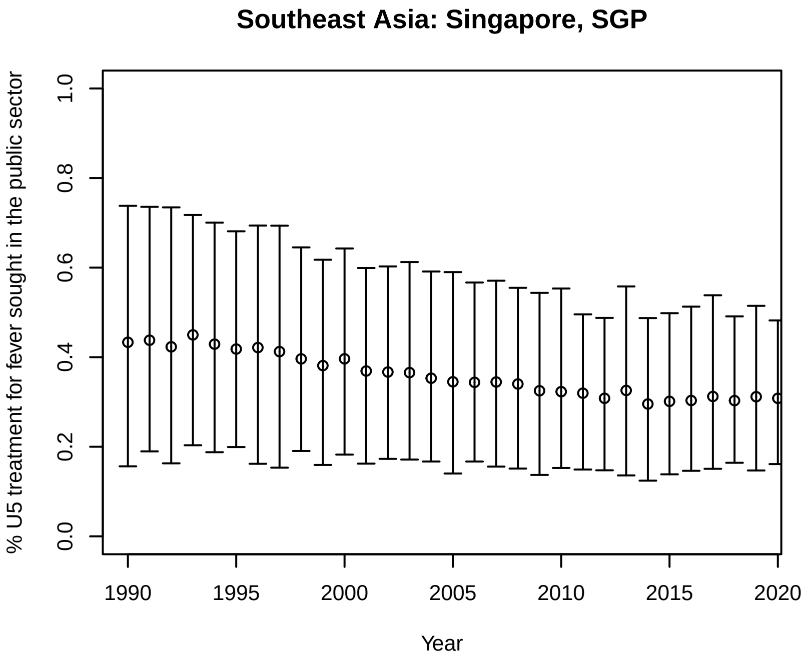
Southeast Asia: Singapore, SGP
% U5 treatment for fever sought in the public sector
Year
0.0
0.2
0.4
0.6
0.8
1.0
1990
1995
2000
2005
2010
2015
2020

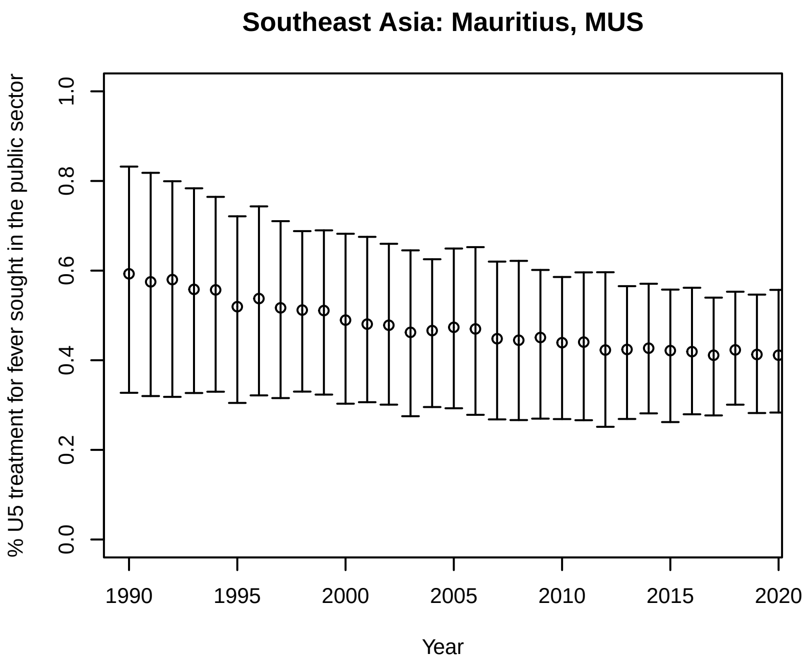
Southeast Asia: Mauritius, MUS
% U5 treatment for fever sought in the public sector
Year

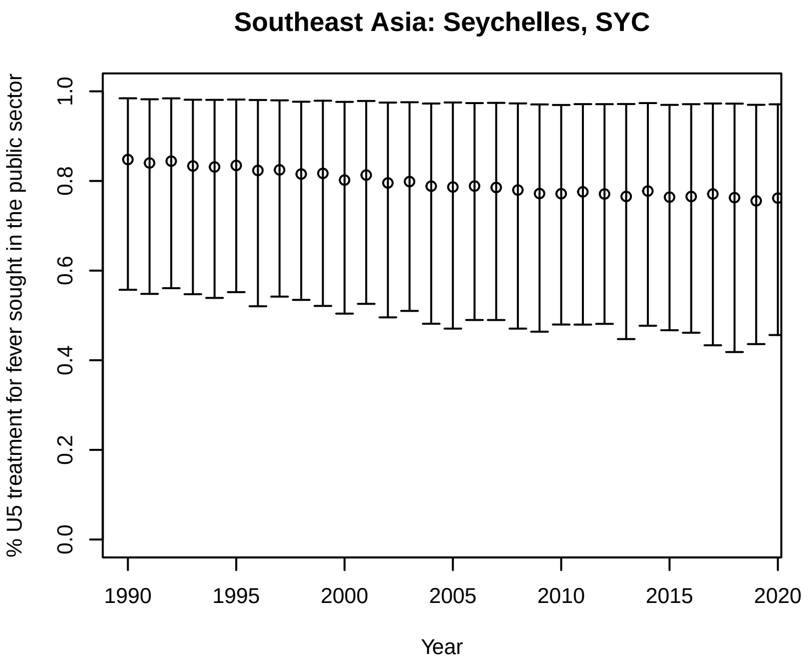
Southeast Asia: Seychelles, SYC
% U5 treatment for fever sought in the public sector
Year

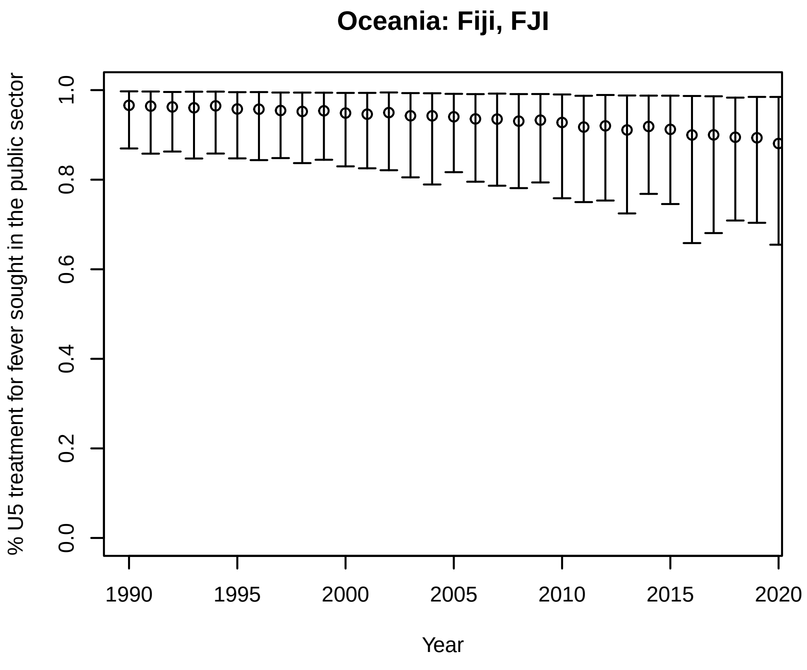
Oceania: Fiji, FJI
% U5 treatment for fever sought in the public sector
Year

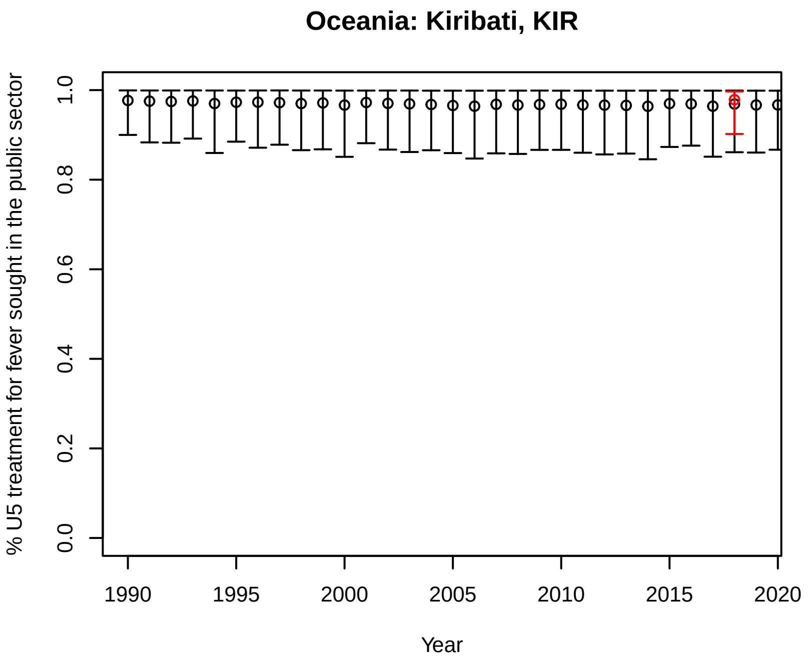
Oceania: Kiribati, KIR
% U5 treatment for fever sought in the public sector
Year

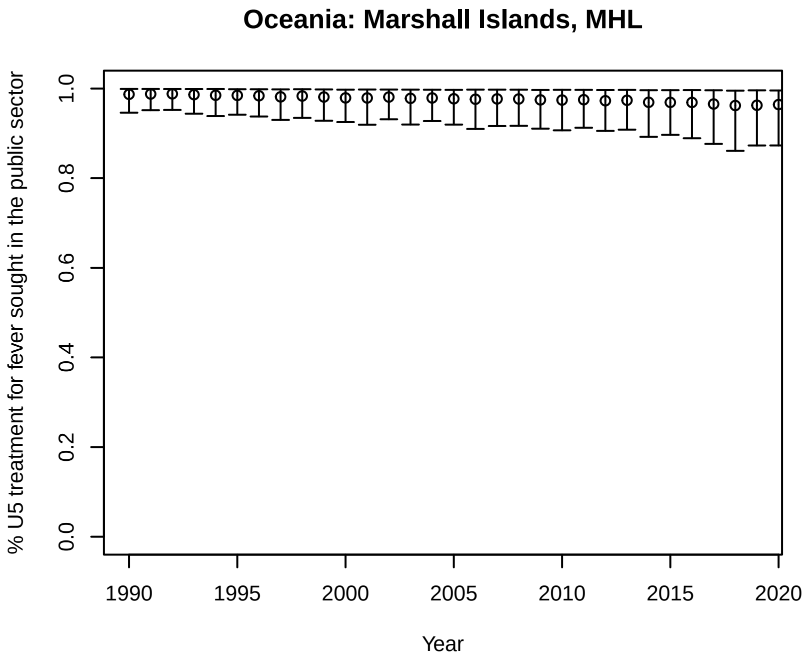
Oceania: Marshall Islands, MHL
% U5 treatment for fever sought in the public sector
Year

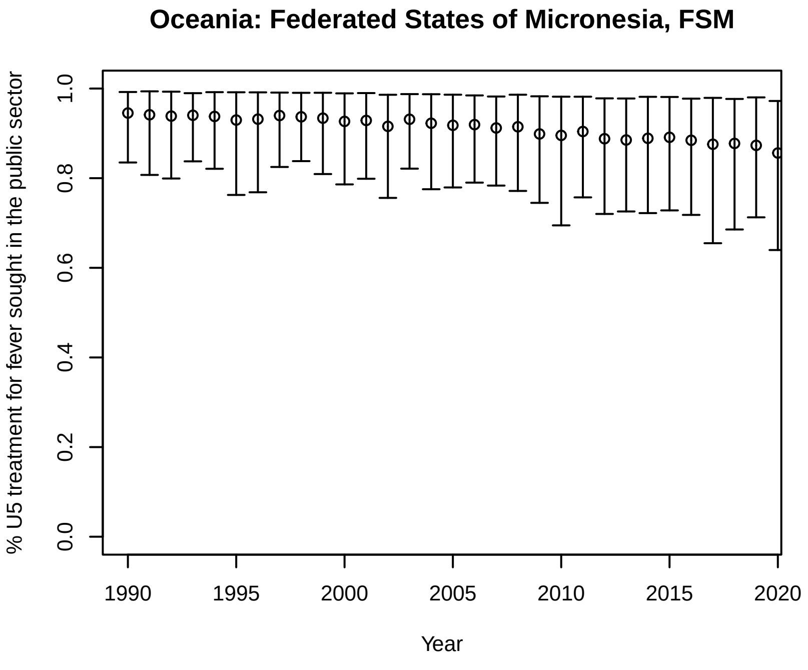
Oceania: Federated States of Micronesia, FSM
% U5 treatment for fever sought in the public sector
Year

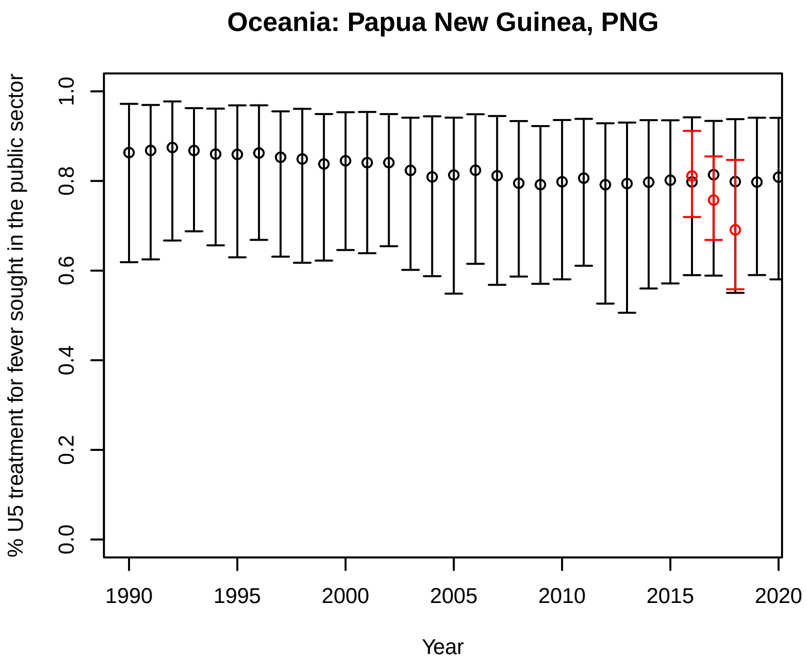
Oceania: Papua New Guinea, PNG
% U5 treatment for fever sought in the public sector
Year
0.0
0.2
0.4
0.6
0.8
1.0
1990
1995
2000
2005
2010
2015
2020

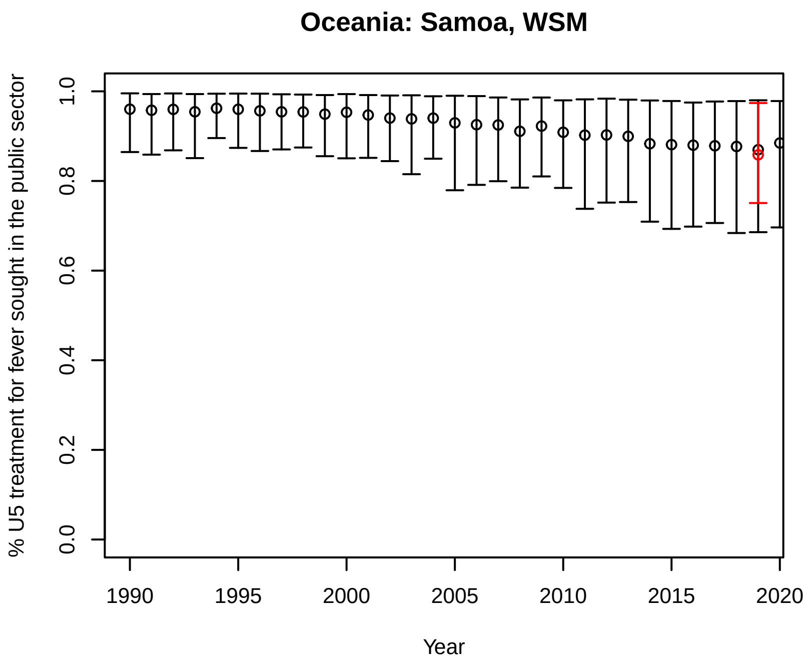
Oceania: Samoa, WSM
% U5 treatment for fever sought in the public sector
Year
0.0
0.2
0.4
0.6
0.8
1.0
1990
1995
2000
2005
2010
2015
2020

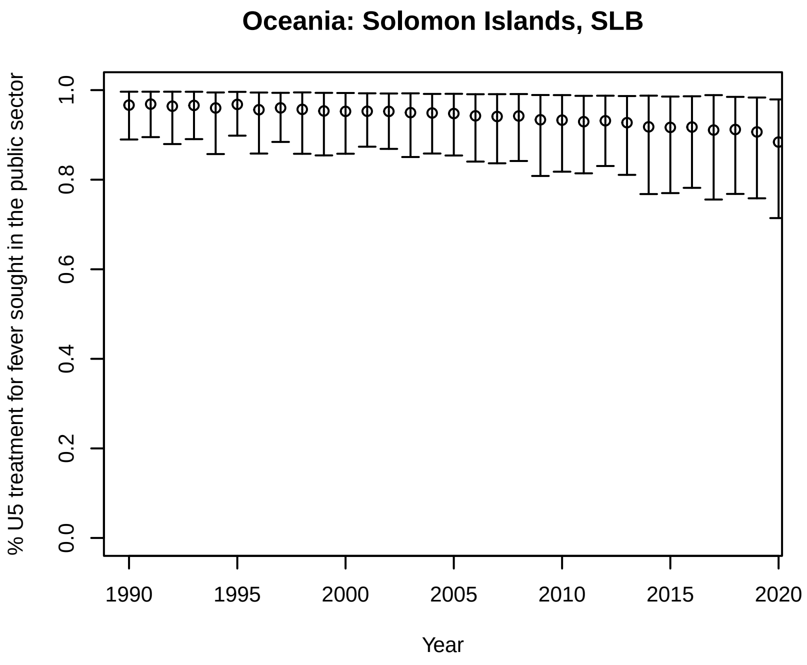
Oceania: Solomon Islands, SLB
% U5 treatment for fever sought in the public sector
Year
0.0
0.2
0.4
0.6
0.8
1.0
1990
1995
2000
2005
2010
2015
2020

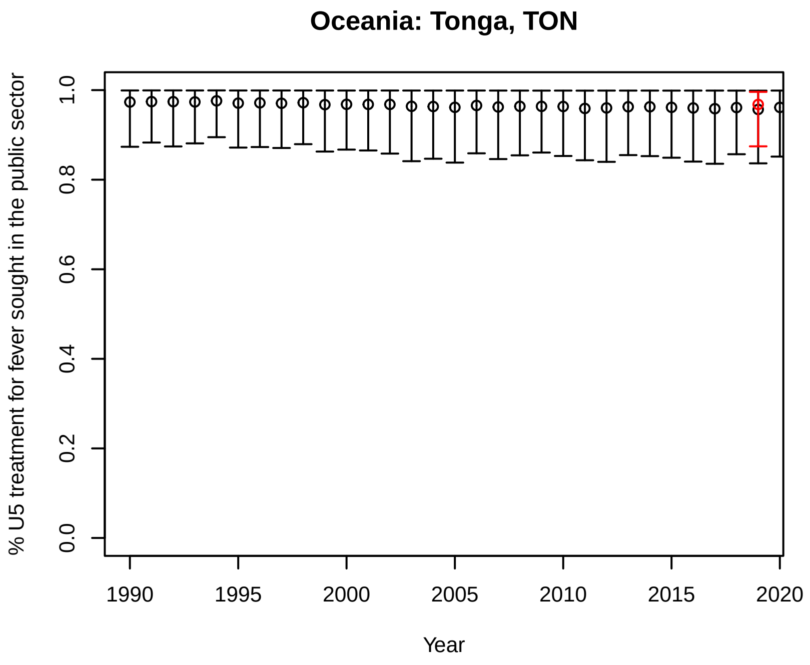
Oceania: Tonga, TON
% U5 treatment for fever sought in the public sector
Year
0.0
0.2
0.4
0.6
0.8
1.0
1990
1995
2000
2005
2010
2015
2020

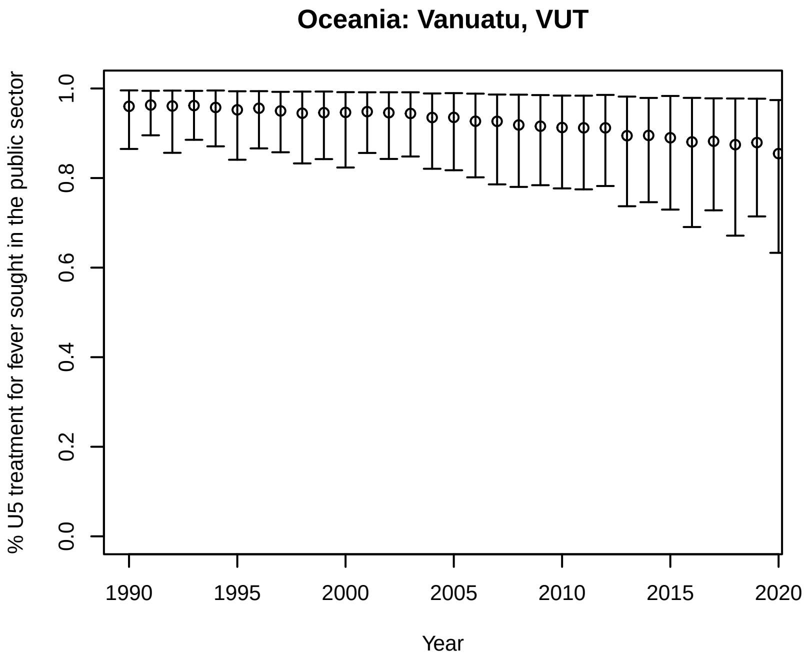
Oceania: Vanuatu, VUT
% U5 treatment for fever sought in the public sector
Year
0.0
0.2
0.4
0.6
0.8
1.0
1990
1995
2000
2005
2010
2015
2020

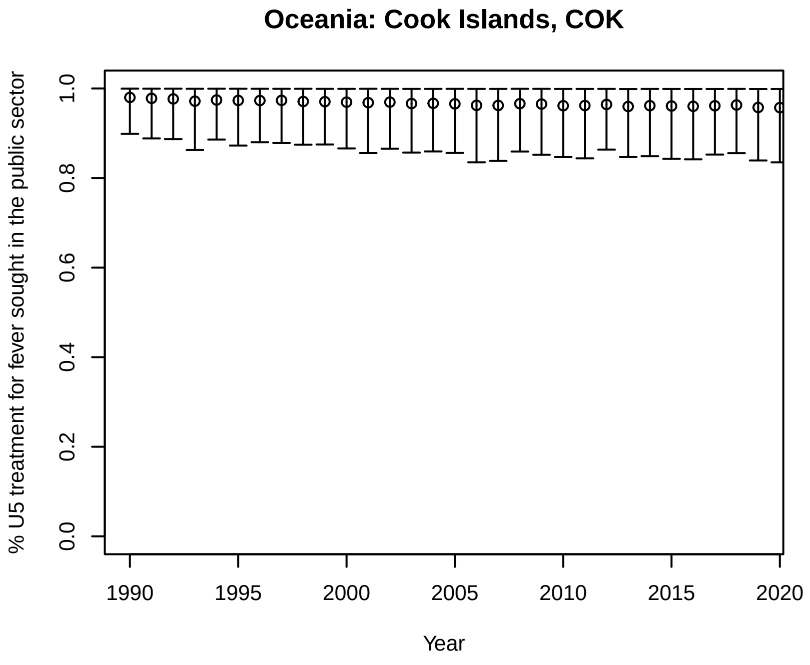
Oceania: Cook Islands, COK
% U5 treatment for fever sought in the public sector
Year
0.0
0.2
0.4
0.6
0.8
1.0
1990
1995
2000
2005
2010
2015
2020

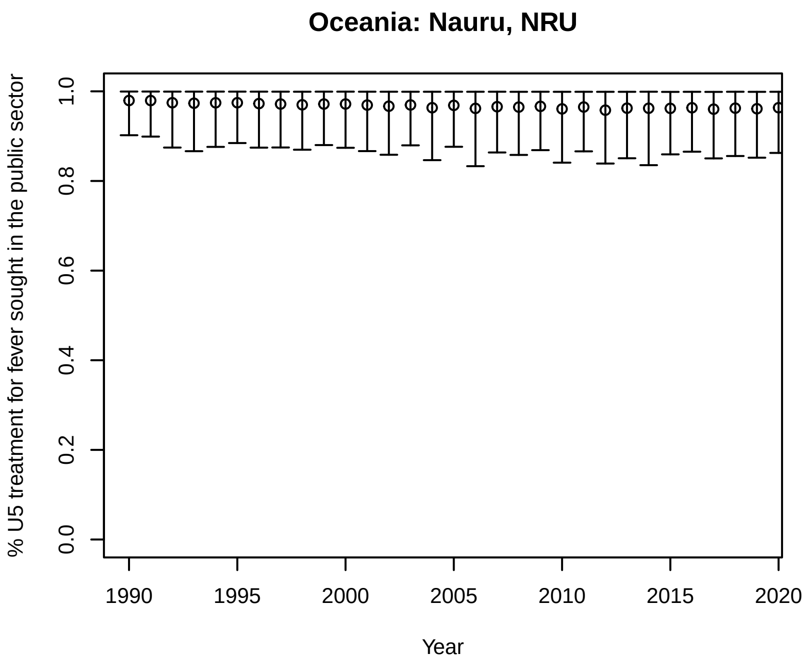
Oceania: Nauru, NRU
% U5 treatment for fever sought in the public sector
0.0
0.2
0.4
0.6
0.8
1.0
1990
1995
2000
2005
2010
2015
2020
Year

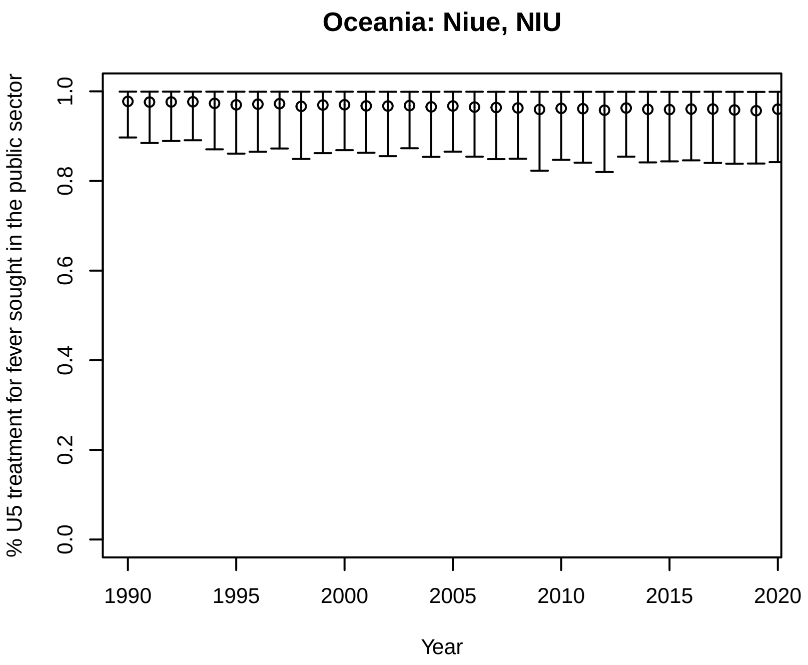
Oceania: Niue, NIU
% U5 treatment for fever sought in the public sector
0.0
0.2
0.4
0.6
0.8
1.0
1990
1995
2000
2005
2010
2015
2020
Year

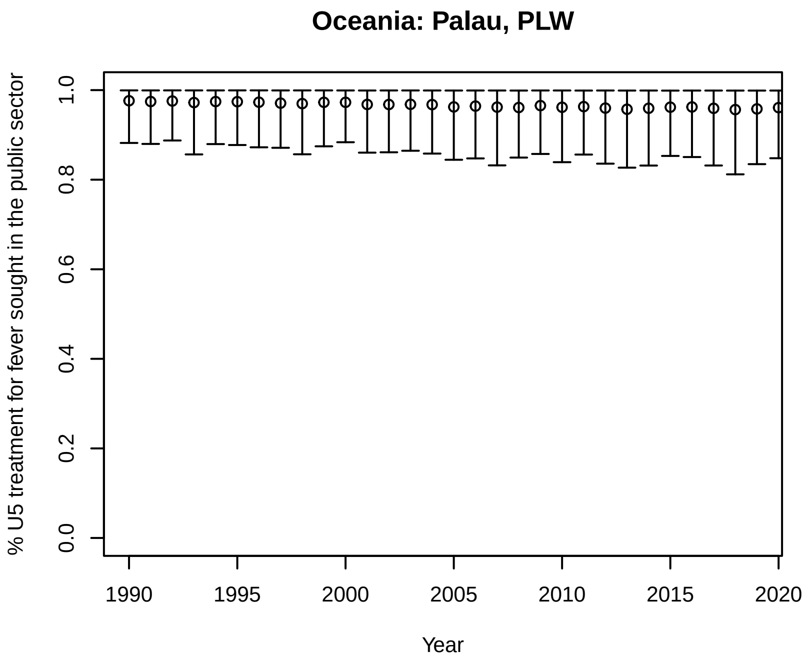
Oceania: Palau, PLW
% U5 treatment for fever sought in the public sector
0.0
0.2
0.4
0.6
0.8
1.0
1990
1995
2000
2005
2010
2015
2020
Year

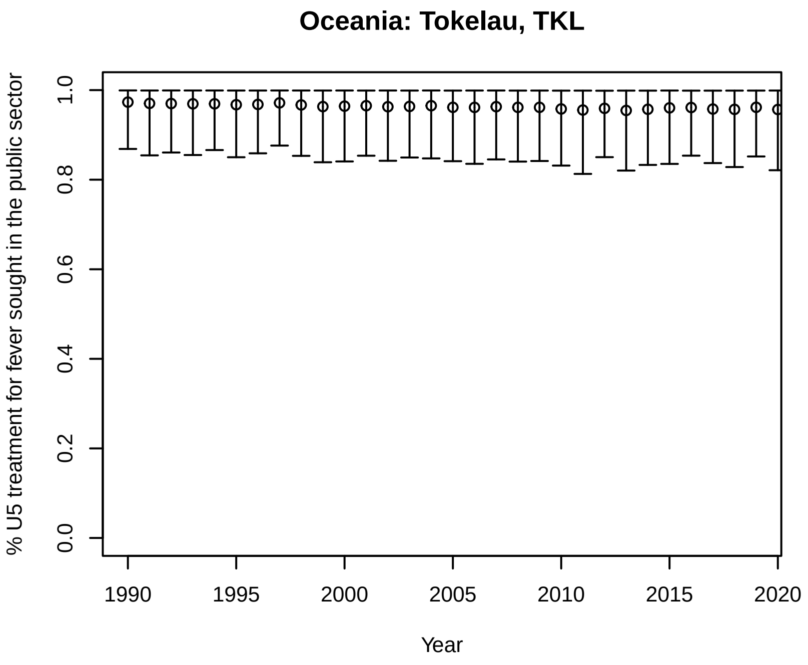
Oceania: Tokelau, TKL
% U5 treatment for fever sought in the public sector
0.0
0.2
0.4
0.6
0.8
1.0
1990
1995
2000
2005
2010
2015
2020
Year

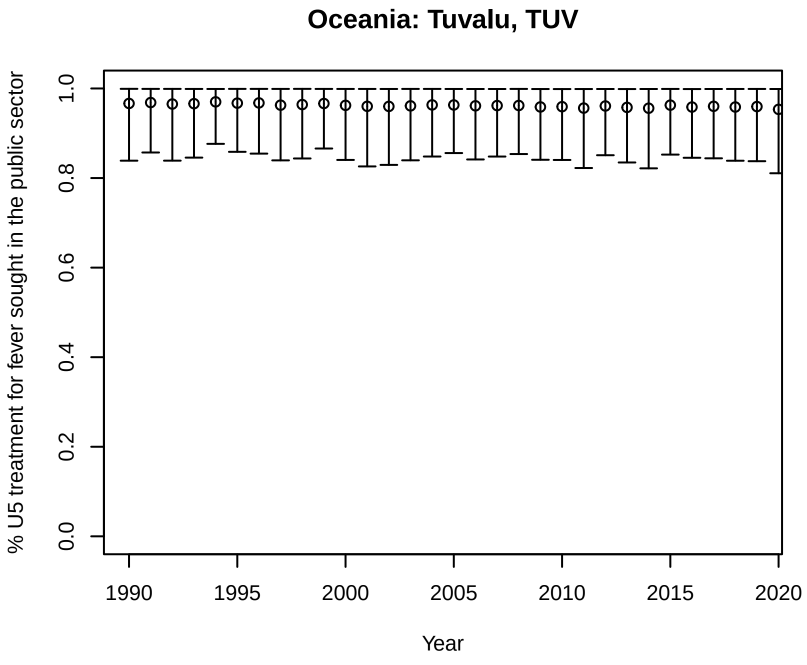
Oceania: Tuvalu, TUV
% U5 treatment for fever sought in the public sector
0.0
0.2
0.4
0.6
0.8
1.0
1990
1995
2000
2005
2010
2015
2020
Year

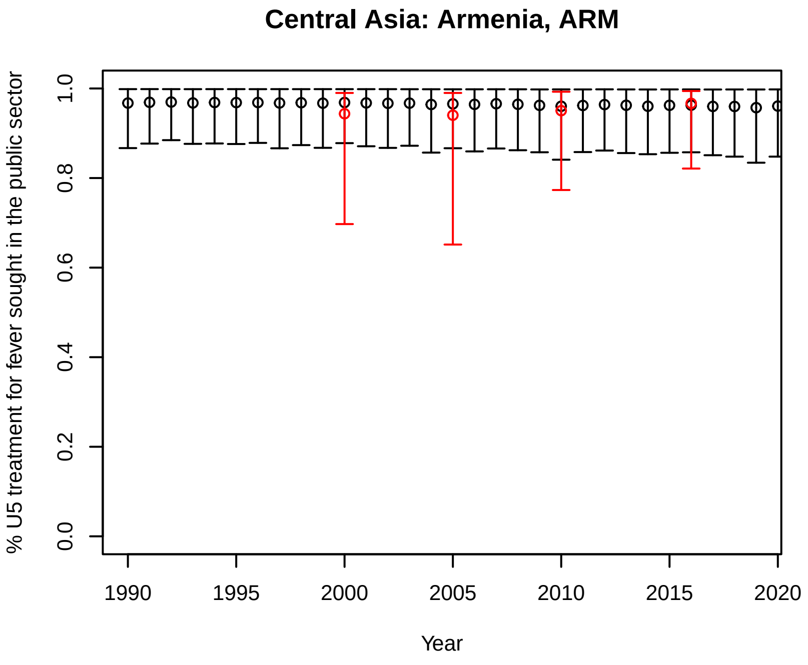
Central Asia: Armenia, ARM
% U5 treatment for fever sought in the public sector
0.0
0.2
0.4
0.6
0.8
1.0
1990
1995
2000
2005
2010
2015
2020
Year

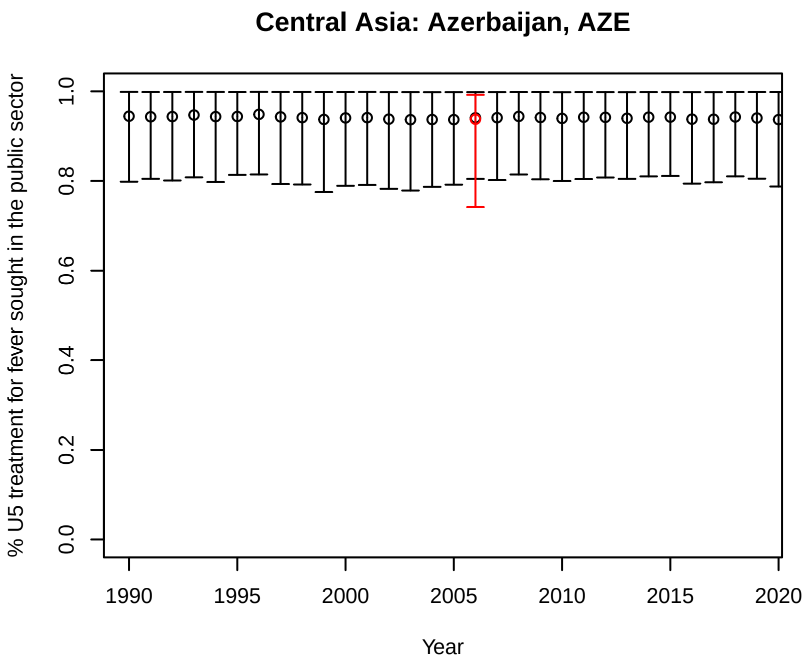
Central Asia: Azerbaijan, AZE
% U5 treatment for fever sought in the public sector
Year

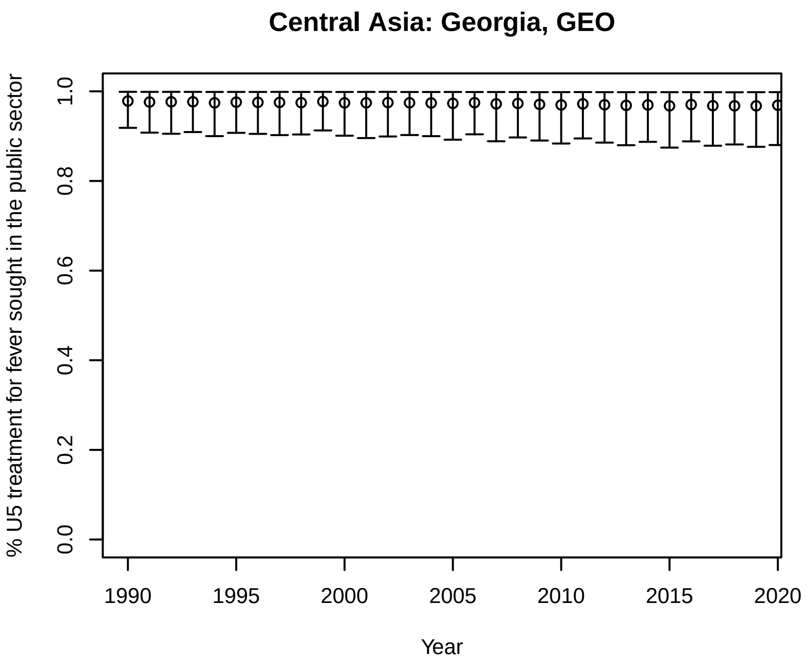
Central Asia: Georgia, GEO
% U5 treatment for fever sought in the public sector
Year

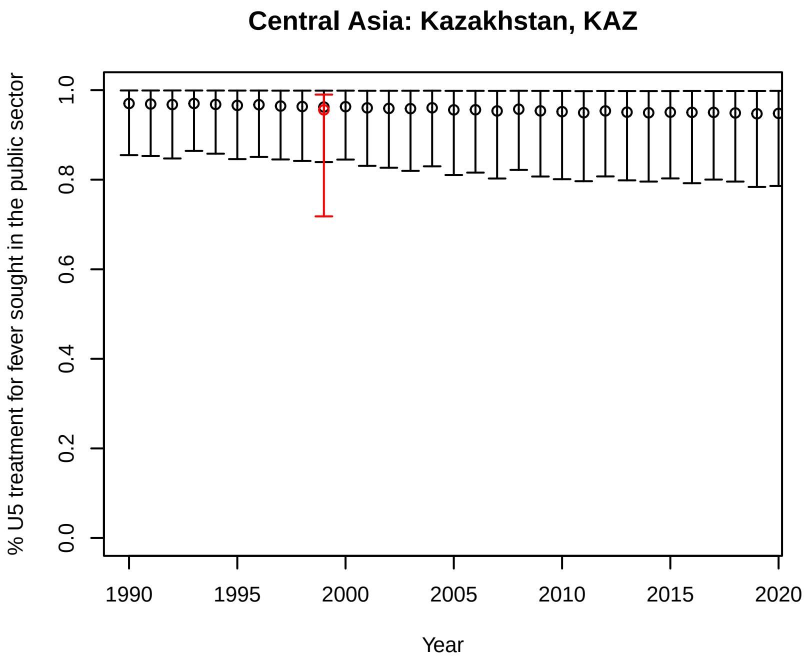
Central Asia: Kazakhstan, KAZ
% U5 treatment for fever sought in the public sector
Year

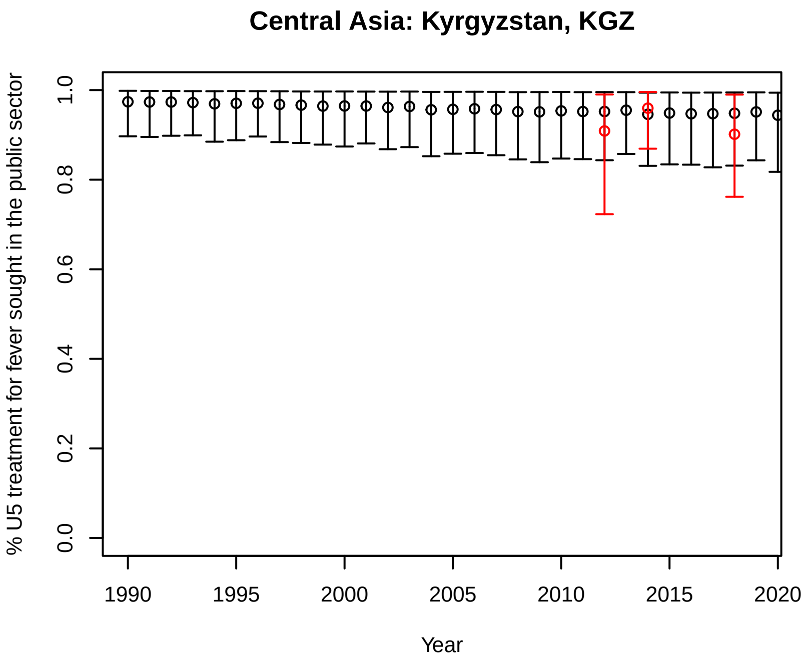
Central Asia: Kyrgyzstan, KGZ
% U5 treatment for fever sought in the public sector
Year

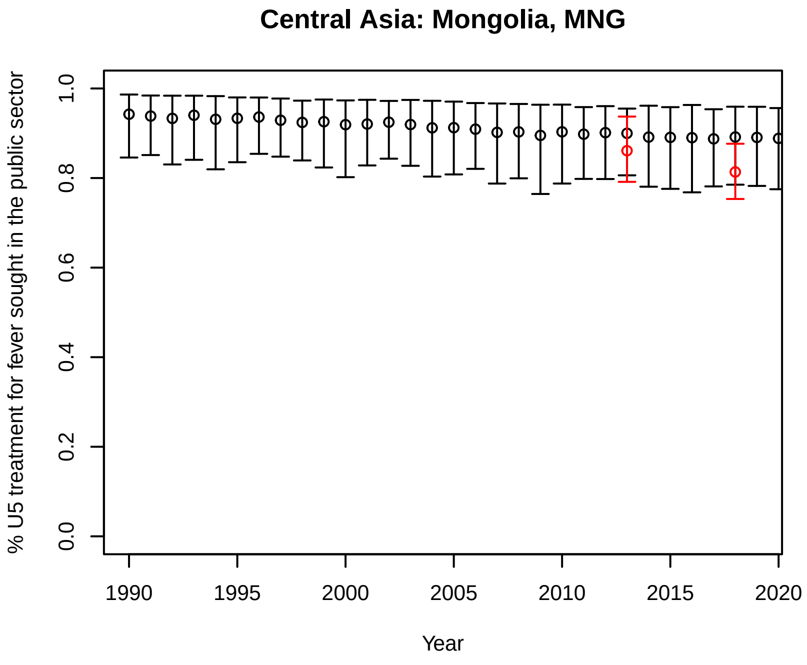
Central Asia: Mongolia, MNG
% U5 treatment for fever sought in the public sector
Year

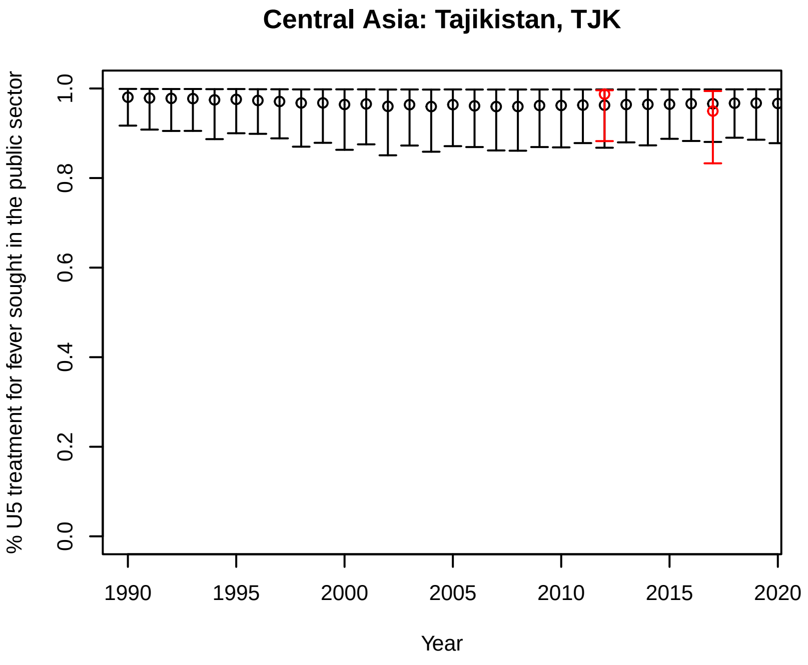
Central Asia: Tajikistan, TJK
% U5 treatment for fever sought in the public sector
Year

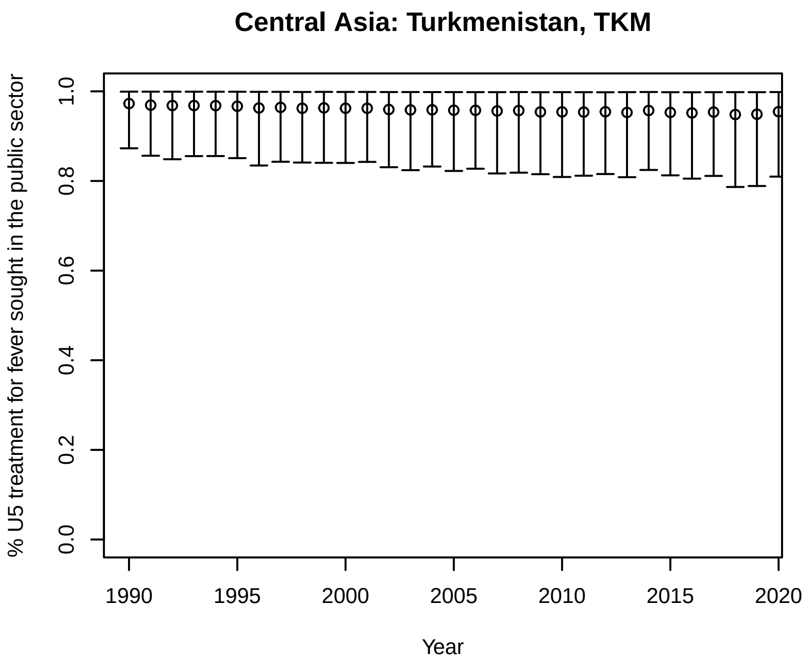

Central Asia: Turkmenistan, TKM
% U5 treatment for fever sought in the public sector
Year

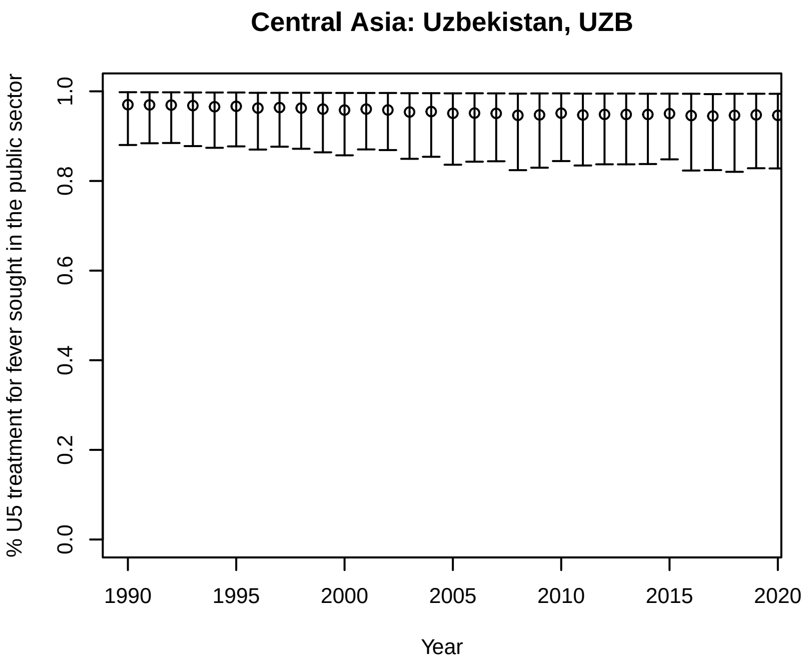

Central Asia: Uzbekistan, UZB
% U5 treatment for fever sought in the public sector
Year

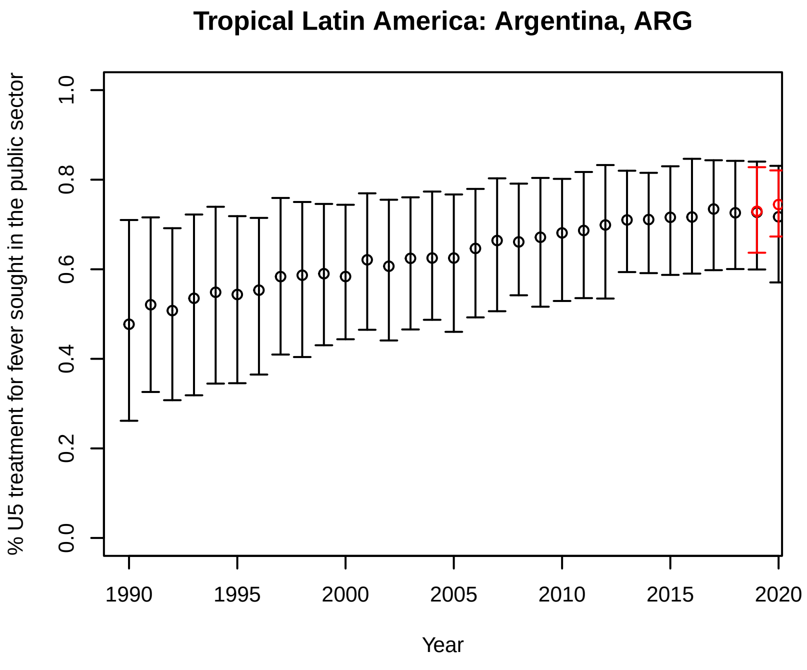

Tropical Latin America: Argentina, ARG
% U5 treatment for fever sought in the public sector
Year

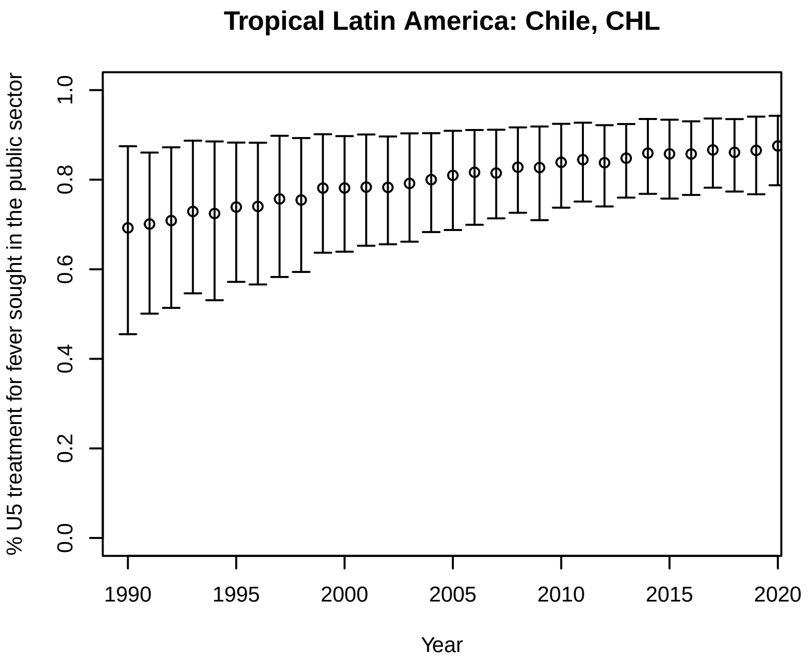

Tropical Latin America: Chile, CHL
% U5 treatment for fever sought in the public sector
Year

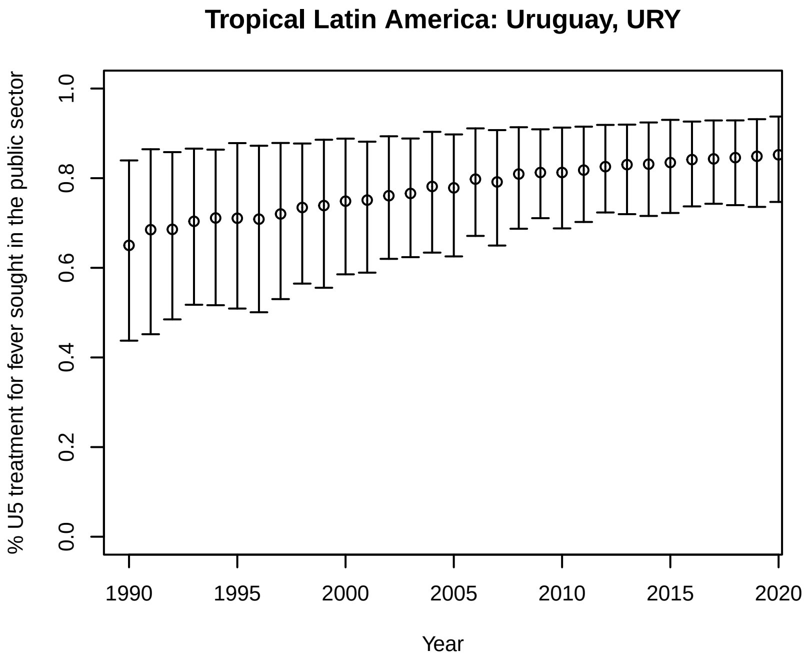

Tropical Latin America: Uruguay, URY
% U5 treatment for fever sought in the public sector
Year

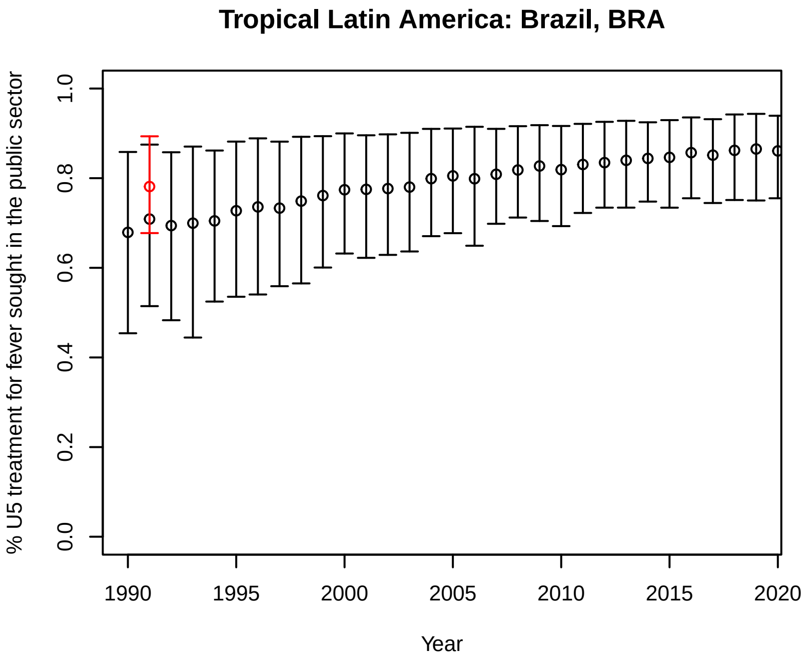

Tropical Latin America: Brazil, BRA
% U5 treatment for fever sought in the public sector
Year

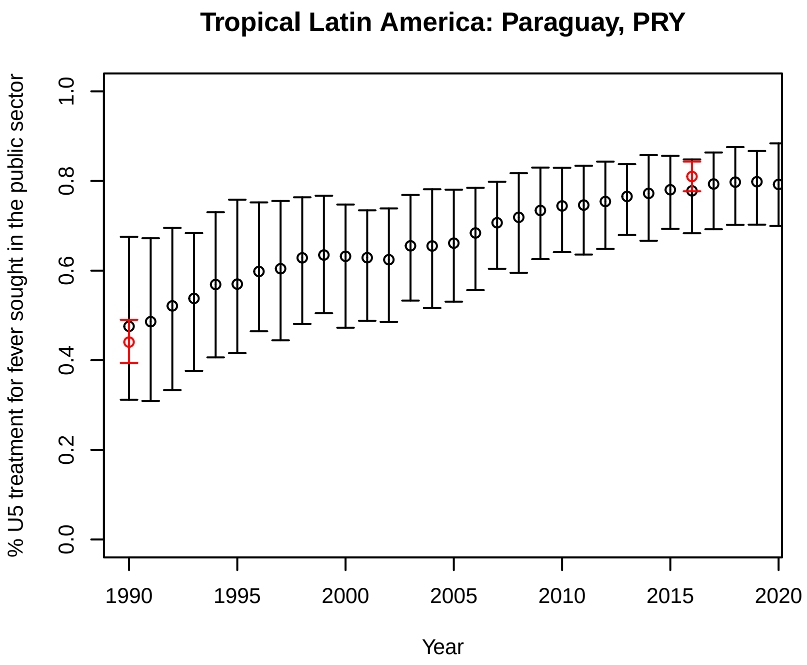
Tropical Latin America: Paraguay, PRY
% U5 treatment for fever sought in the public sector
0.0 0.2 0.4 0.6 0.8 1.0
1990 1995 2000 2005 2010 2015 2020
Year

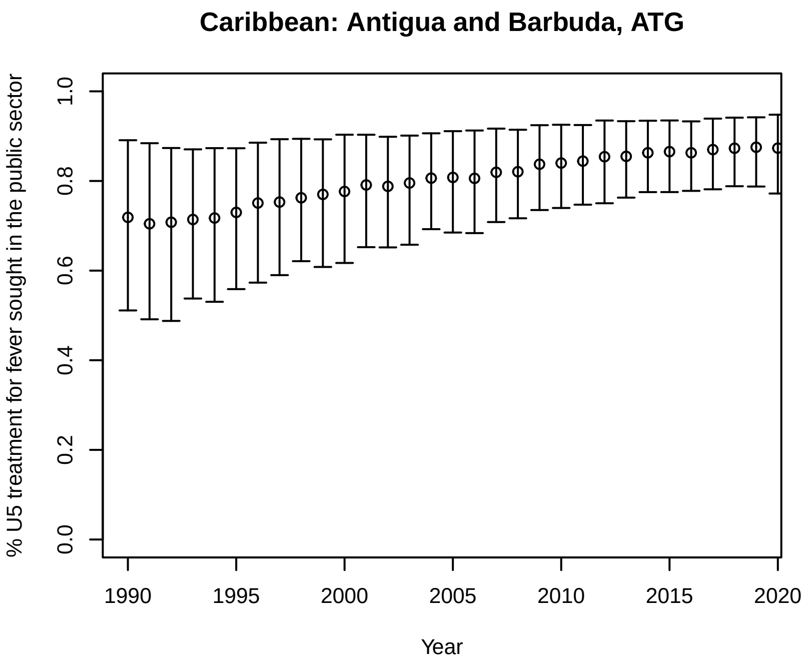
Caribbean: Antigua and Barbuda, ATG
% U5 treatment for fever sought in the public sector
0.0 0.2 0.4 0.6 0.8 1.0
1990 1995 2000 2005 2010 2015 2020
Year

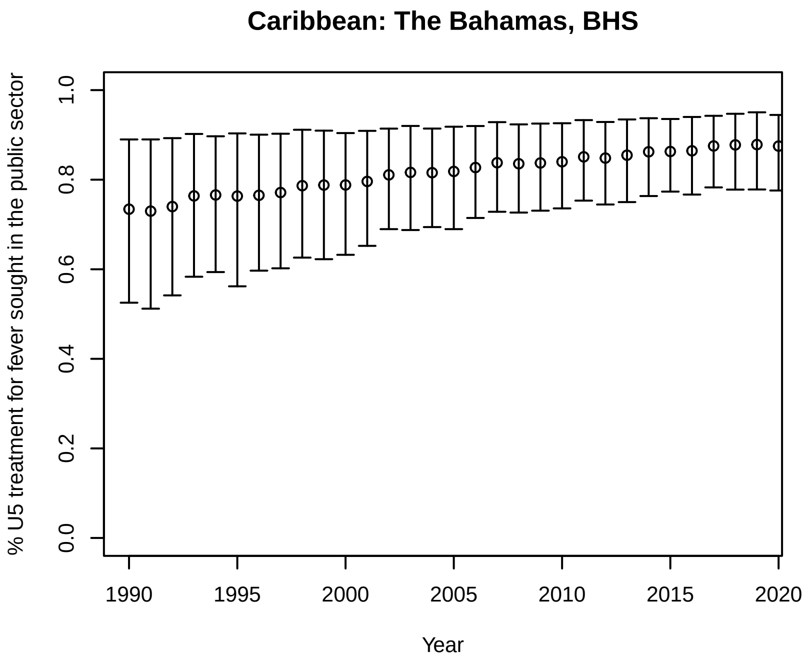
Caribbean: The Bahamas, BHS
% U5 treatment for fever sought in the public sector
0.0 0.2 0.4 0.6 0.8 1.0
1990 1995 2000 2005 2010 2015 2020
Year

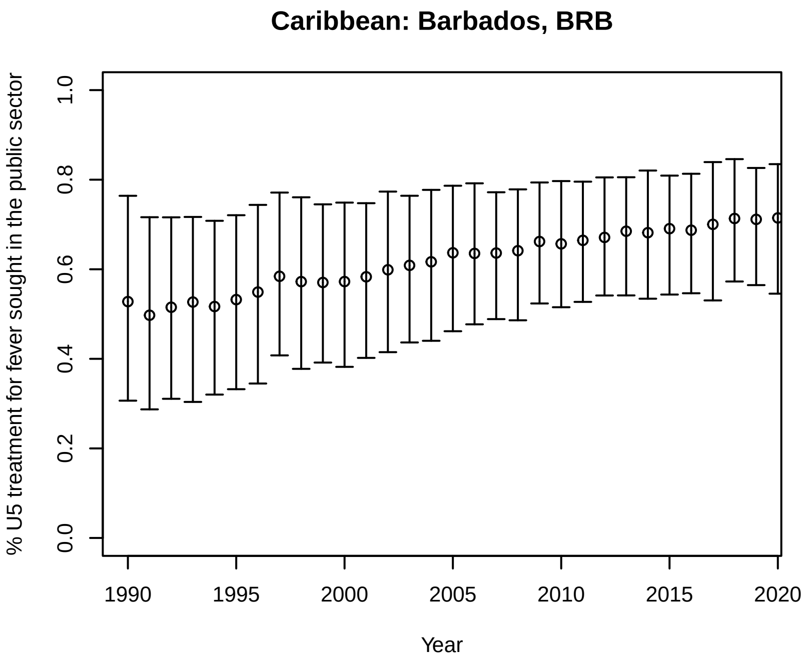
Caribbean: Barbados, BRB
% U5 treatment for fever sought in the public sector
0.0 0.2 0.4 0.6 0.8 1.0
1990 1995 2000 2005 2010 2015 2020
Year

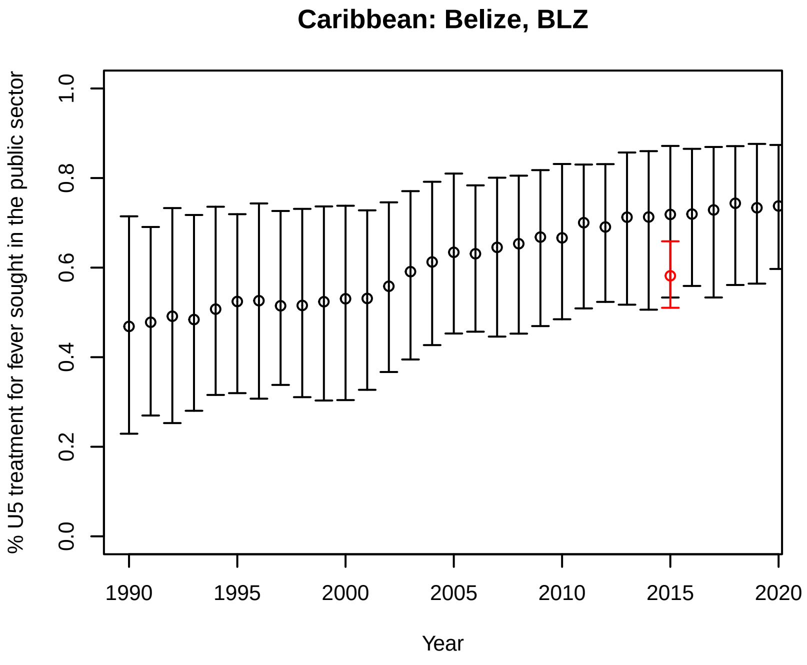
Caribbean: Belize, BLZ
% U5 treatment for fever sought in the public sector
0.0 0.2 0.4 0.6 0.8 1.0
1990 1995 2000 2005 2010 2015 2020
Year

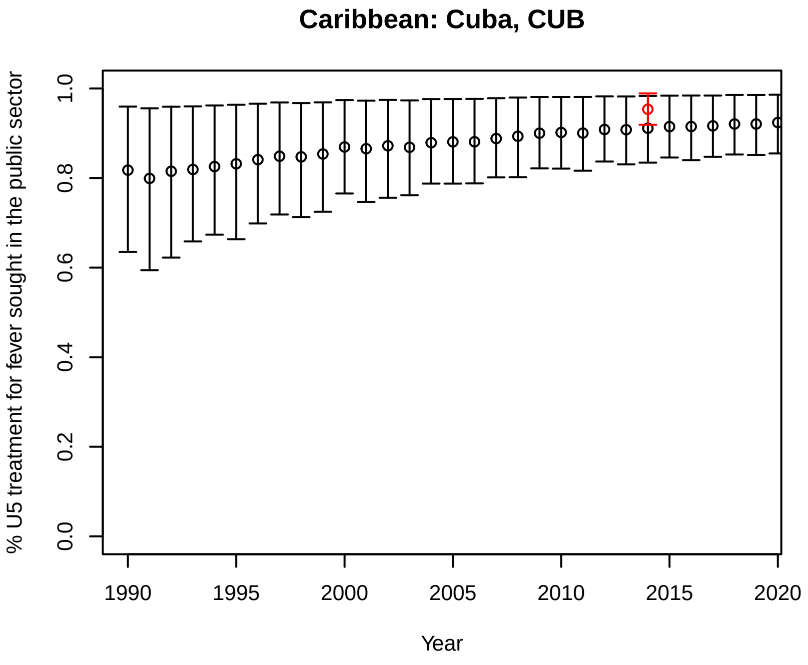
Caribbean: Cuba, CUB
% U5 treatment for fever sought in the public sector
0.0 0.2 0.4 0.6 0.8 1.0
1990 1995 2000 2005 2010 2015 2020
Year

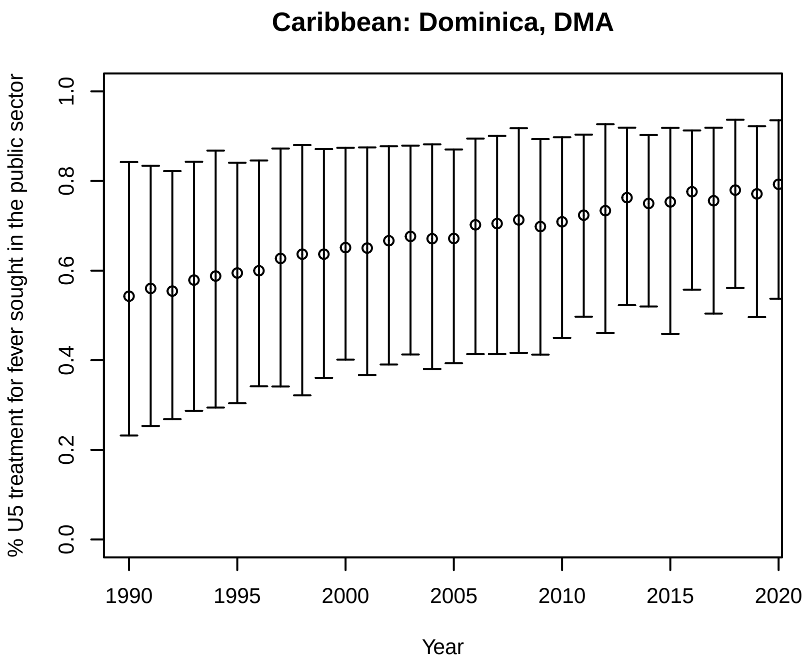
Caribbean: Dominica, DMA
% U5 treatment for fever sought in the public sector
Year
0.0
0.2
0.4
0.6
0.8
1.0
1990
1995
2000
2005
2010
2015
2020

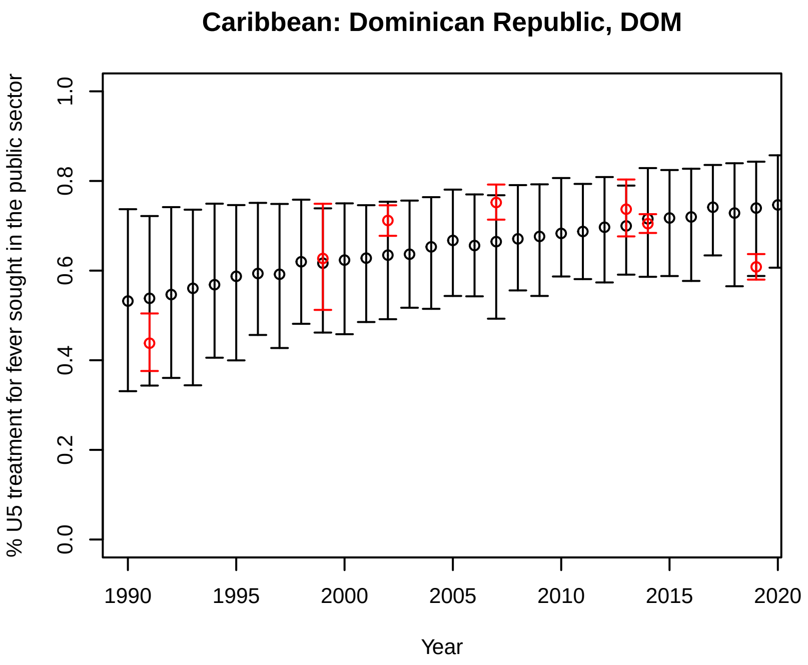
Caribbean: Dominican Republic, DOM
% U5 treatment for fever sought in the public sector
Year
0.0
0.2
0.4
0.6
0.8
1.0
1990
1995
2000
2005
2010
2015
2020

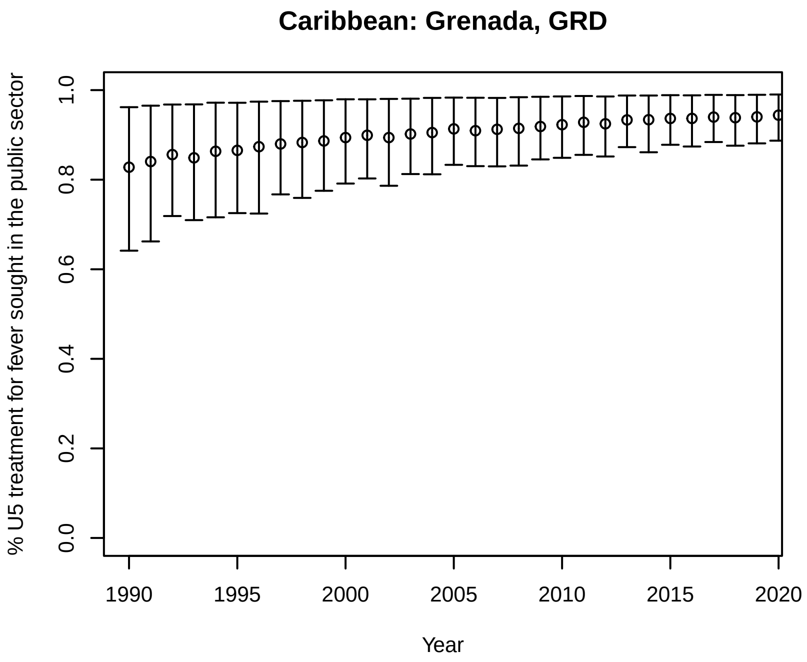
Caribbean: Grenada, GRD
% U5 treatment for fever sought in the public sector
Year
0.0
0.2
0.4
0.6
0.8
1.0
1990
1995
2000
2005
2010
2015
2020

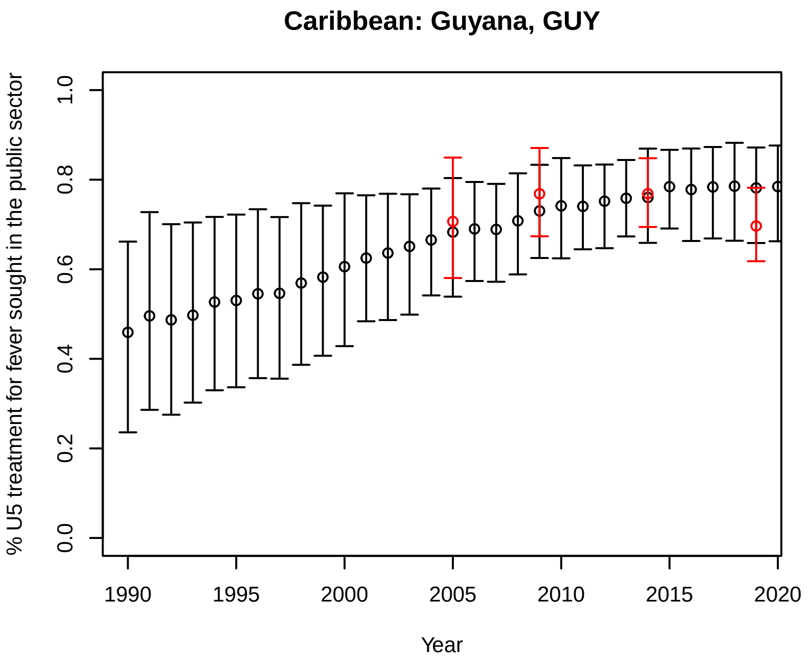
Caribbean: Guyana, GUY
% U5 treatment for fever sought in the public sector
Year
0.0
0.2
0.4
0.6
0.8
1.0
1990
1995
2000
2005
2010
2015
2020

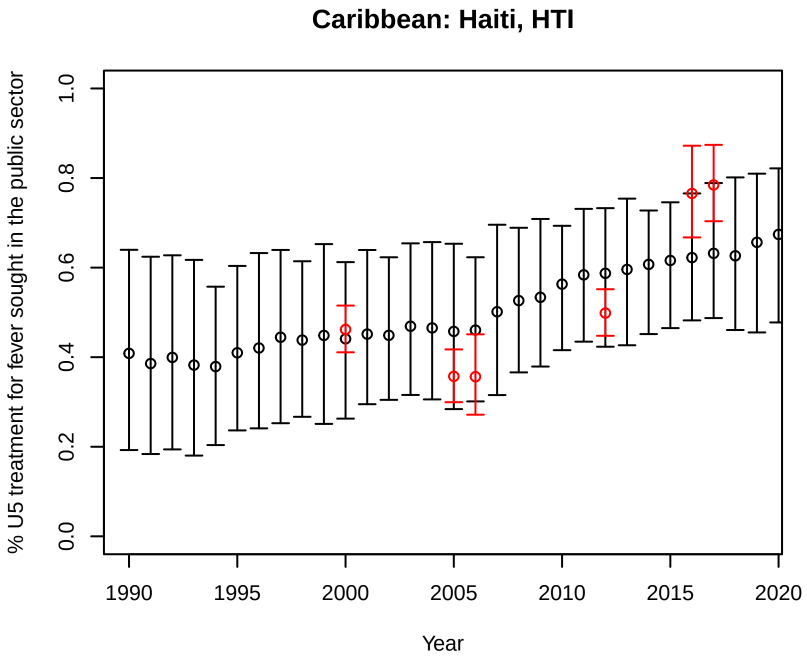
Caribbean: Haiti, HTI
% U5 treatment for fever sought in the public sector
Year
0.0
0.2
0.4
0.6
0.8
1.0
1990
1995
2000
2005
2010
2015
2020

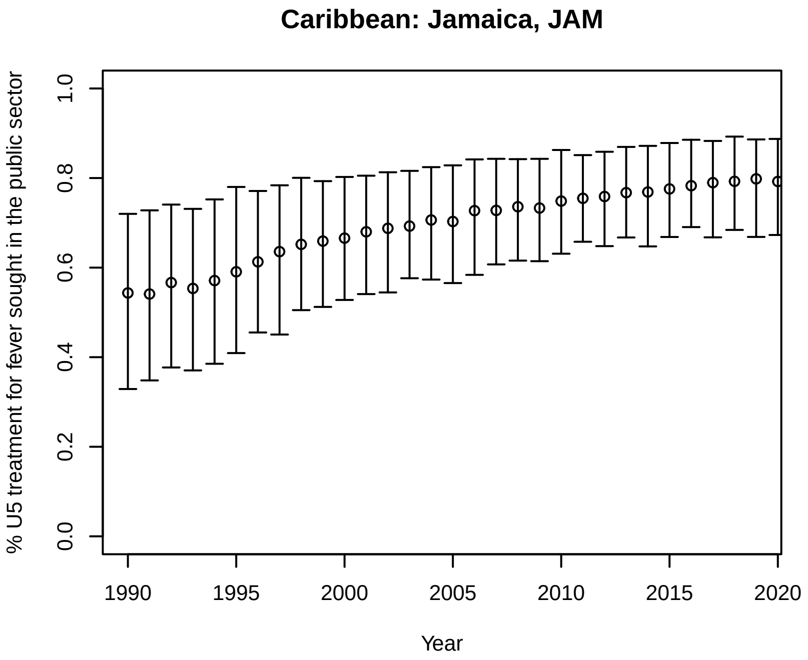
Caribbean: Jamaica, JAM
% U5 treatment for fever sought in the public sector
Year
0.0
0.2
0.4
0.6
0.8
1.0
1990
1995
2000
2005
2010
2015
2020

Caribbean: Saint Lucia, LCA

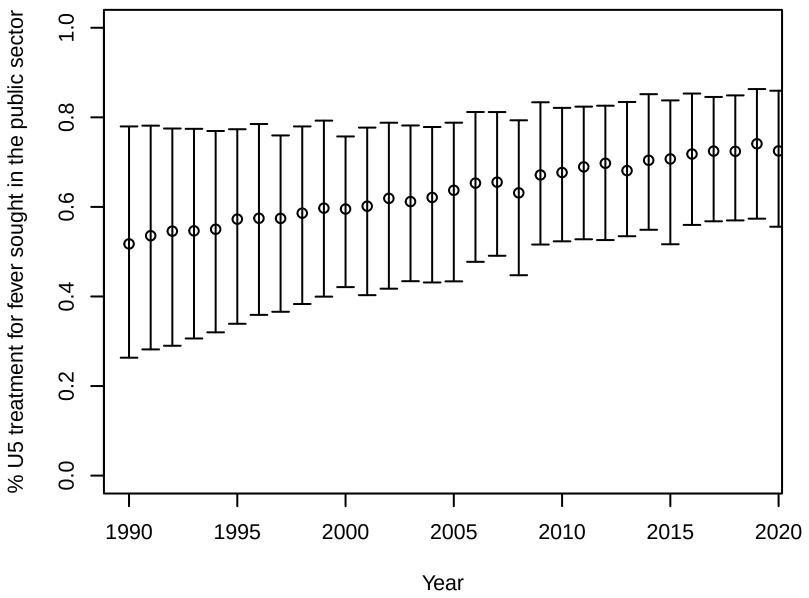

Caribbean: Saint Vincent and the Grenadines, VCT

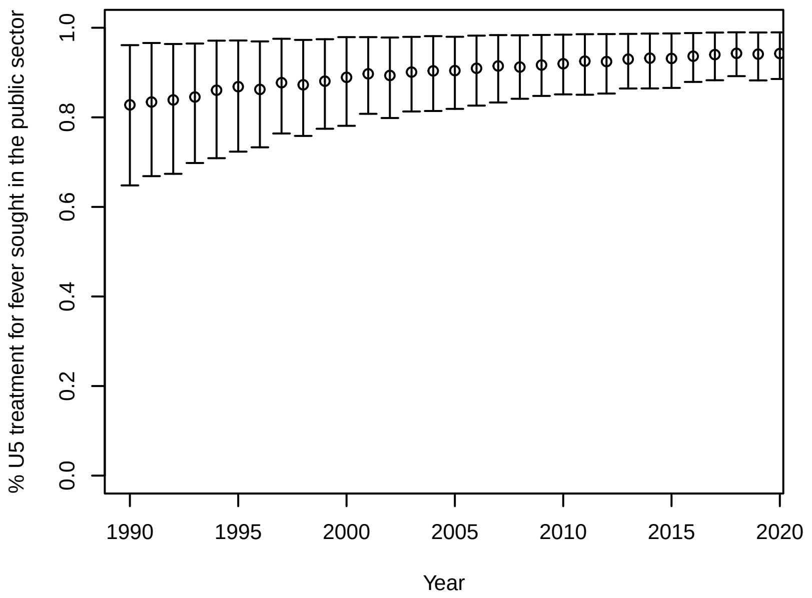

Caribbean: Suriname, SUR

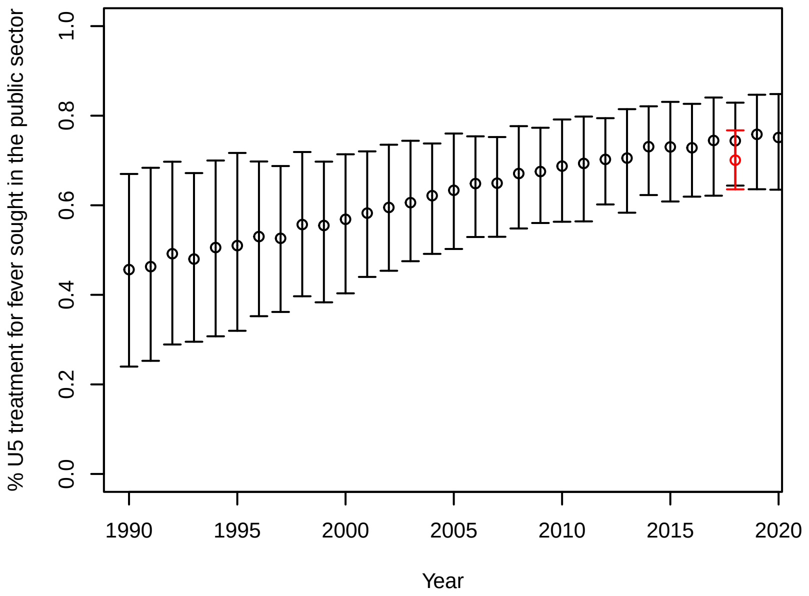

Caribbean: Trinidad and Tobago, TTO

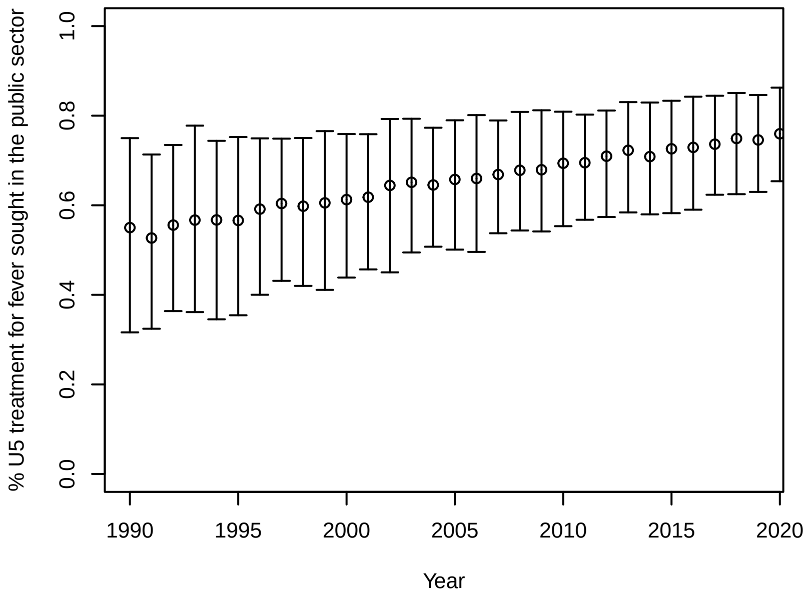

Caribbean: French Guiana, GUF

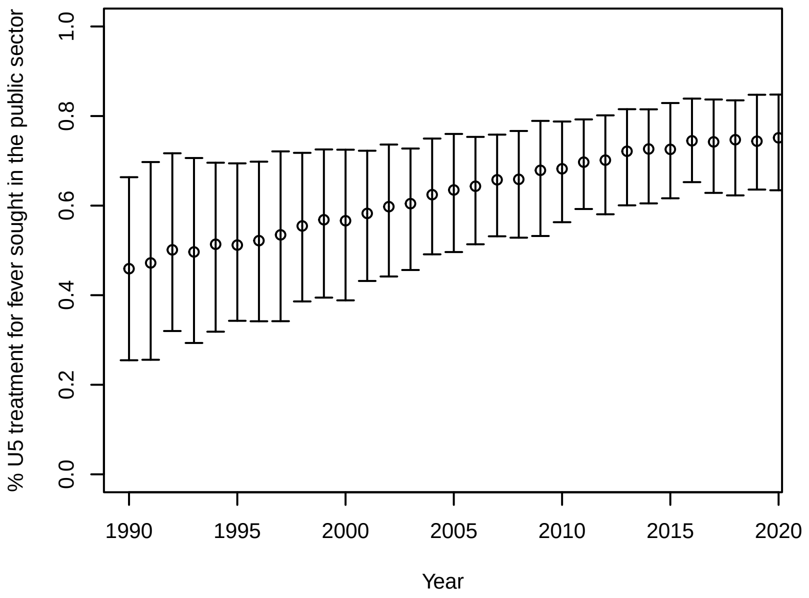

Caribbean: Saint Kitts and Nevis, KNA

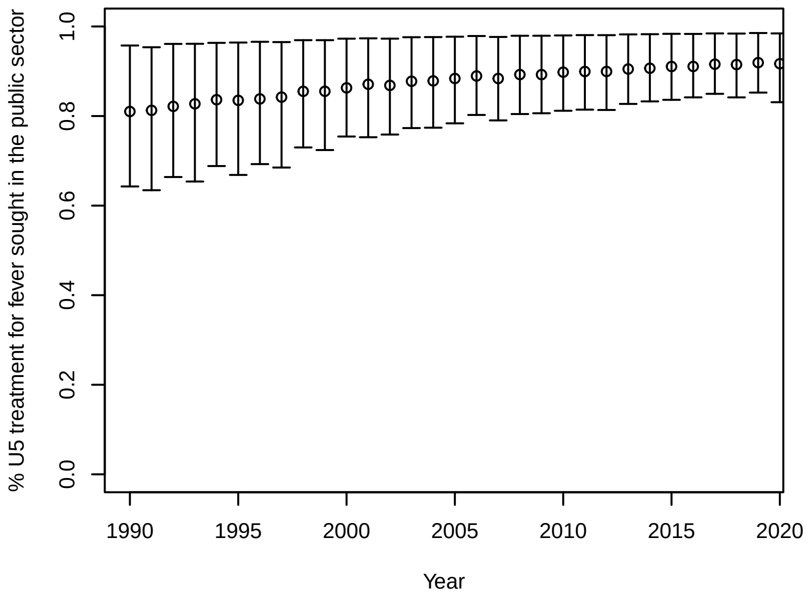

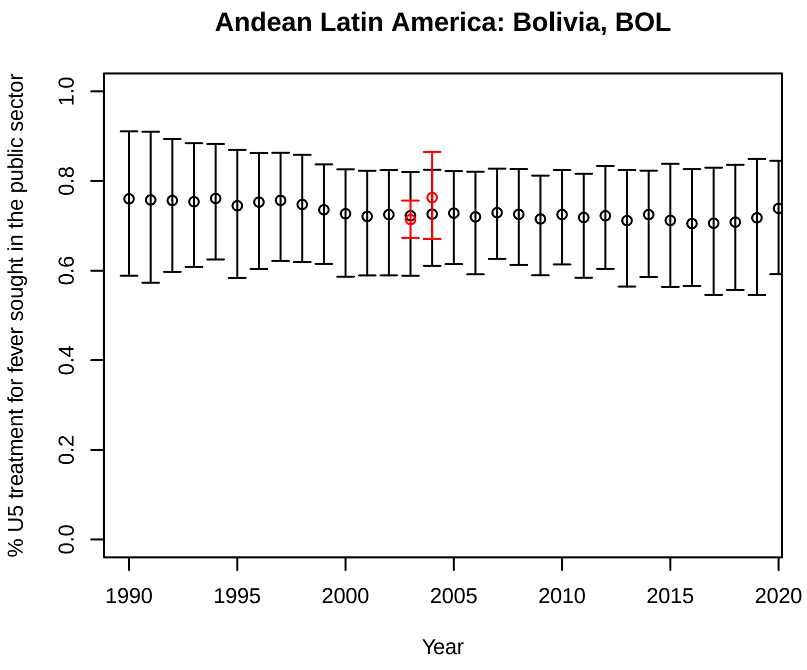
Andean Latin America: Bolivia, BOL
% U5 treatment for fever sought in the public sector
Year
0.0 0.2 0.4 0.6 0.8 1.0
1990 1995 2000 2005 2010 2015 2020

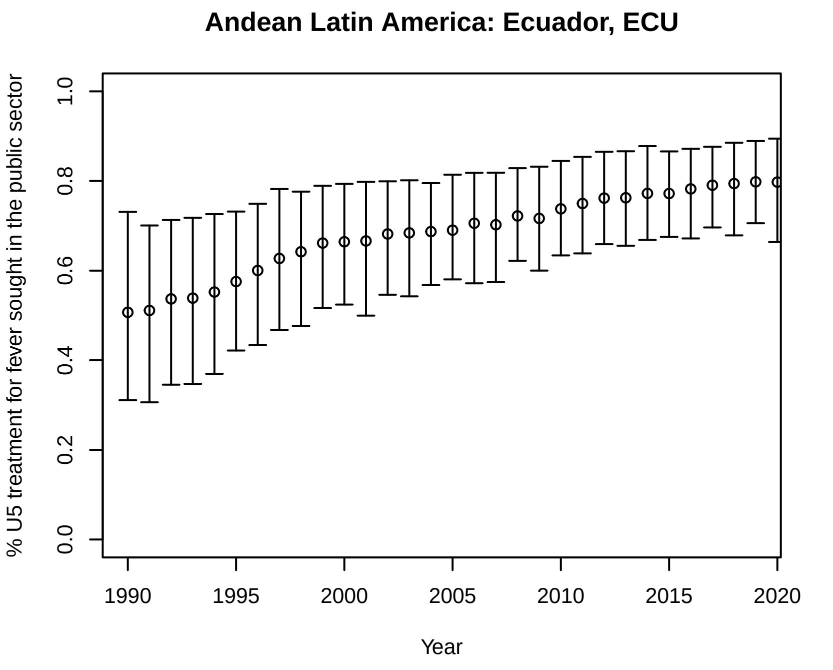
Andean Latin America: Ecuador, ECU
% U5 treatment for fever sought in the public sector
Year
0.0 0.2 0.4 0.6 0.8 1.0
1990 1995 2000 2005 2010 2015 2020

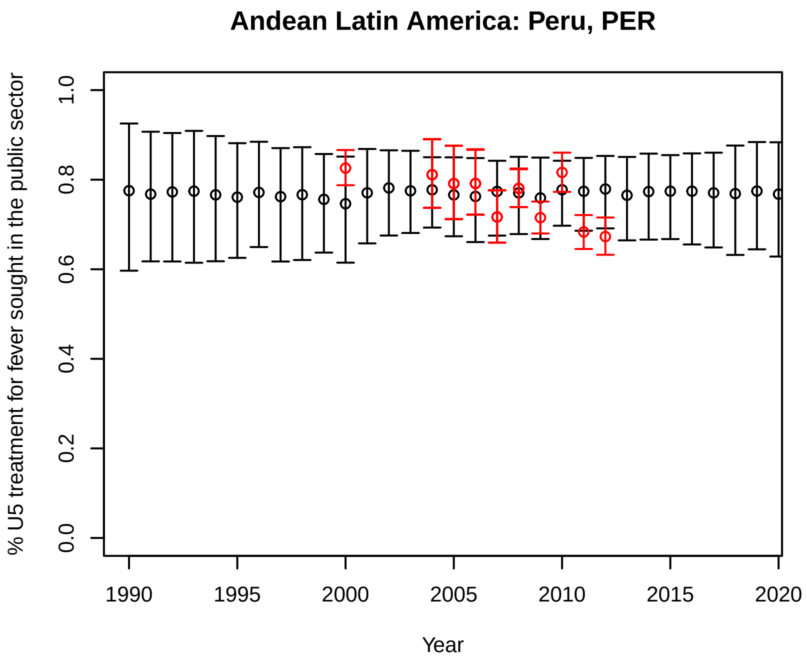
Andean Latin America: Peru, PER
% U5 treatment for fever sought in the public sector
Year
0.0 0.2 0.4 0.6 0.8 1.0
1990 1995 2000 2005 2010 2015 2020

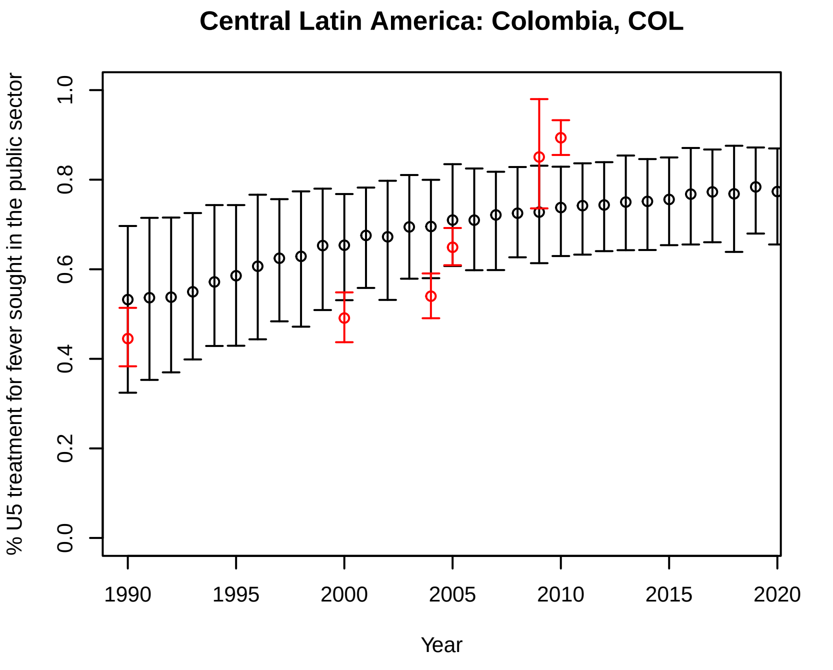
Central Latin America: Colombia, COL
% U5 treatment for fever sought in the public sector
Year
0.0 0.2 0.4 0.6 0.8 1.0
1990 1995 2000 2005 2010 2015 2020

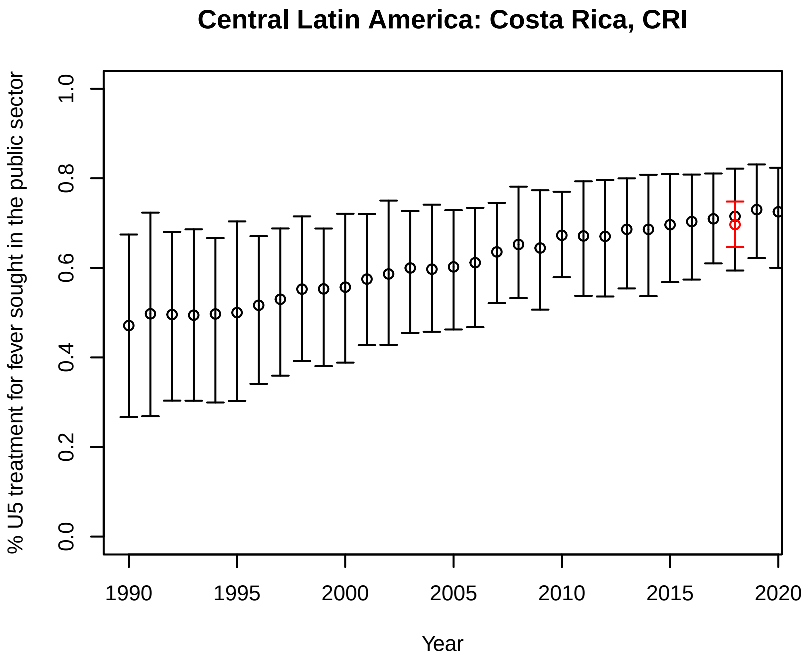
Central Latin America: Costa Rica, CRI
% U5 treatment for fever sought in the public sector
Year
0.0 0.2 0.4 0.6 0.8 1.0
1990 1995 2000 2005 2010 2015 2020

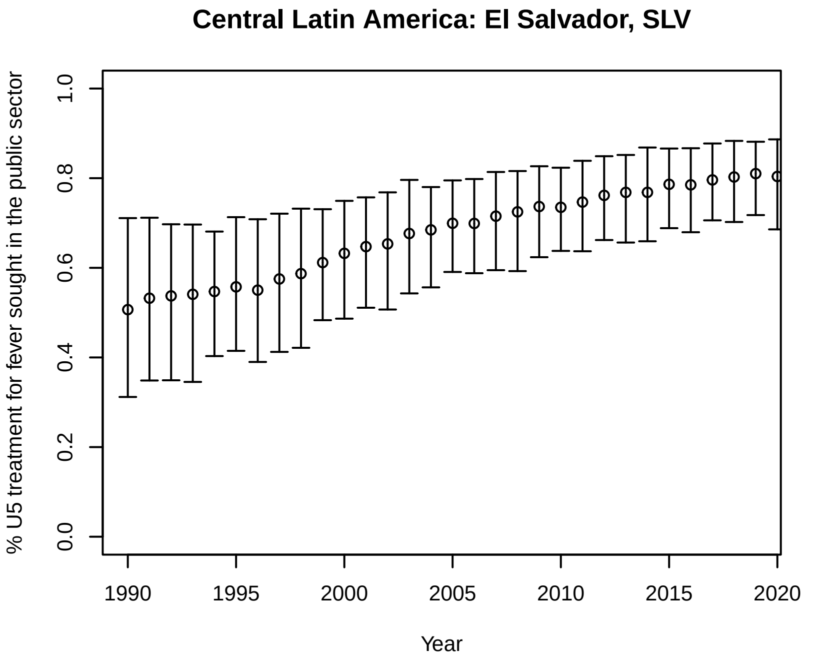
Central Latin America: El Salvador, SLV
% U5 treatment for fever sought in the public sector
Year
0.0 0.2 0.4 0.6 0.8 1.0
1990 1995 2000 2005 2010 2015 2020

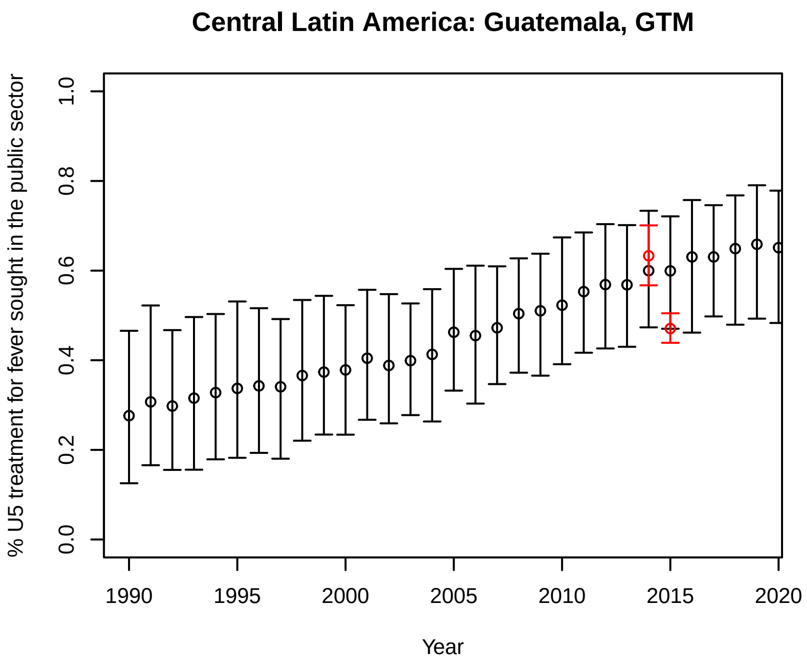
Central Latin America: Guatemala, GTM
% U5 treatment for fever sought in the public sector
0.0
0.2
0.4
0.6
0.8
1.0
1990
1995
2000
2005
2010
2015
2020
Year

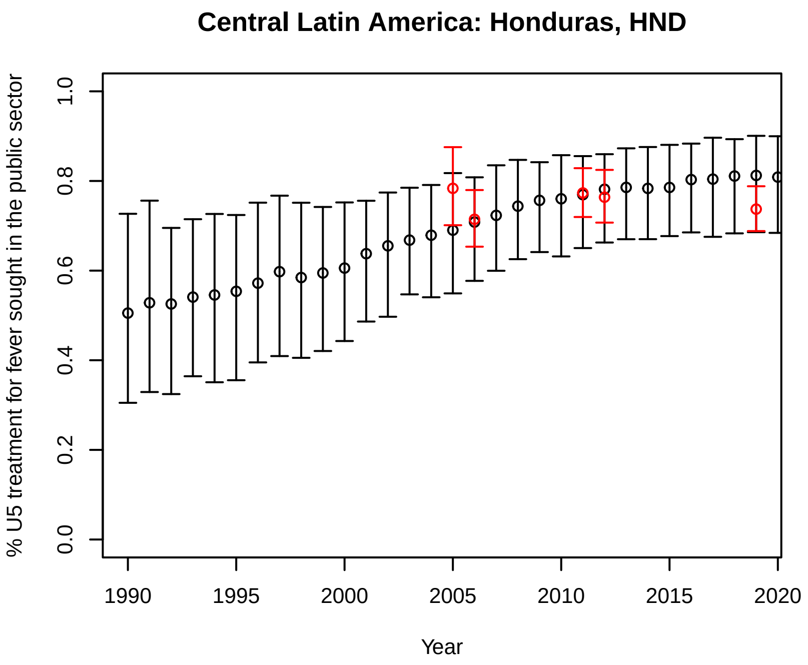
Central Latin America: Honduras, HND
% U5 treatment for fever sought in the public sector
0.0
0.2
0.4
0.6
0.8
1.0
1990
1995
2000
2005
2010
2015
2020
Year

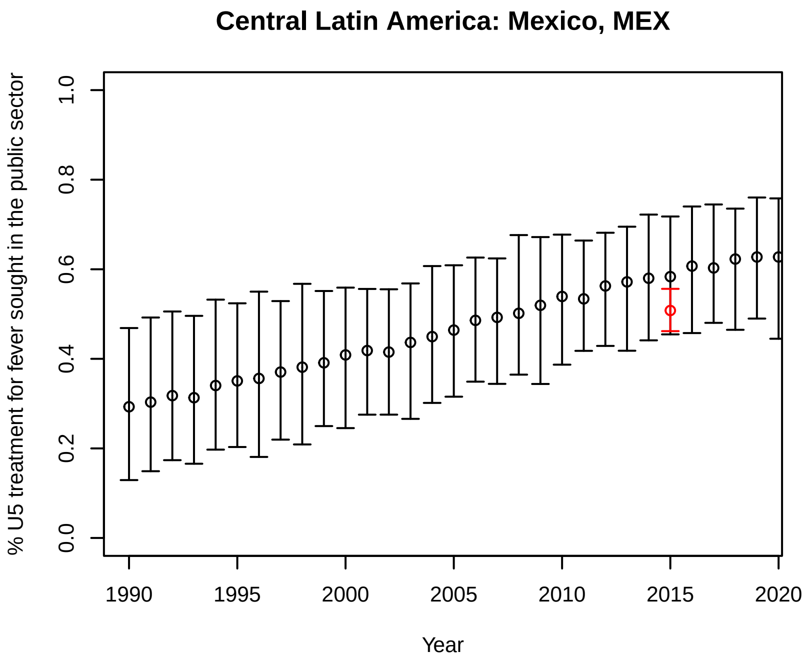
Central Latin America: Mexico, MEX
% U5 treatment for fever sought in the public sector
0.0
0.2
0.4
0.6
0.8
1.0
1990
1995
2000
2005
2010
2015
2020
Year

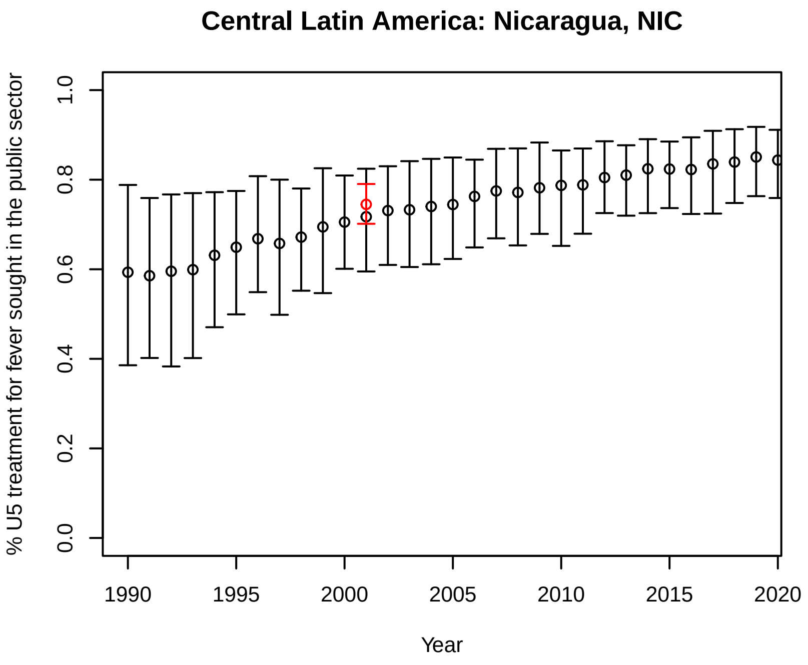
Central Latin America: Nicaragua, NIC
% U5 treatment for fever sought in the public sector
0.0
0.2
0.4
0.6
0.8
1.0
1990
1995
2000
2005
2010
2015
2020
Year

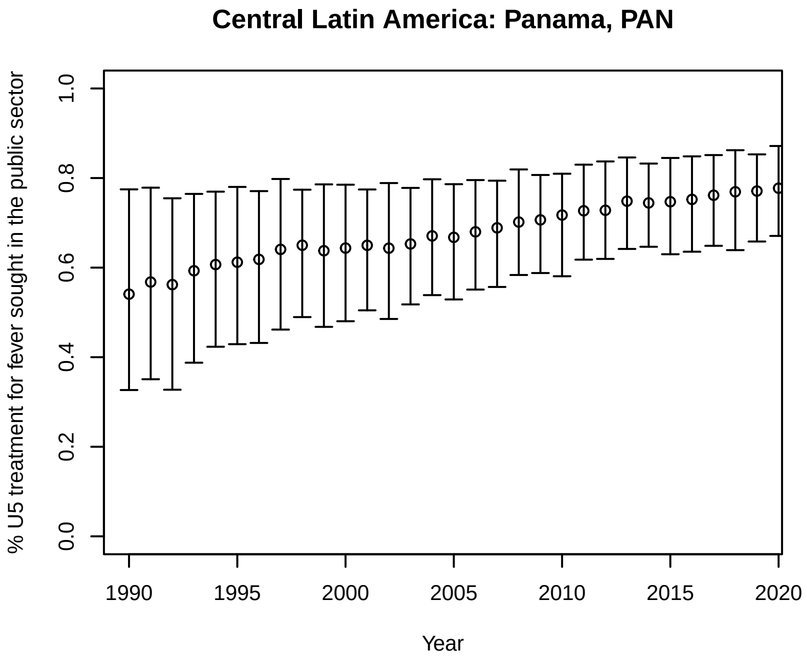
Central Latin America: Panama, PAN
% U5 treatment for fever sought in the public sector
0.0
0.2
0.4
0.6
0.8
1.0
1990
1995
2000
2005
2010
2015
2020
Year

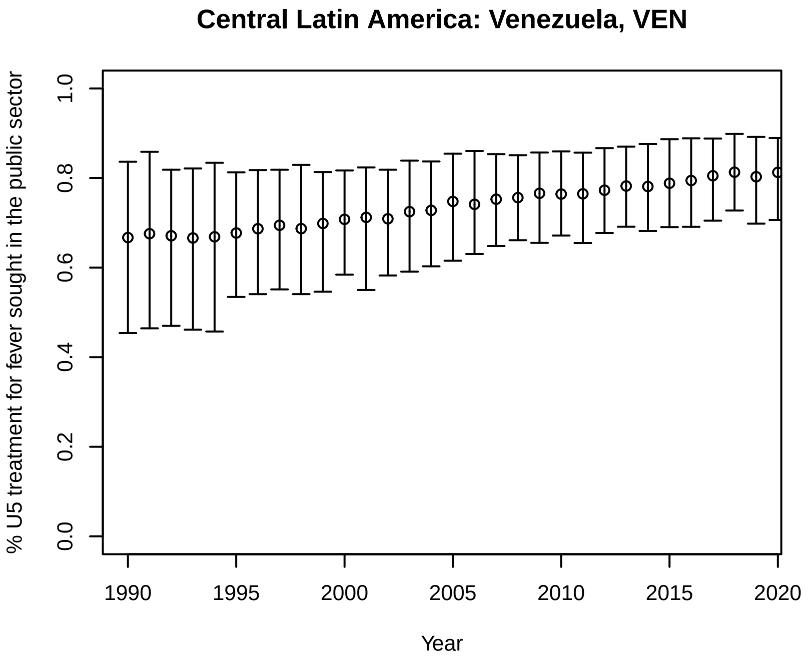
Central Latin America: Venezuela, VEN
% U5 treatment for fever sought in the public sector
0.0
0.2
0.4
0.6
0.8
1.0
1990
1995
2000
2005
2010
2015
2020
Year

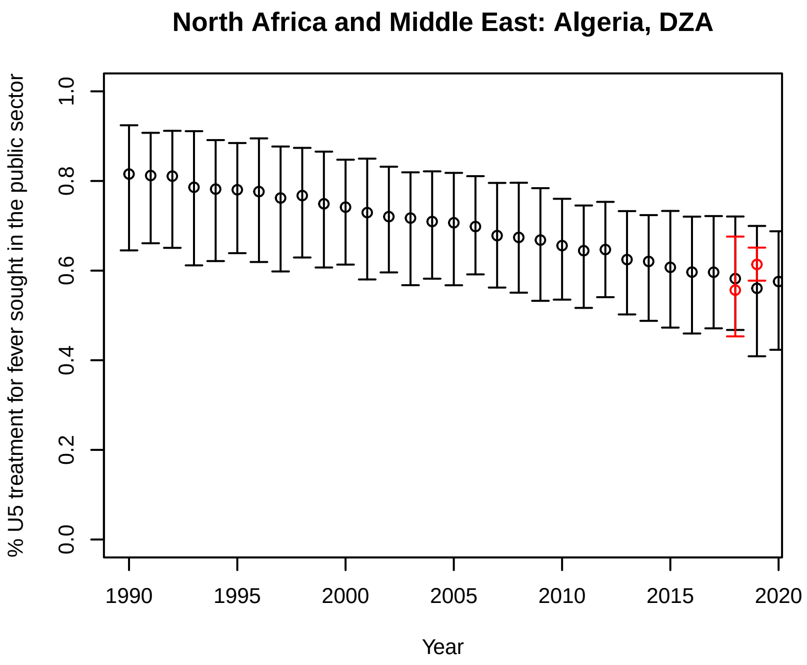
North Africa and Middle East: Algeria, DZA
% U5 treatment for fever sought in the public sector
Year

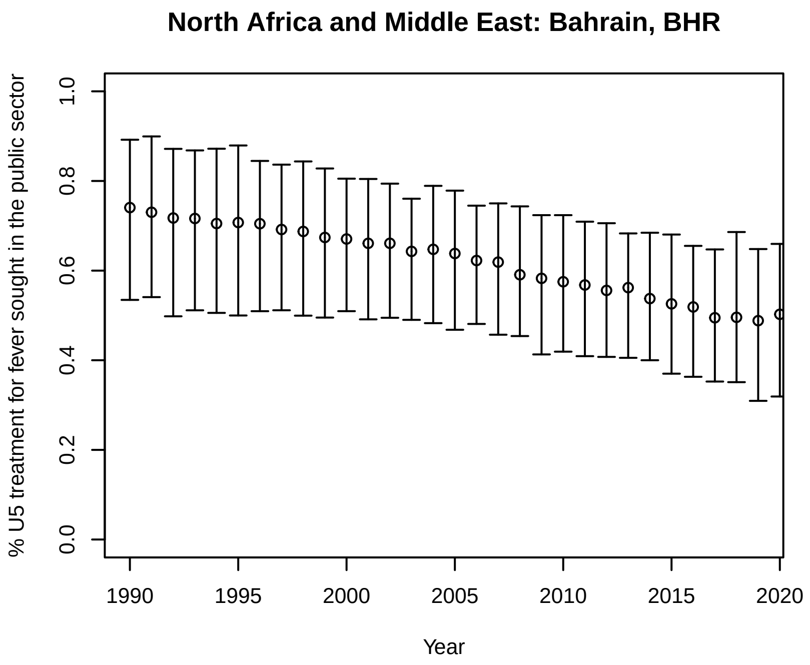
North Africa and Middle East: Bahrain, BHR
% U5 treatment for fever sought in the public sector
Year

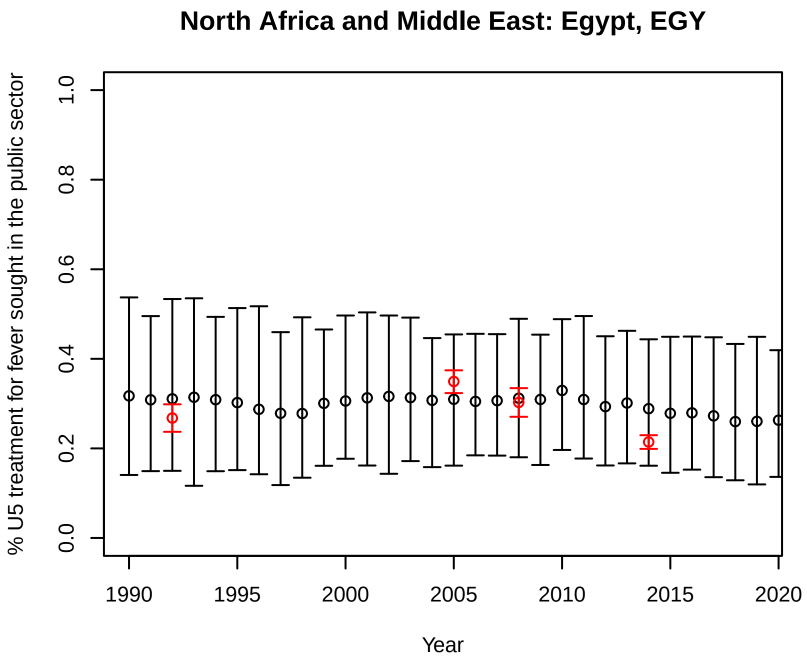
North Africa and Middle East: Egypt, EGY
% U5 treatment for fever sought in the public sector
Year

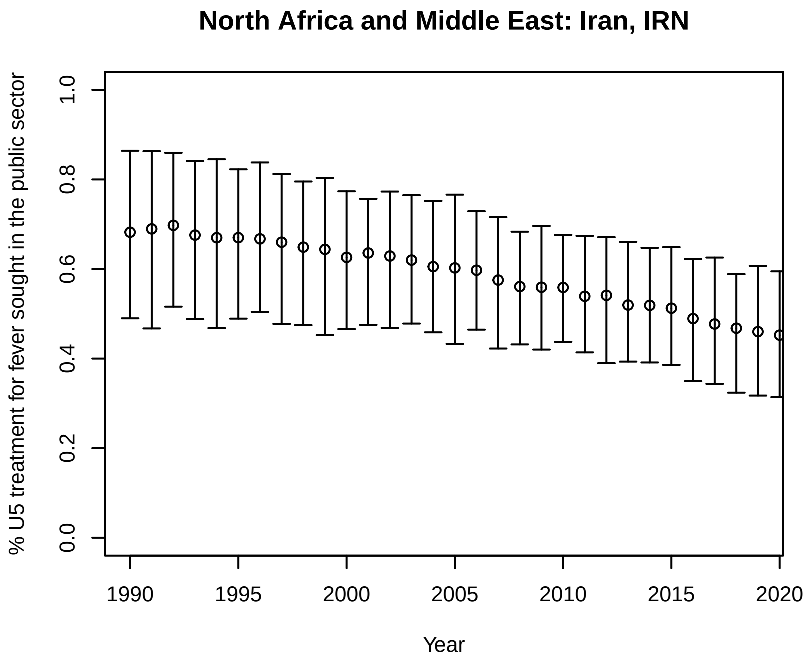
North Africa and Middle East: Iran, IRN
% U5 treatment for fever sought in the public sector
Year

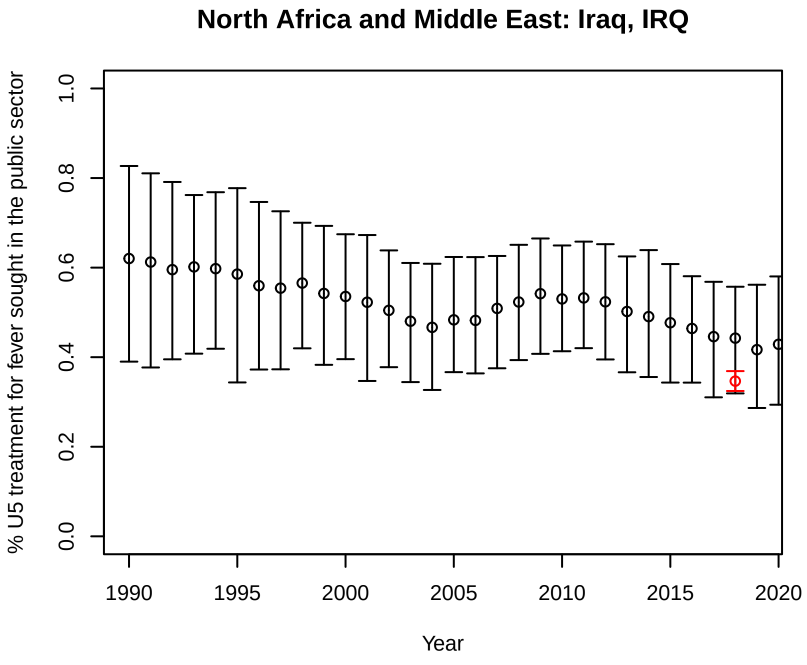
North Africa and Middle East: Iraq, IRQ
% U5 treatment for fever sought in the public sector
Year

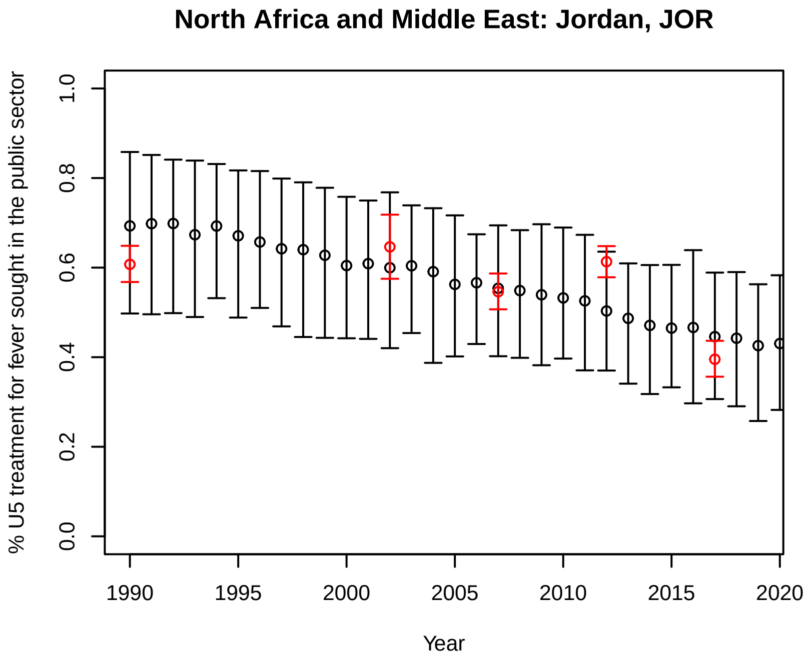
North Africa and Middle East: Jordan, JOR
% U5 treatment for fever sought in the public sector
Year

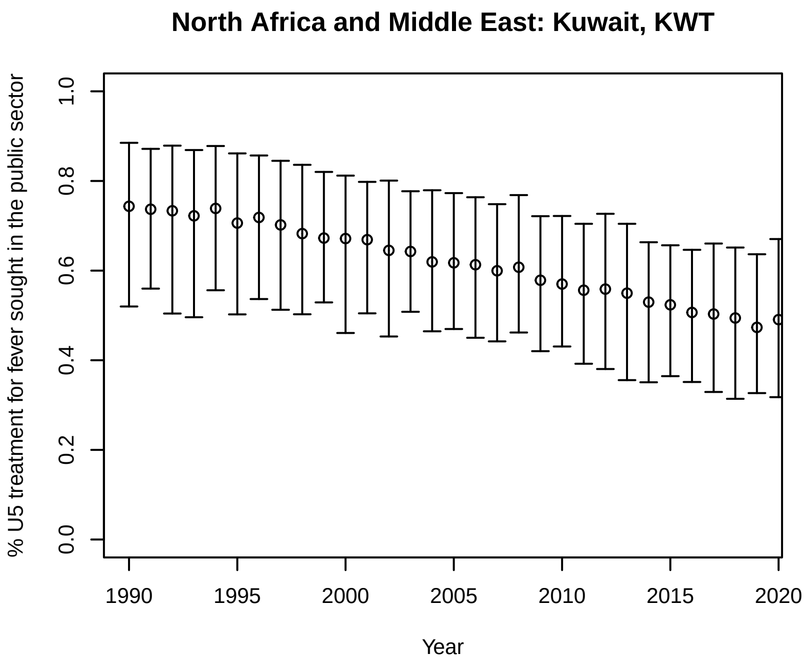

North Africa and Middle East: Kuwait, KWT
% U5 treatment for fever sought in the public sector
Year

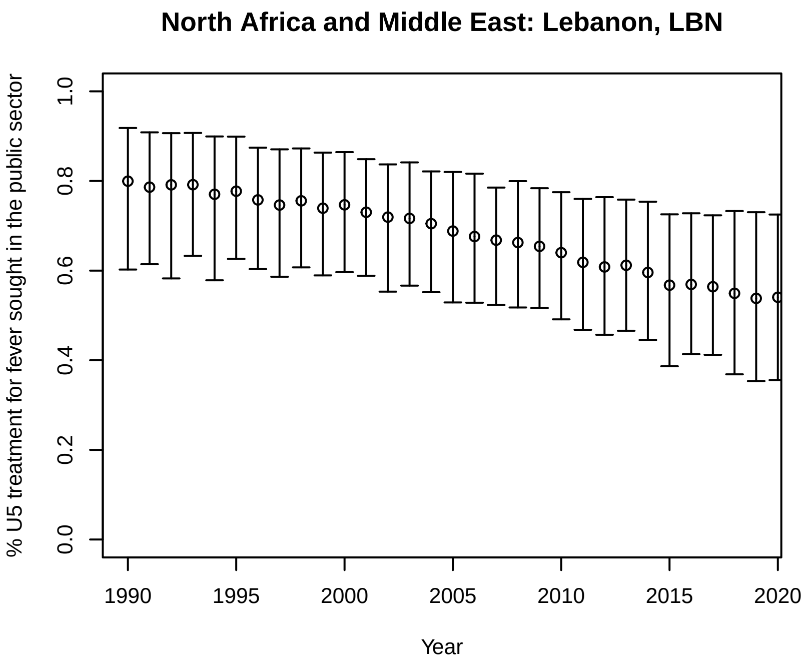

North Africa and Middle East: Lebanon, LBN
% U5 treatment for fever sought in the public sector
Year

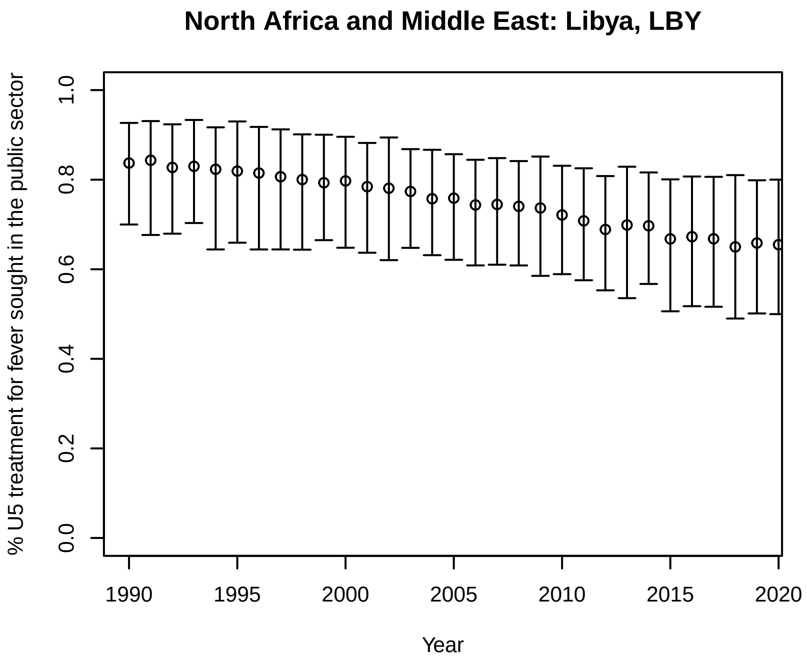

North Africa and Middle East: Libya, LBY
% U5 treatment for fever sought in the public sector
Year

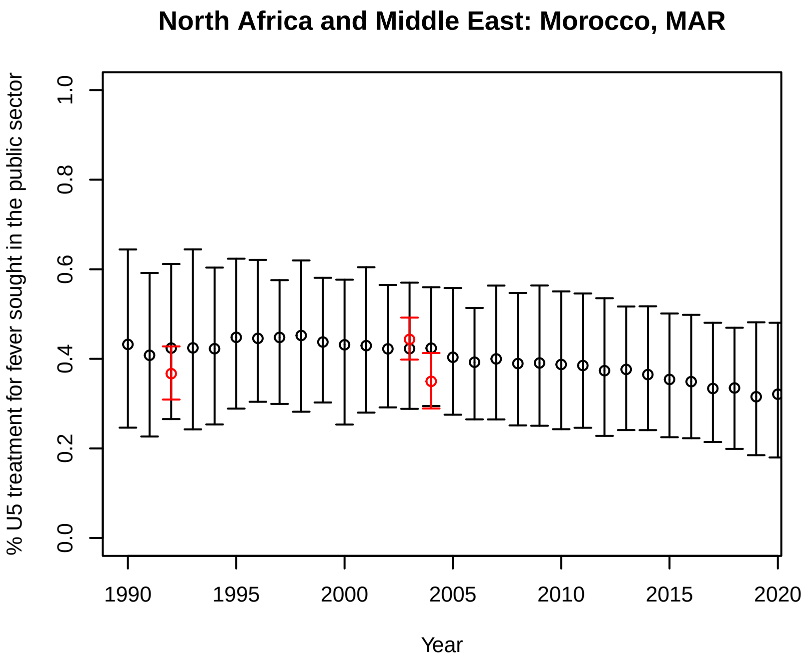

North Africa and Middle East: Morocco, MAR
% U5 treatment for fever sought in the public sector
Year

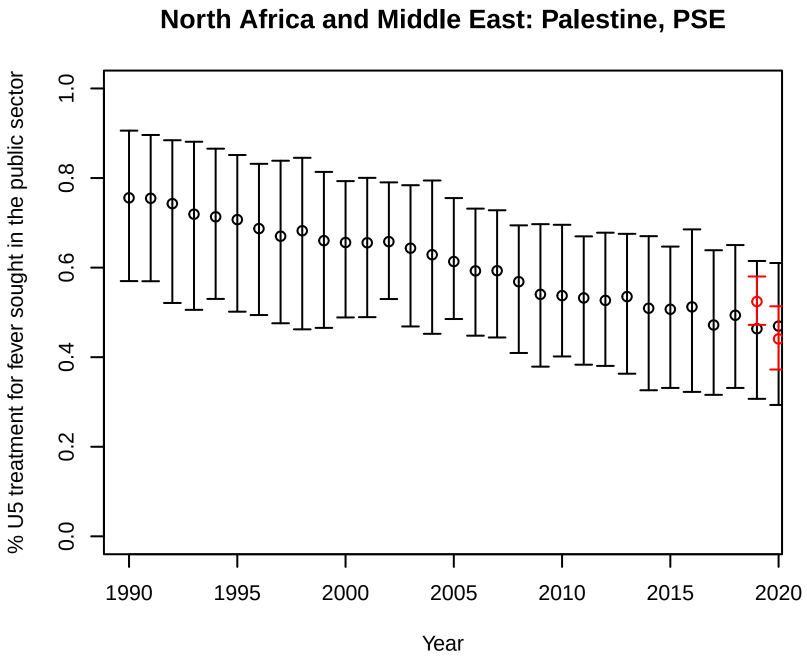

North Africa and Middle East: Palestine, PSE
% U5 treatment for fever sought in the public sector
Year

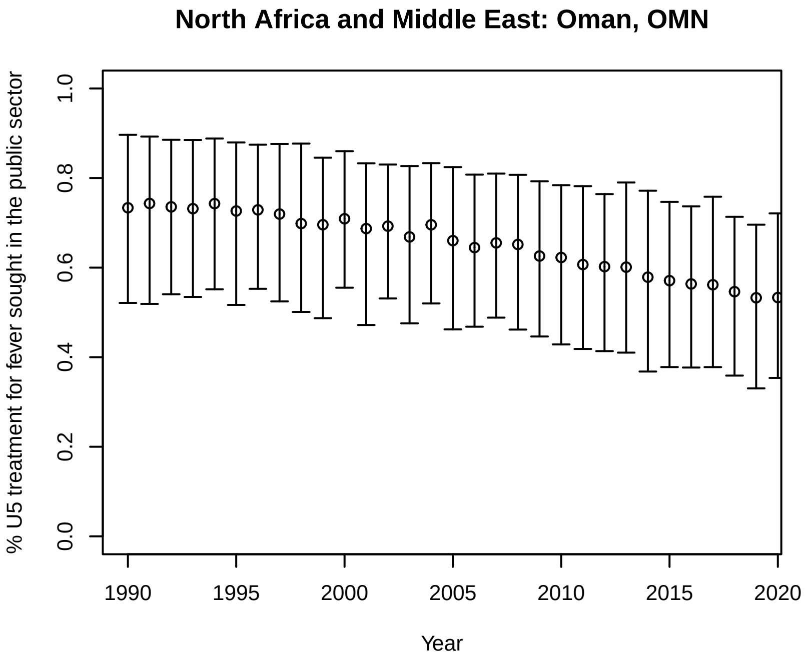

North Africa and Middle East: Oman, OMN
% U5 treatment for fever sought in the public sector
Year

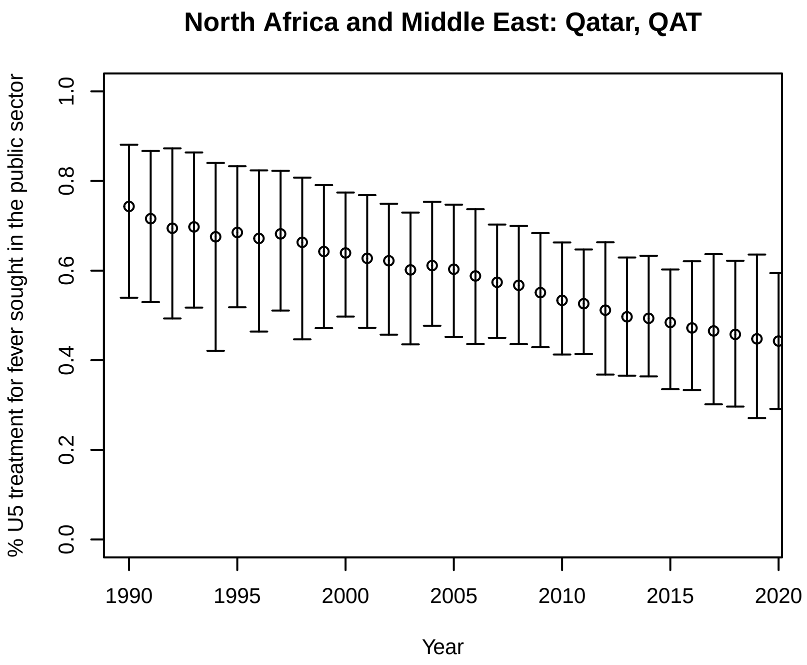
North Africa and Middle East: Qatar, QAT
% U5 treatment for fever sought in the public sector
Year

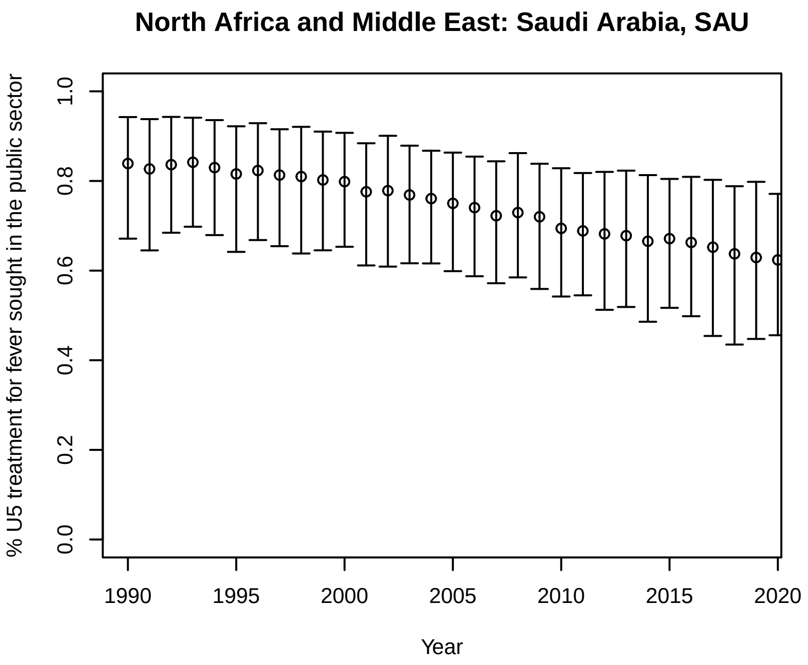
North Africa and Middle East: Saudi Arabia, SAU
% U5 treatment for fever sought in the public sector
Year

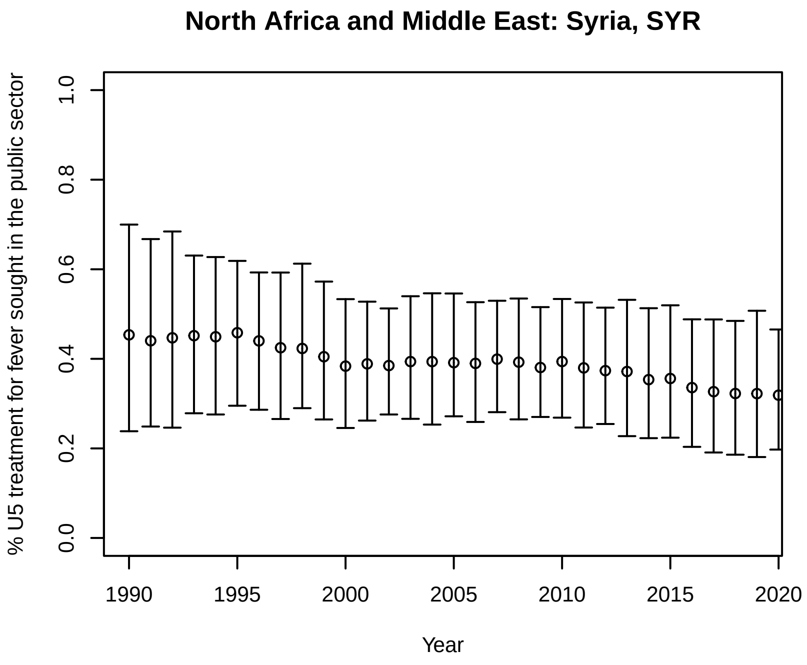
North Africa and Middle East: Syria, SYR
% U5 treatment for fever sought in the public sector
Year

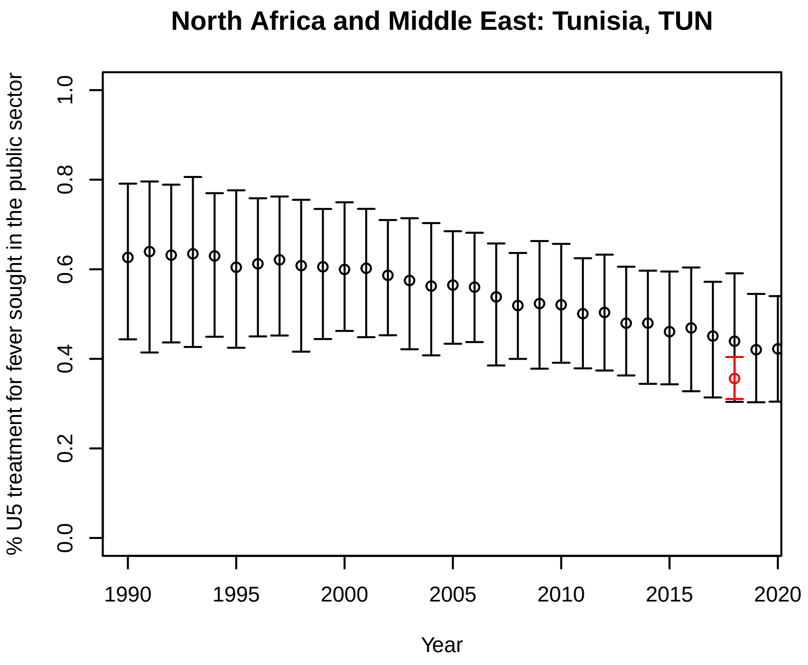
North Africa and Middle East: Tunisia, TUN
% U5 treatment for fever sought in the public sector
Year

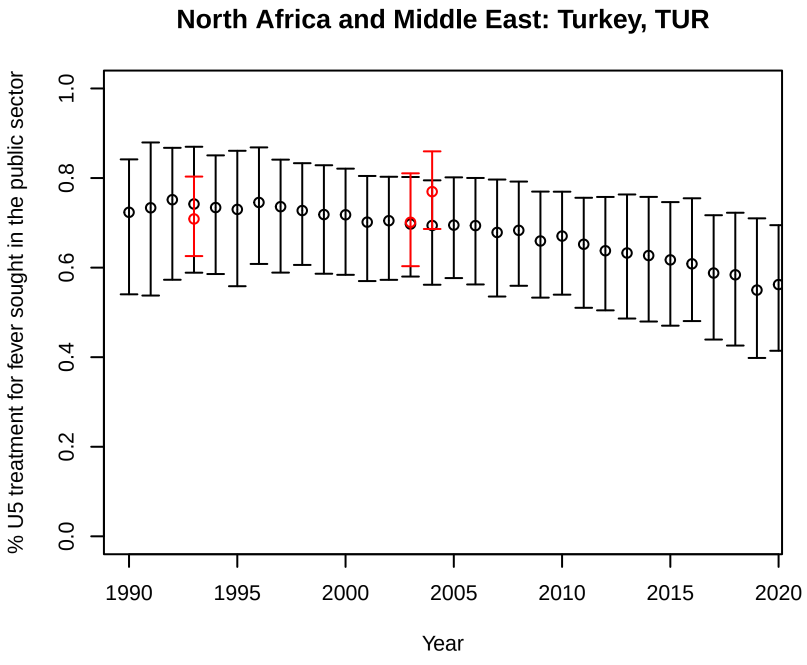
North Africa and Middle East: Turkey, TUR
% U5 treatment for fever sought in the public sector
Year

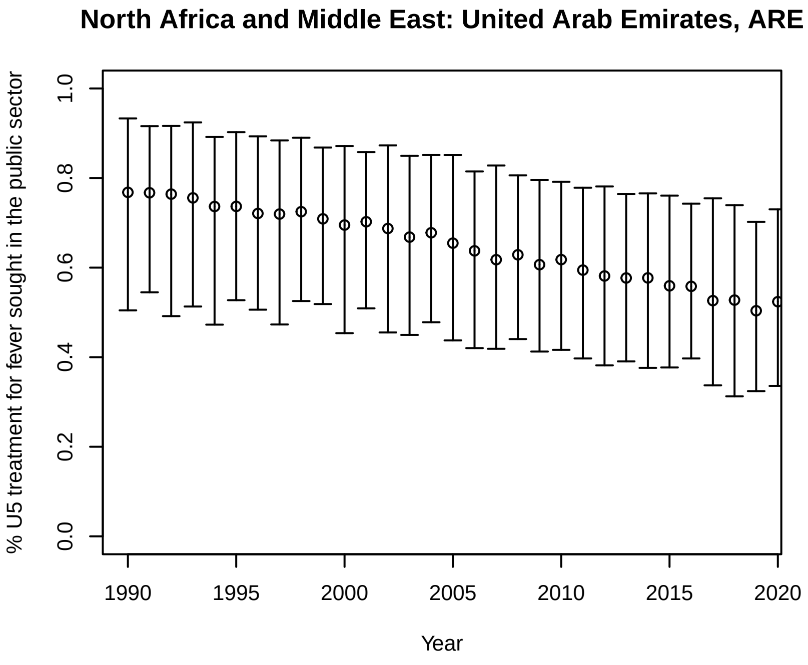
North Africa and Middle East: United Arab Emirates, ARE
% U5 treatment for fever sought in the public sector
Year

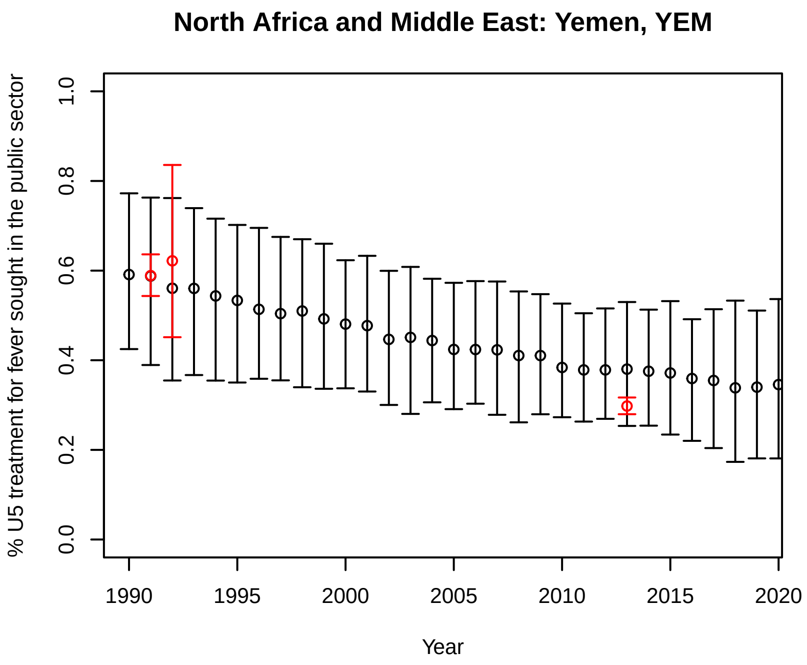
North Africa and Middle East: Yemen, YEM
% U5 treatment for fever sought in the public sector
0.0
0.2
0.4
0.6
0.8
1.0
1990
1995
2000
2005
2010
2015
2020
Year

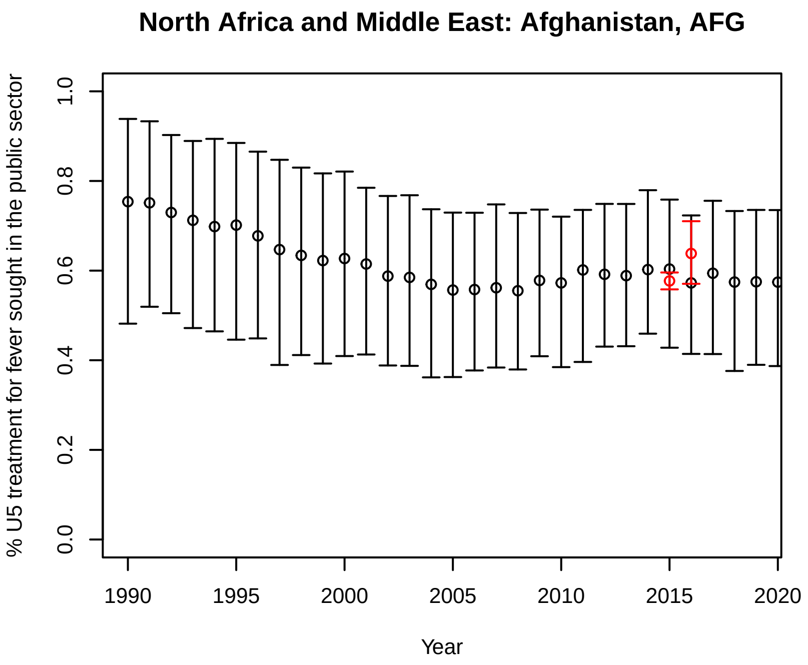
North Africa and Middle East: Afghanistan, AFG
% U5 treatment for fever sought in the public sector
0.0
0.2
0.4
0.6
0.8
1.0
1990
1995
2000
2005
2010
2015
2020
Year

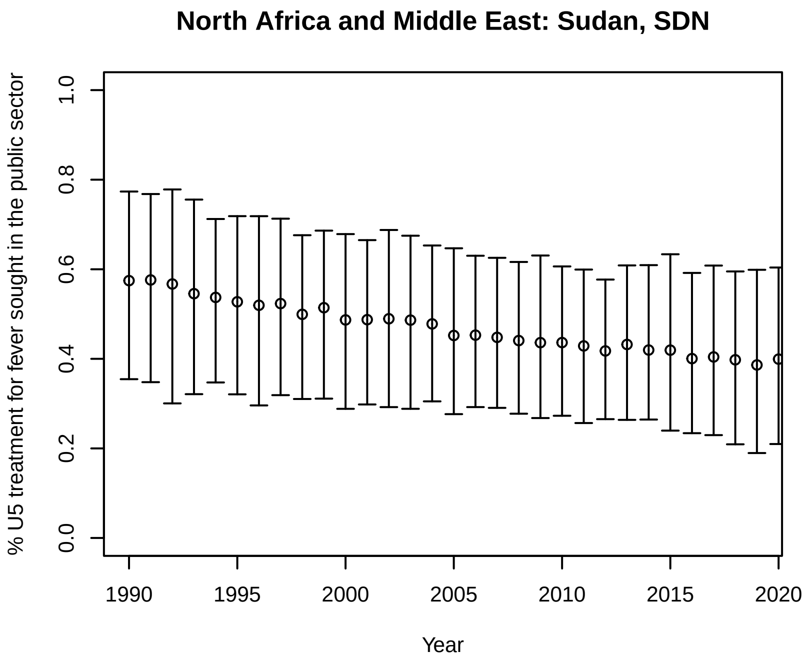
North Africa and Middle East: Sudan, SDN
% U5 treatment for fever sought in the public sector
0.0
0.2
0.4
0.6
0.8
1.0
1990
1995
2000
2005
2010
2015
2020
Year

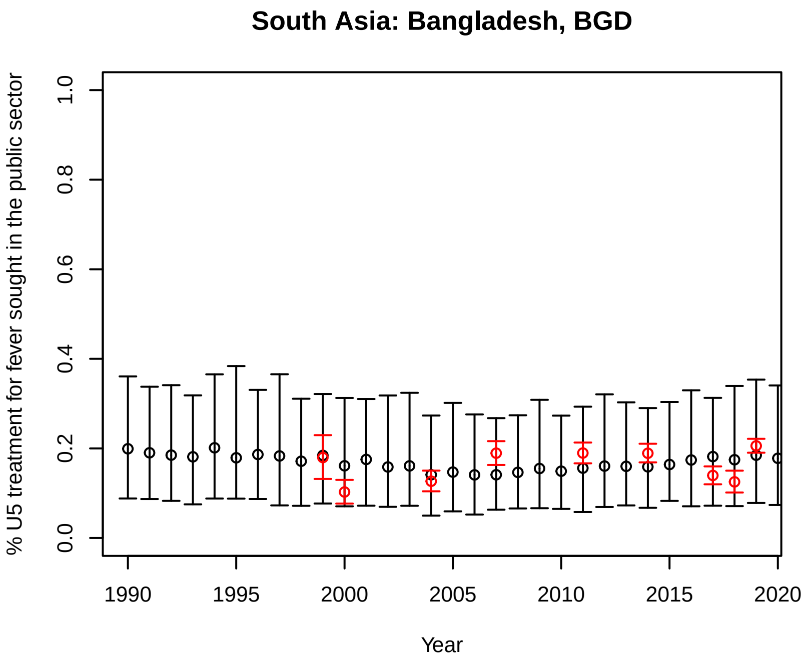
South Asia: Bangladesh, BGD
% U5 treatment for fever sought in the public sector
0.0
0.2
0.4
0.6
0.8
1.0
1990
1995
2000
2005
2010
2015
2020
Year

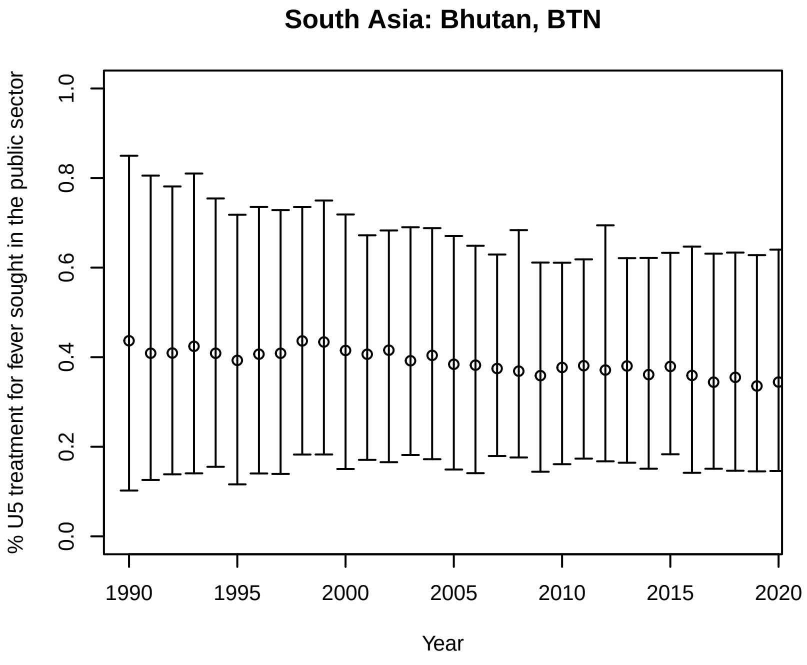
South Asia: Bhutan, BTN
% U5 treatment for fever sought in the public sector
0.0
0.2
0.4
0.6
0.8
1.0
1990
1995
2000
2005
2010
2015
2020
Year

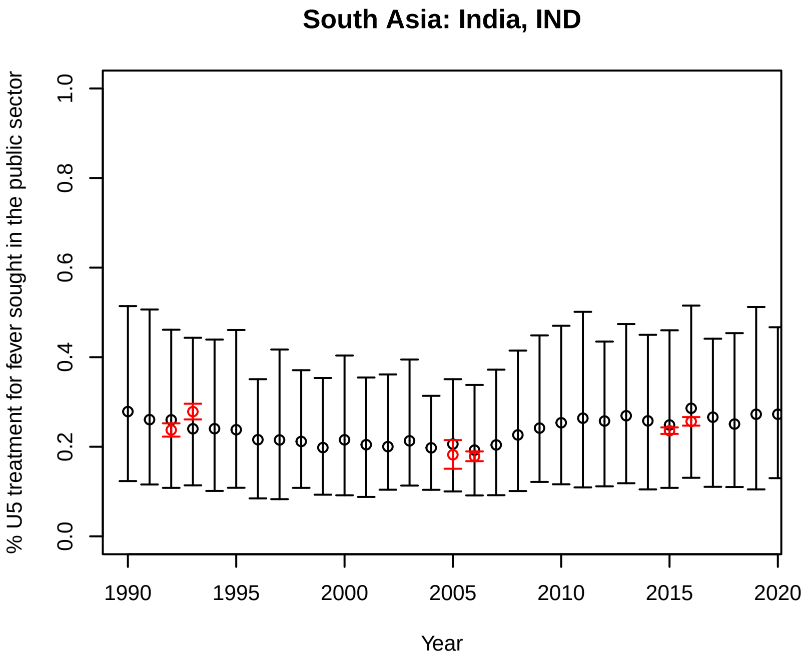
South Asia: India, IND
% U5 treatment for fever sought in the public sector
0.0
0.2
0.4
0.6
0.8
1.0
1990
1995
2000
2005
2010
2015
2020
Year

South Asia: Nepal, NPL

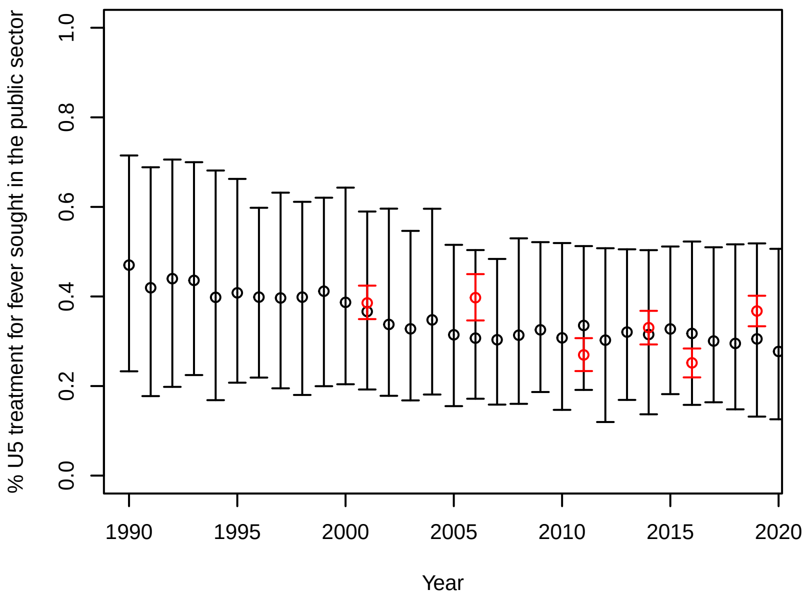

South Asia: Pakistan, PAK

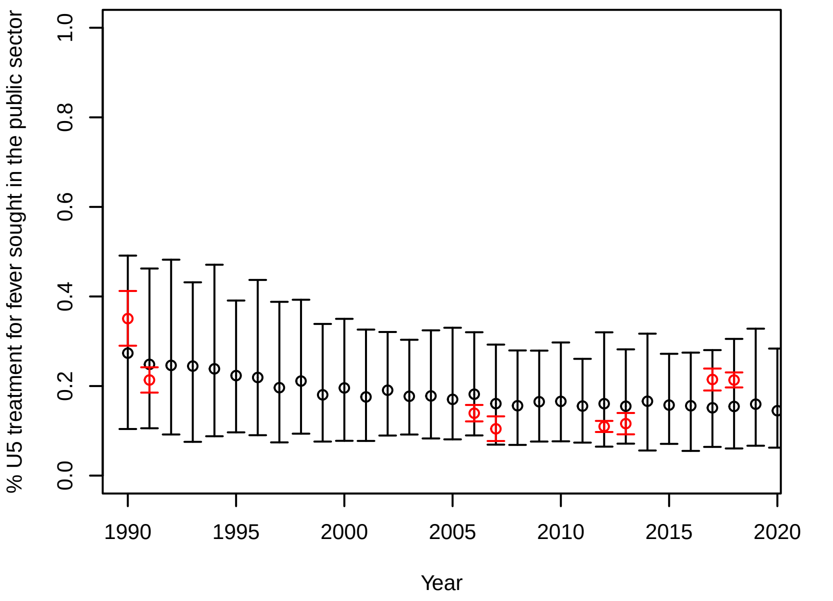

Central Sub−Saharan Africa: Angola, AGO

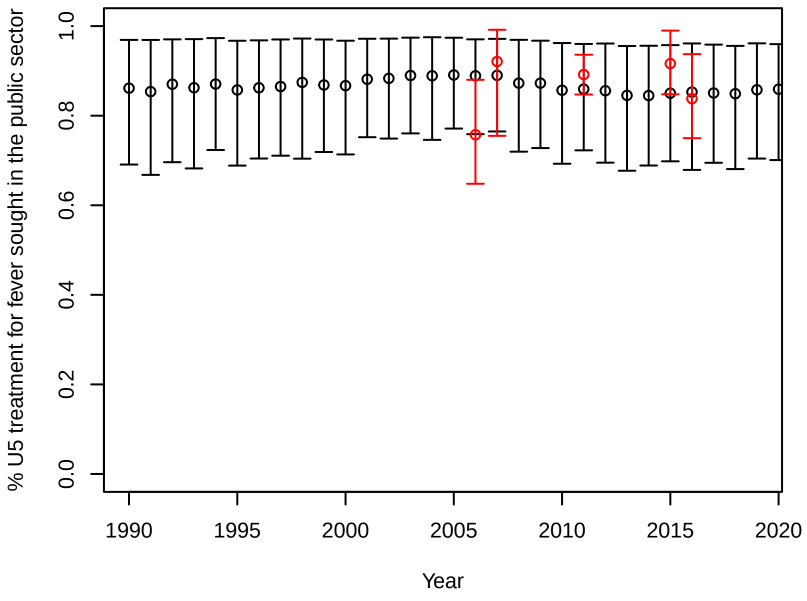

Central Sub−Saharan Africa: Central African Republic, CAF

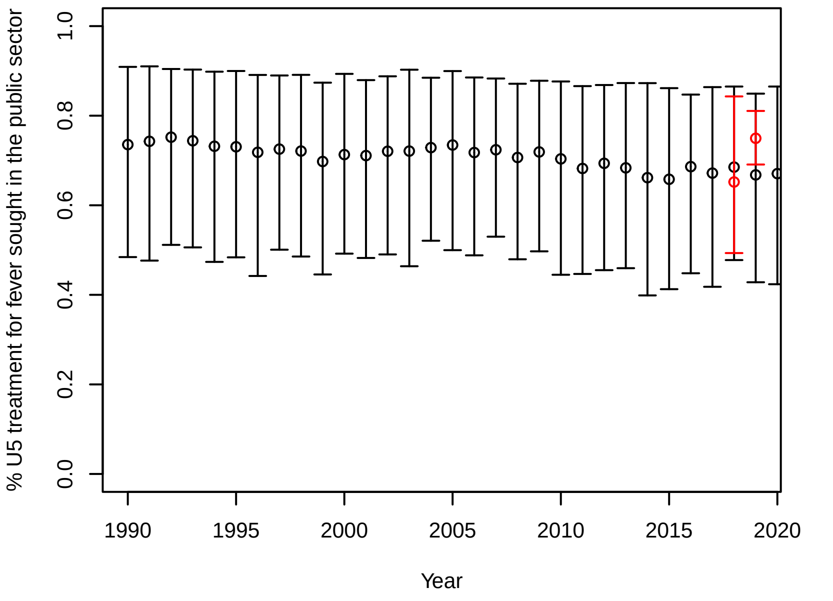

Central Sub−Saharan Africa: Congo, COG

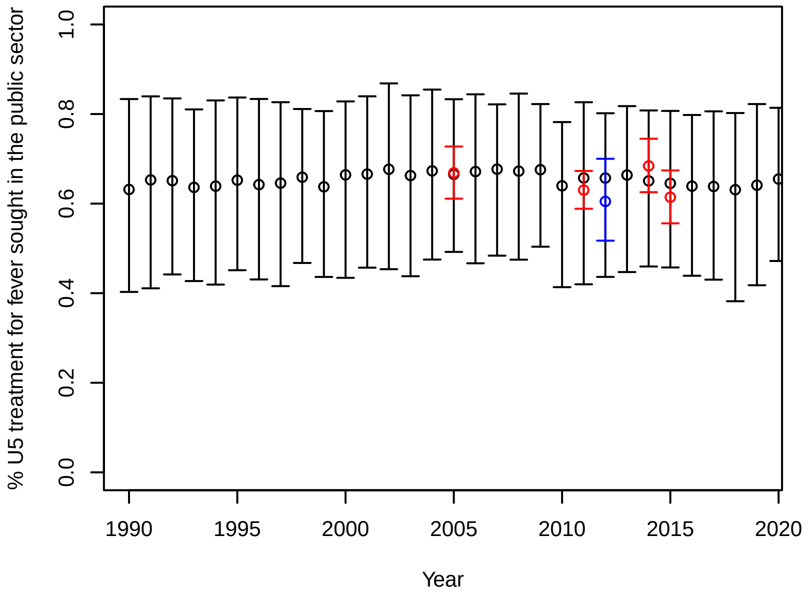

Central Sub−Saharan Africa: Democratic Republic of the Congo, C

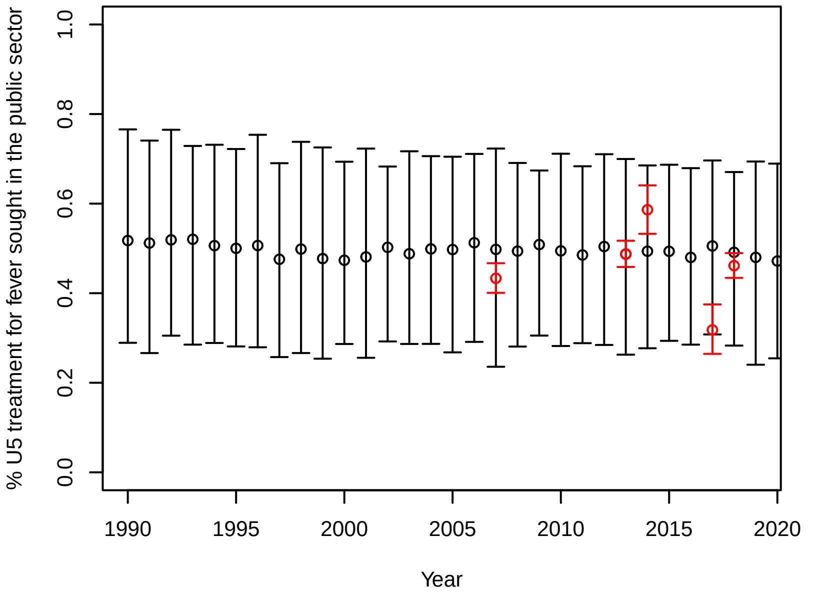

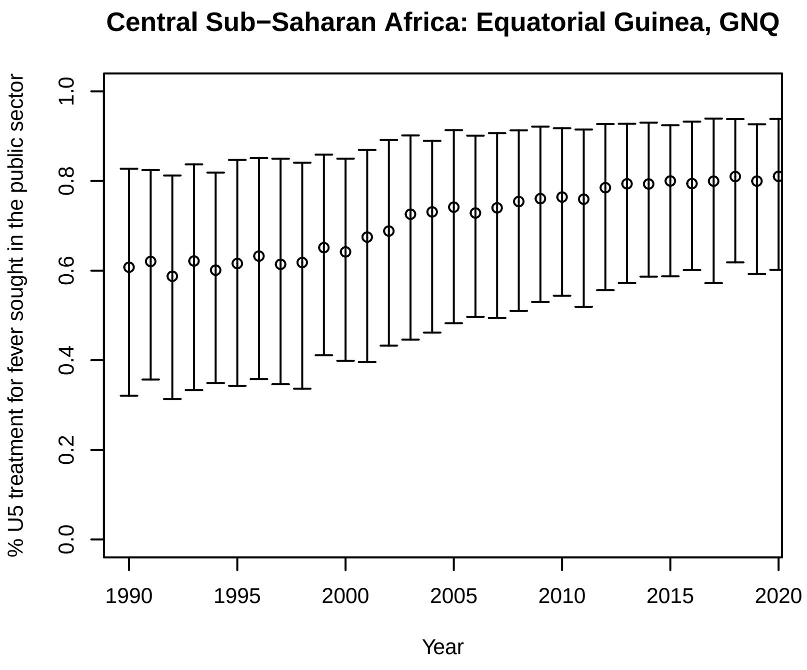
Central Sub−Saharan Africa: Equatorial Guinea, GNQ
% U5 treatment for fever sought in the public sector
Year

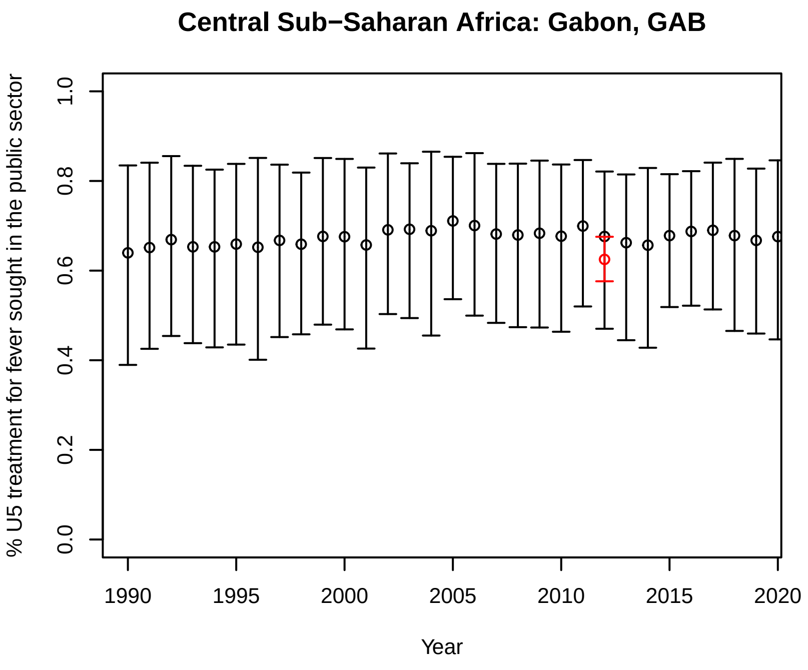
Central Sub−Saharan Africa: Gabon, GAB
% U5 treatment for fever sought in the public sector
Year

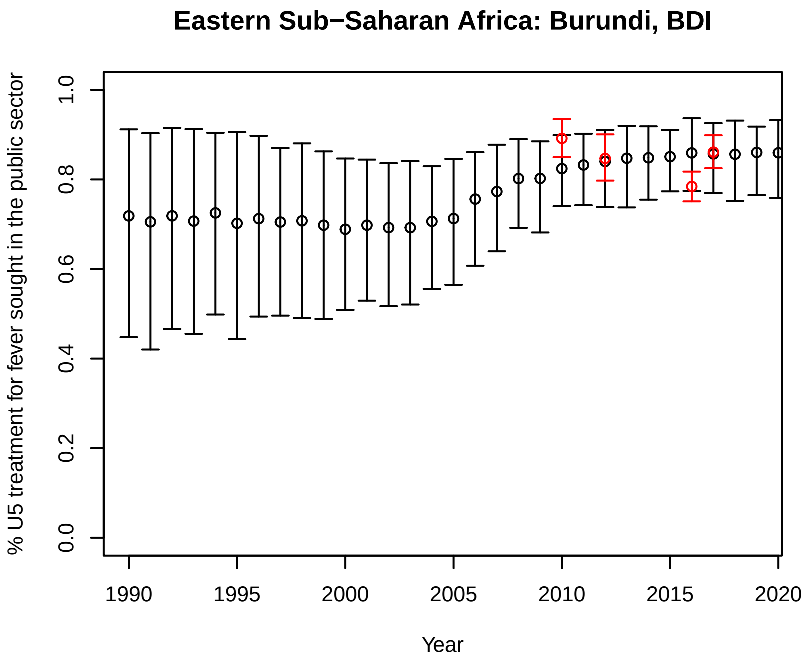
Eastern Sub−Saharan Africa: Burundi, BDI
% U5 treatment for fever sought in the public sector
Year

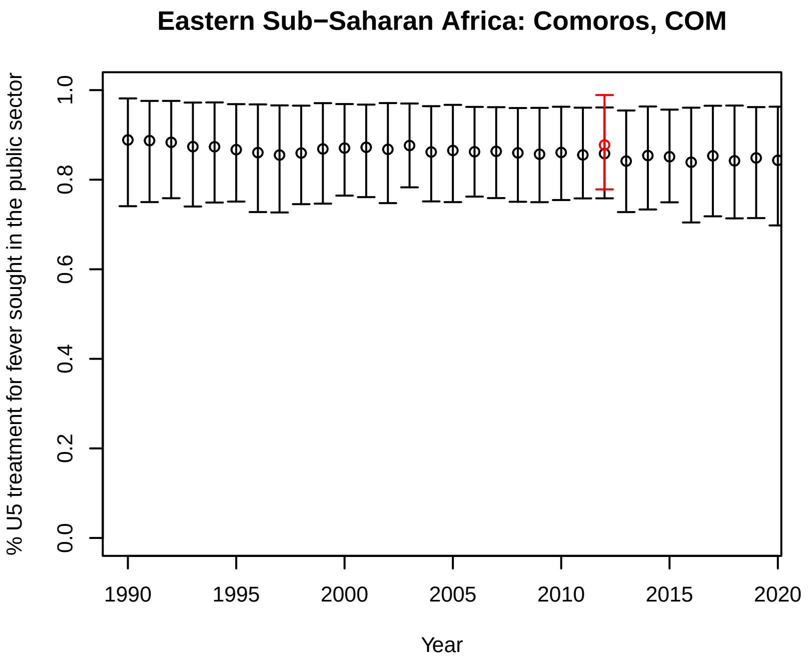
Eastern Sub−Saharan Africa: Comoros, COM
% U5 treatment for fever sought in the public sector
Year

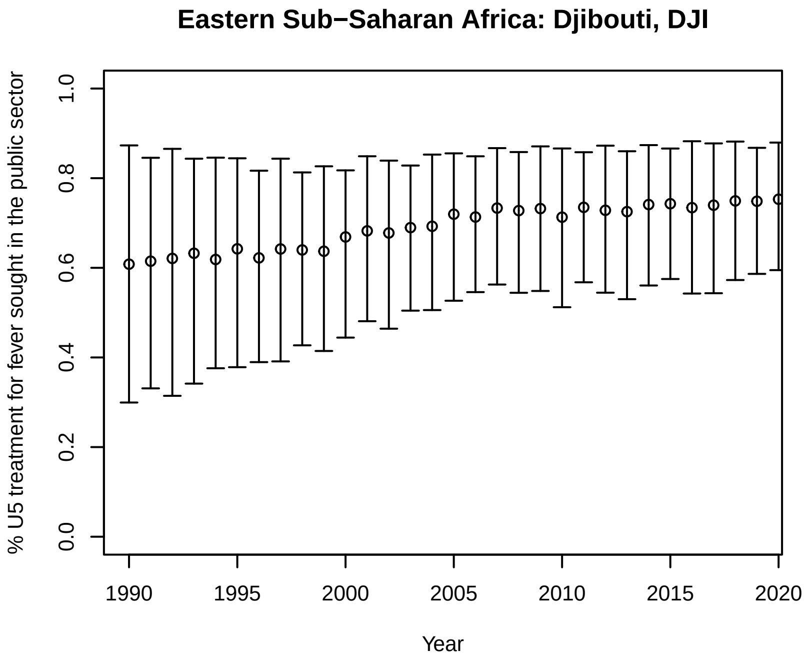
Eastern Sub−Saharan Africa: Djibouti, DJI
% U5 treatment for fever sought in the public sector
Year

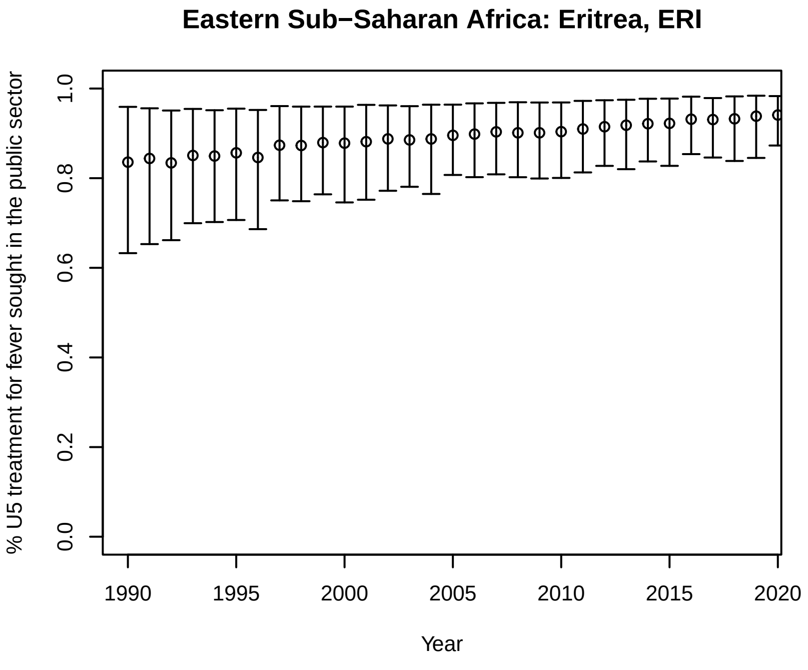
Eastern Sub−Saharan Africa: Eritrea, ERI
% U5 treatment for fever sought in the public sector
Year

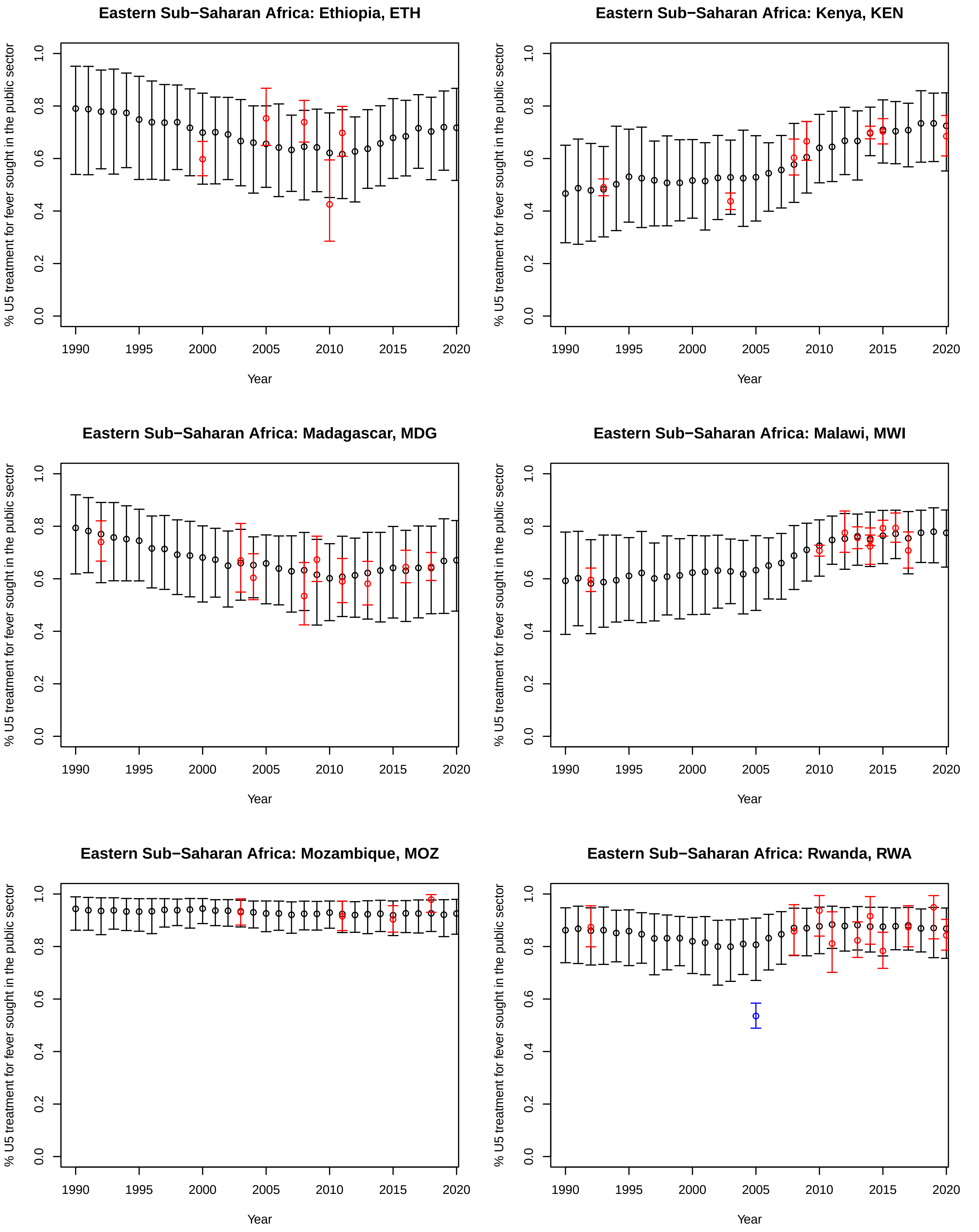
Eastern Sub−Saharan Africa: Ethiopia, ETH
Eastern Sub−Saharan Africa: Kenya, KEN
Eastern Sub−Saharan Africa: Madagascar, MDG
Eastern Sub−Saharan Africa: Malawi, MWI
Eastern Sub−Saharan Africa: Mozambique, MOZ
Eastern Sub−Saharan Africa: Rwanda, RWA
% U5 treatment for fever sought in the public sector
0.0
0.2
0.4
0.6
0.8
1.0
1990
1995
2000
2005
2010
2015
2020
Year

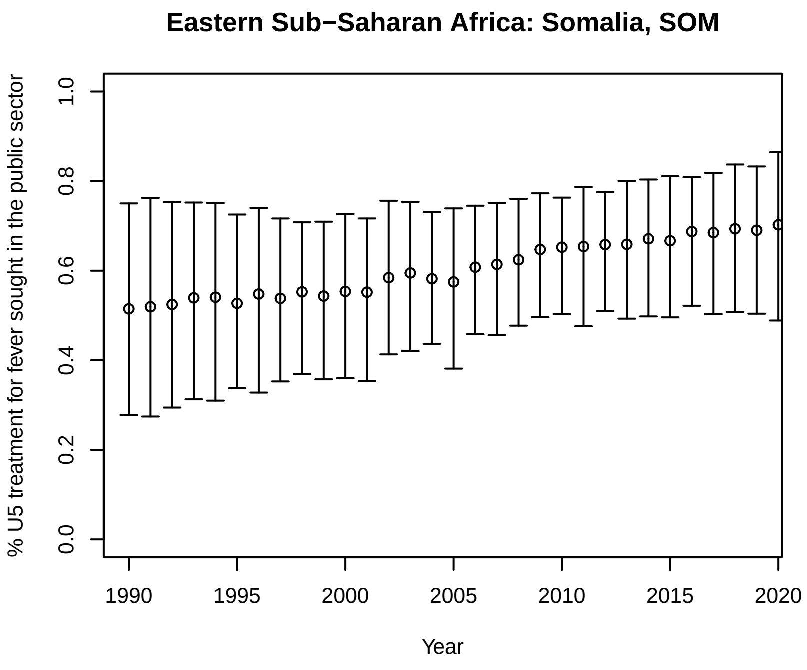
Eastern Sub−Saharan Africa: Somalia, SOM
% U5 treatment for fever sought in the public sector
Year

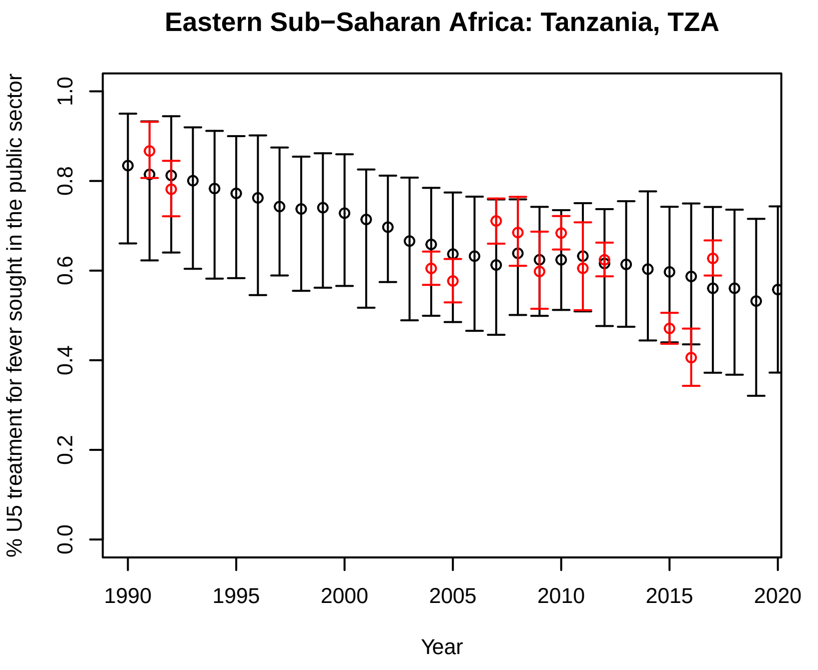
Eastern Sub−Saharan Africa: Tanzania, TZA
% U5 treatment for fever sought in the public sector
Year

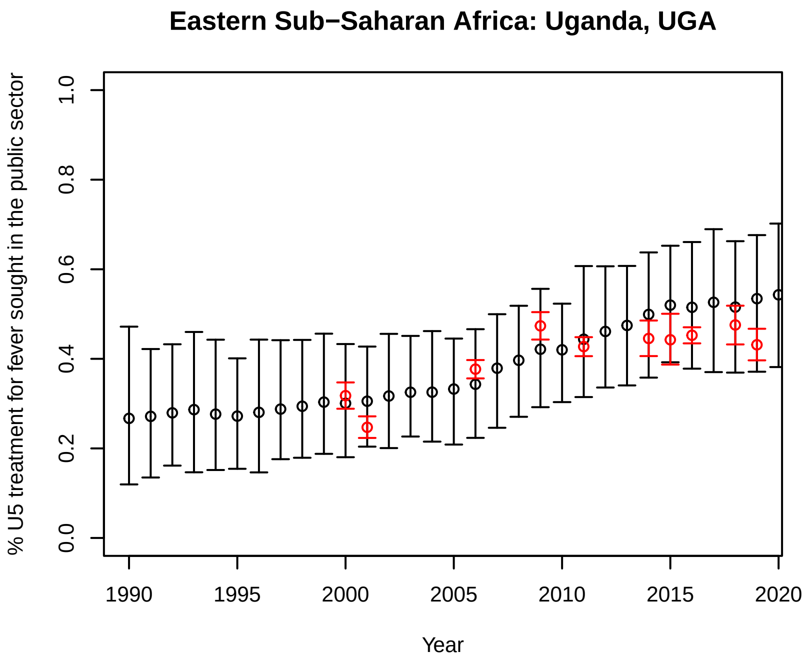
Eastern Sub−Saharan Africa: Uganda, UGA
% U5 treatment for fever sought in the public sector
Year

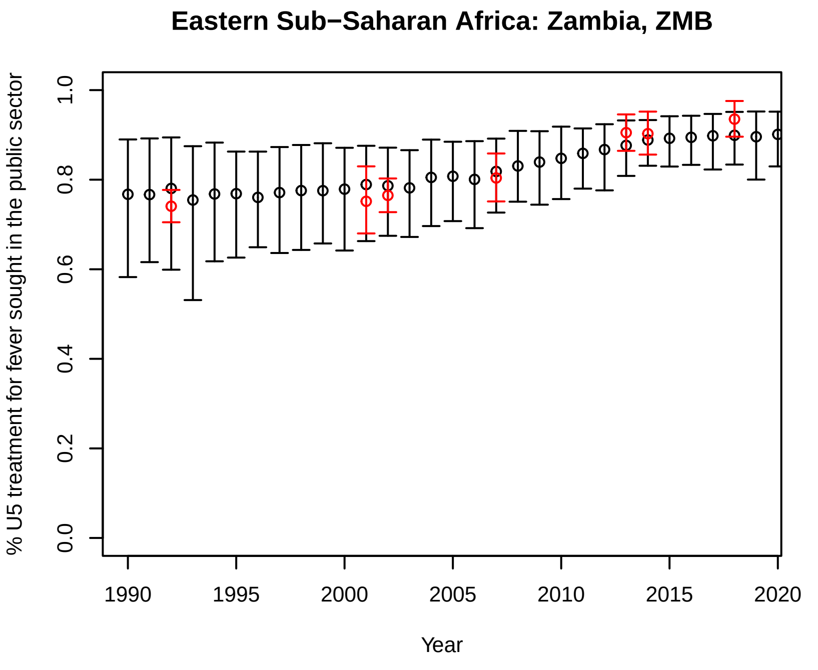
Eastern Sub−Saharan Africa: Zambia, ZMB
% U5 treatment for fever sought in the public sector
Year

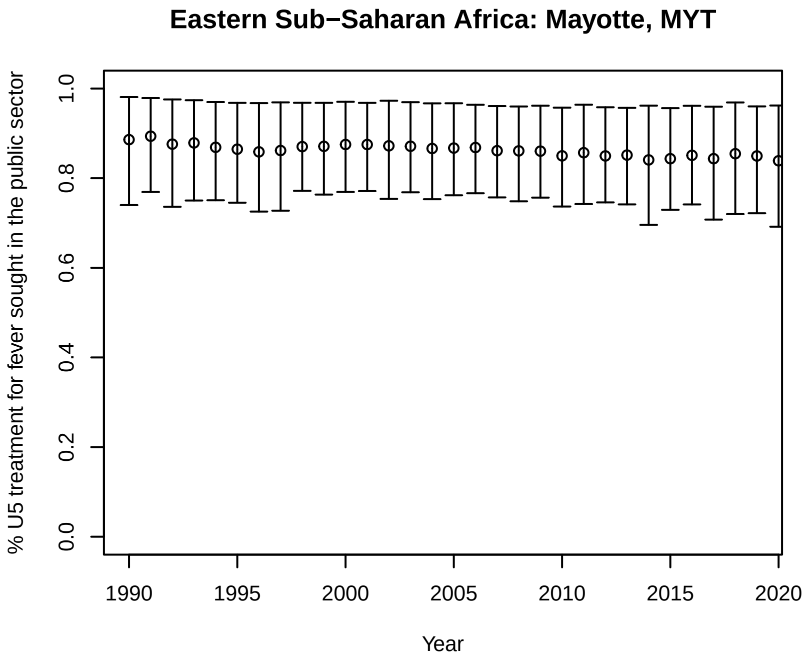
Eastern Sub−Saharan Africa: Mayotte, MYT
% U5 treatment for fever sought in the public sector
Year

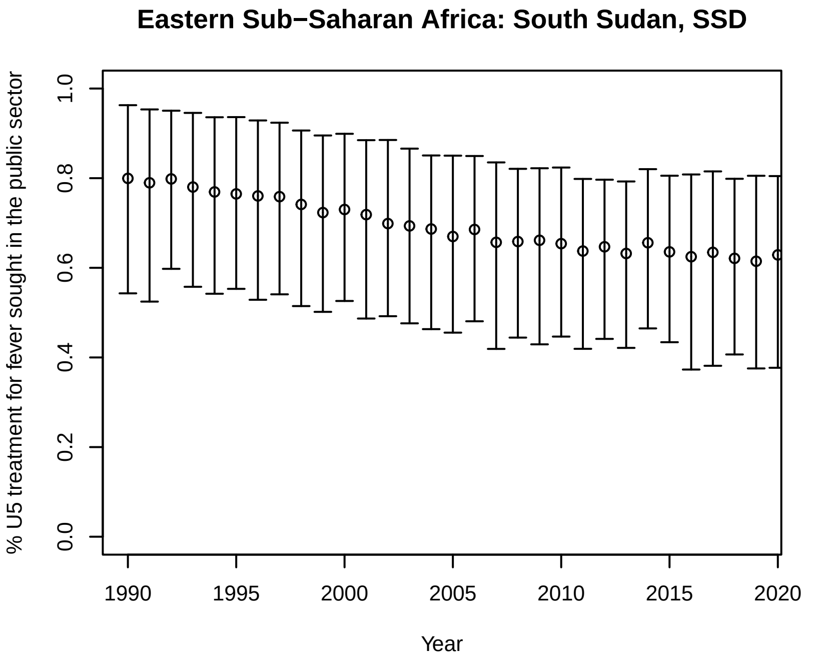
Eastern Sub−Saharan Africa: South Sudan, SSD
% U5 treatment for fever sought in the public sector
Year

Southern Sub−Saharan Africa: Botswana, BWA

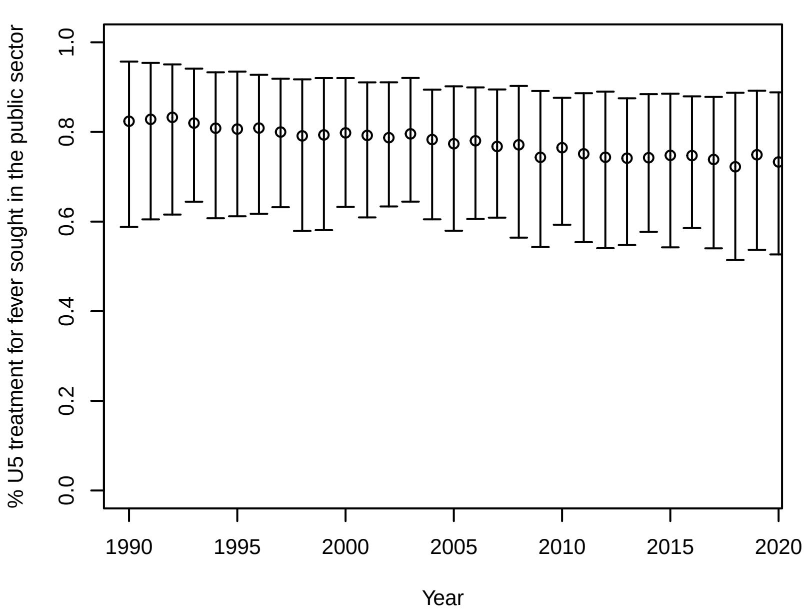

Southern Sub−Saharan Africa: Lesotho, LSO

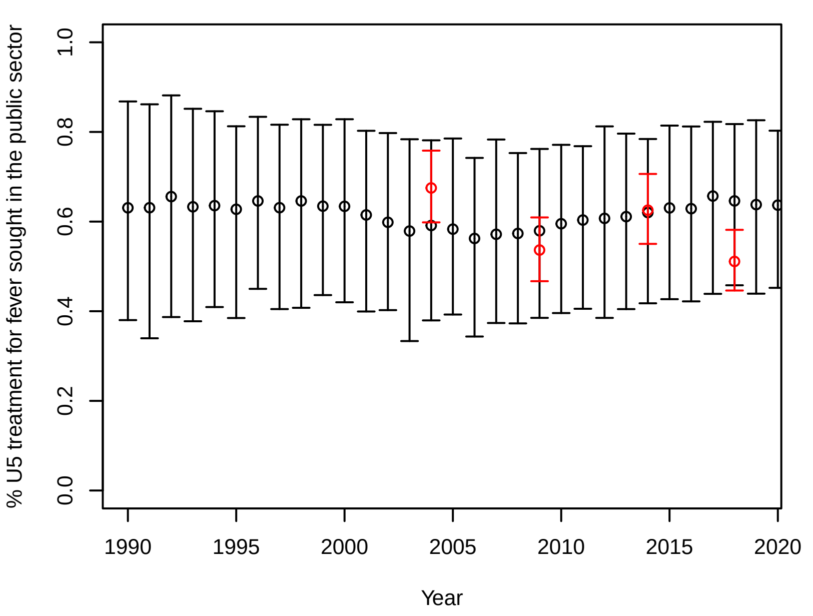

Southern Sub−Saharan Africa: Namibia, NAM

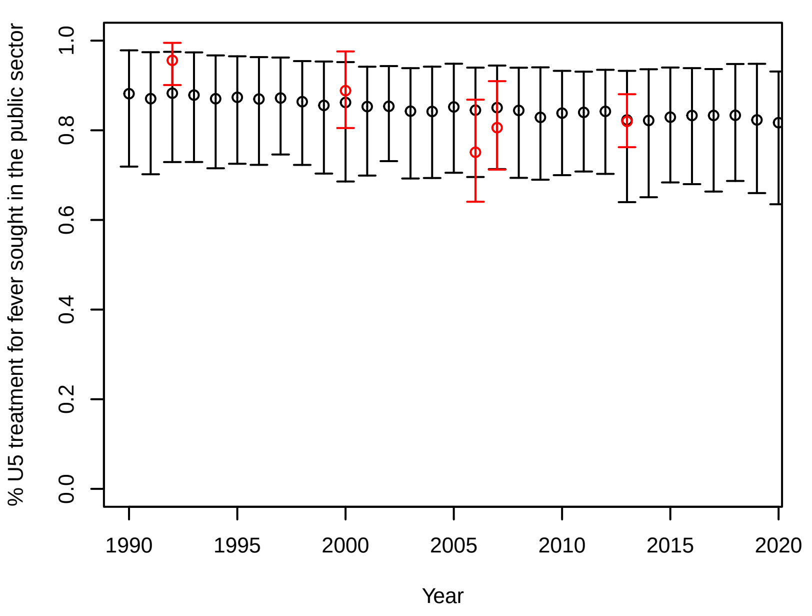

Southern Sub−Saharan Africa: South Africa, ZAF

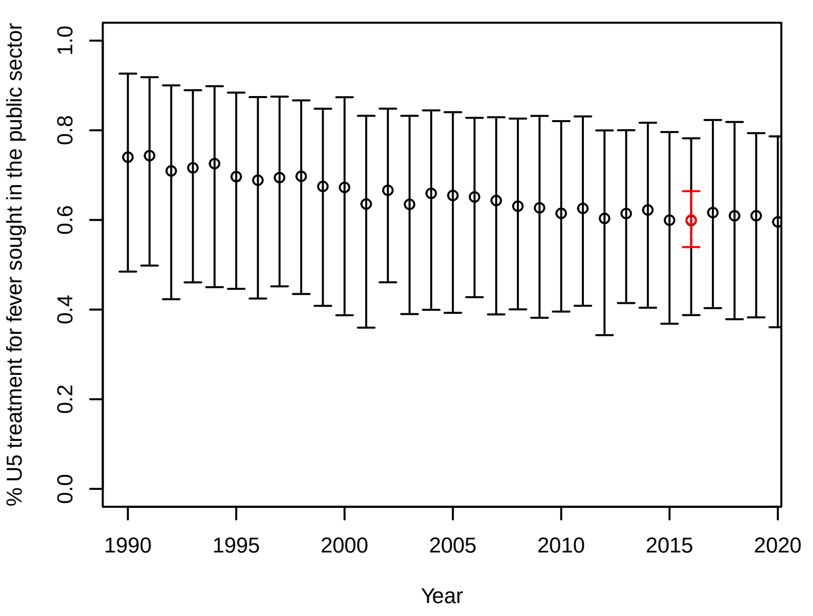

Southern Sub−Saharan Africa: Eswatini, SWZ

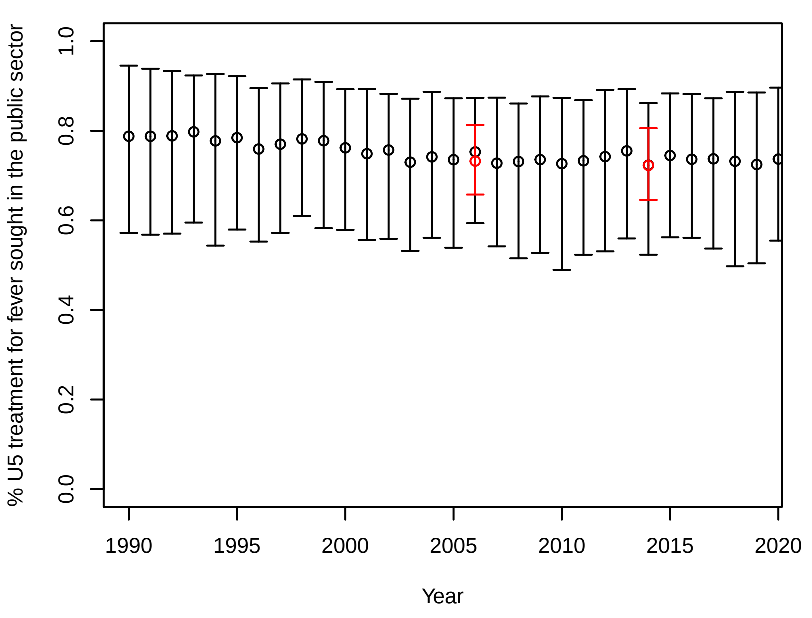

Southern Sub−Saharan Africa: Zimbabwe, ZWE

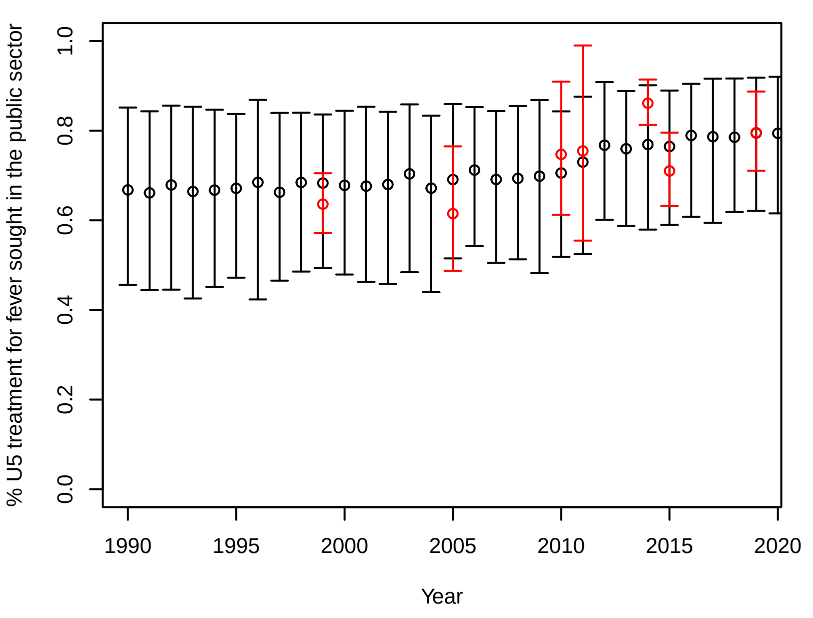

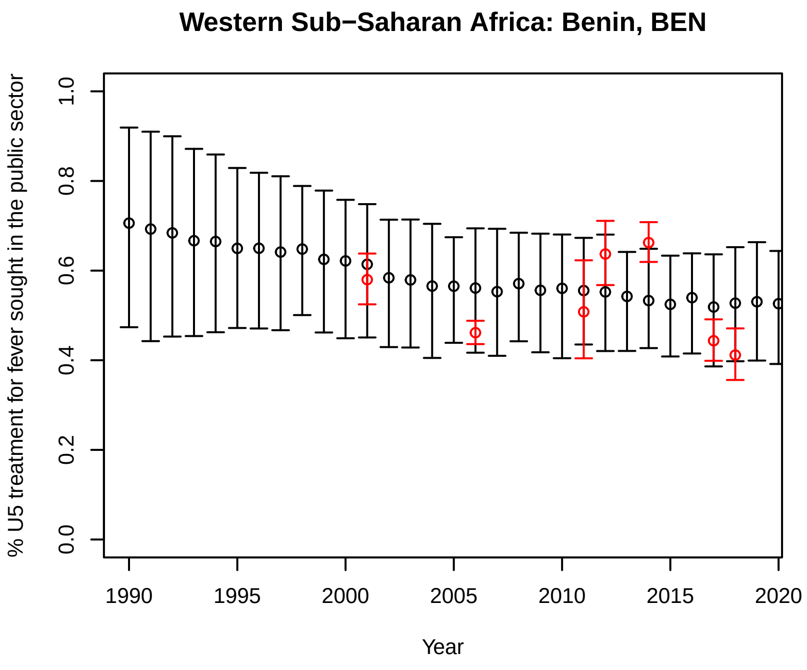
Western Sub−Saharan Africa: Benin, BEN
% U5 treatment for fever sought in the public sector
Year
0.0
0.2
0.4
0.6
0.8
1.0
1990
1995
2000
2005
2010
2015
2020

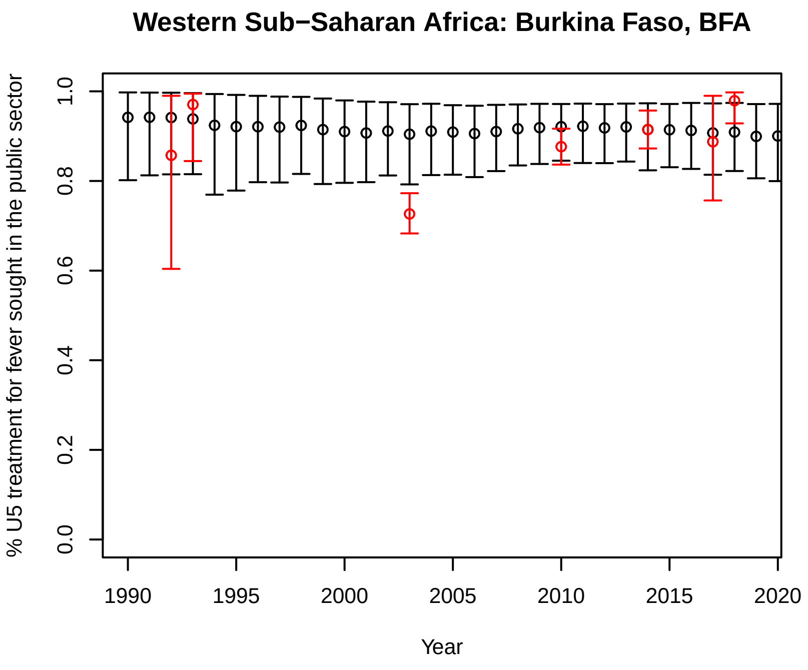
Western Sub−Saharan Africa: Burkina Faso, BFA
% U5 treatment for fever sought in the public sector
Year
0.0
0.2
0.4
0.6
0.8
1.0
1990
1995
2000
2005
2010
2015
2020

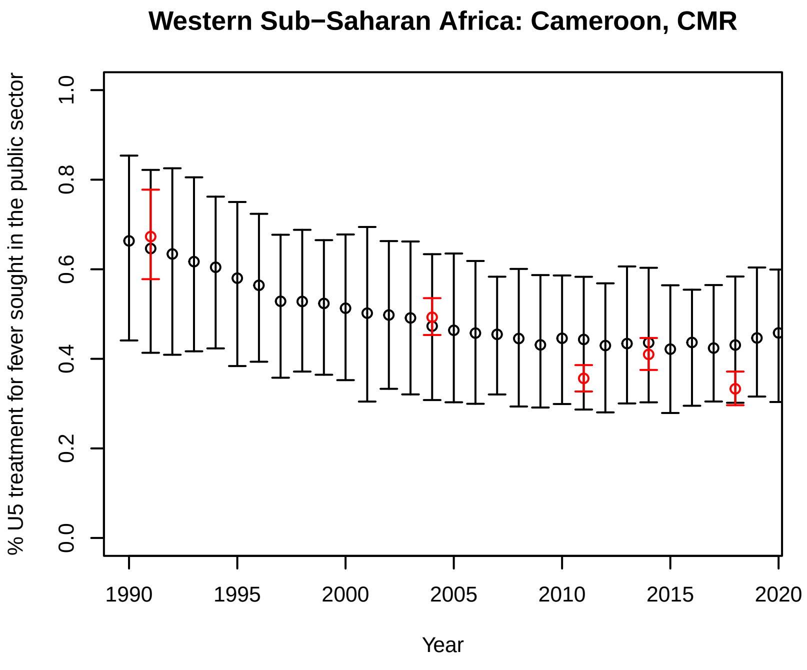
Western Sub−Saharan Africa: Cameroon, CMR
% U5 treatment for fever sought in the public sector
Year
0.0
0.2
0.4
0.6
0.8
1.0
1990
1995
2000
2005
2010
2015
2020

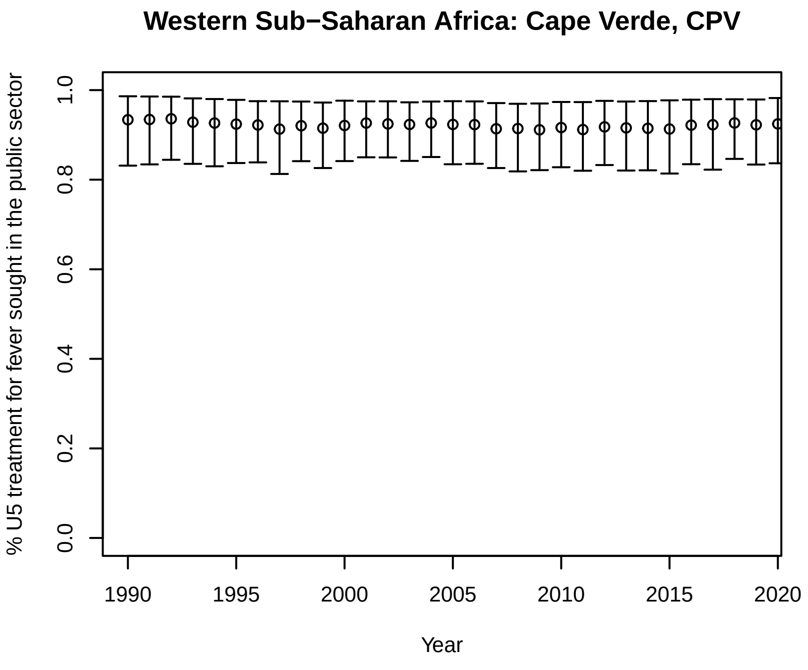
Western Sub−Saharan Africa: Cape Verde, CPV
% U5 treatment for fever sought in the public sector
Year
0.0
0.2
0.4
0.6
0.8
1.0
1990
1995
2000
2005
2010
2015
2020

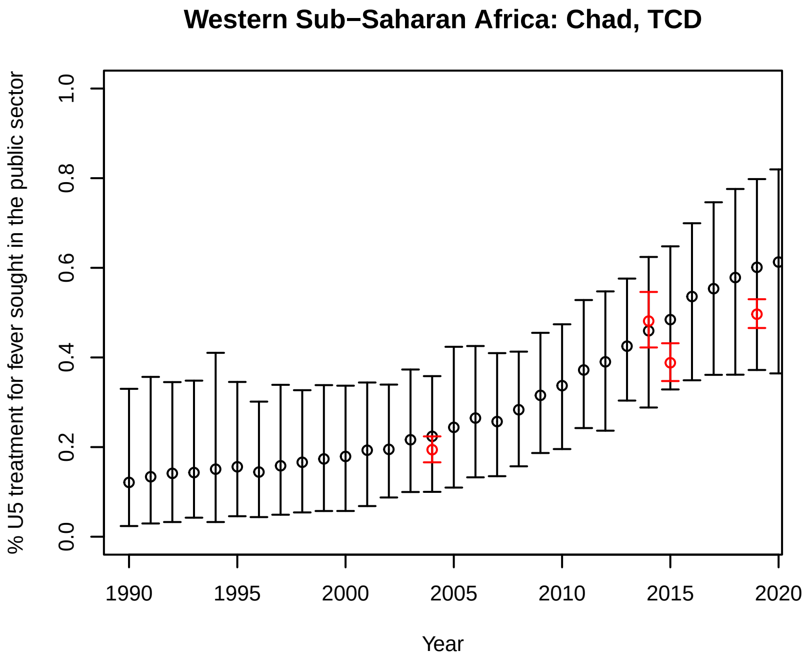
Western Sub−Saharan Africa: Chad, TCD
% U5 treatment for fever sought in the public sector
Year
0.0
0.2
0.4
0.6
0.8
1.0
1990
1995
2000
2005
2010
2015
2020

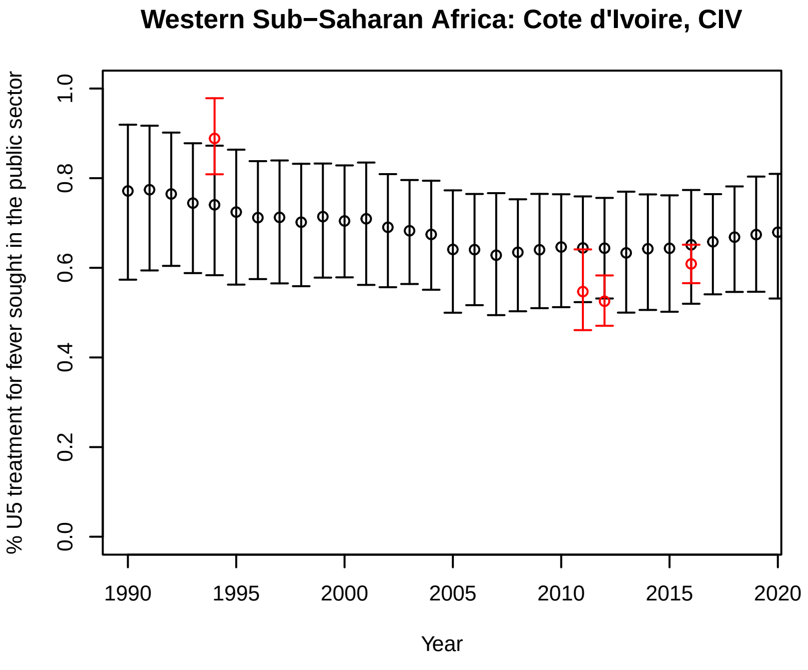
Western Sub−Saharan Africa: Cote d'Ivoire, CIV
% U5 treatment for fever sought in the public sector
Year
0.0
0.2
0.4
0.6
0.8
1.0
1990
1995
2000
2005
2010
2015
2020

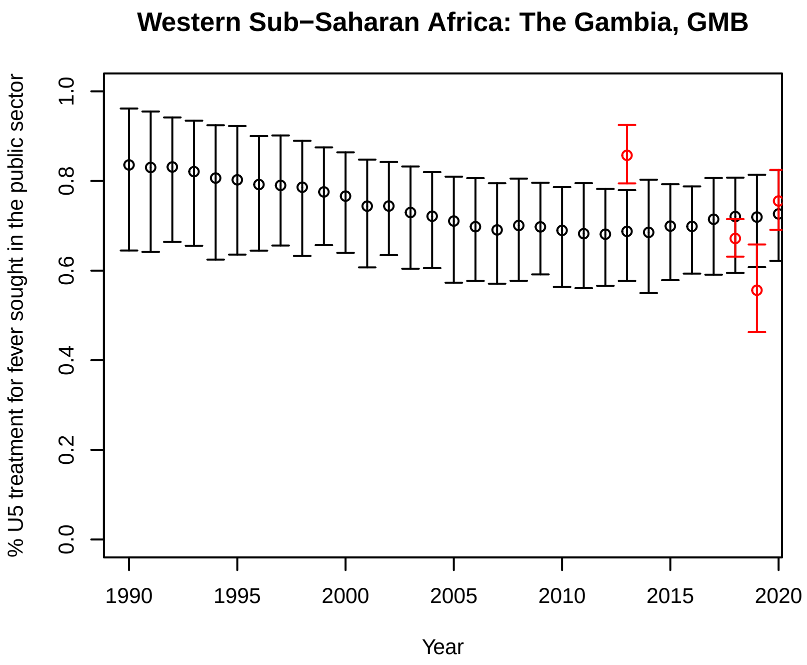
Western Sub−Saharan Africa: The Gambia, GMB
% U5 treatment for fever sought in the public sector
0.0
0.2
0.4
0.6
0.8
1.0
1990
1995
2000
2005
2010
2015
2020
Year

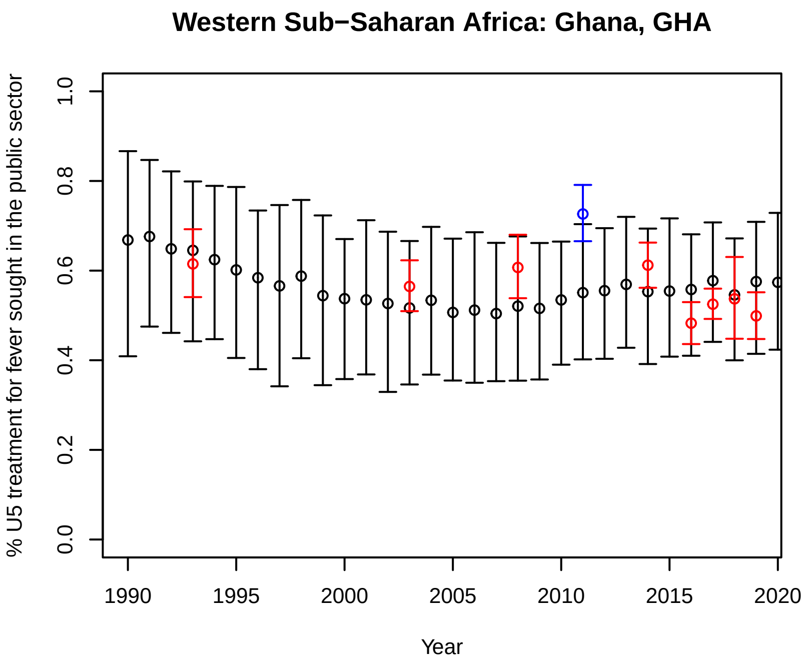
Western Sub−Saharan Africa: Ghana, GHA
% U5 treatment for fever sought in the public sector
0.0
0.2
0.4
0.6
0.8
1.0
1990
1995
2000
2005
2010
2015
2020
Year

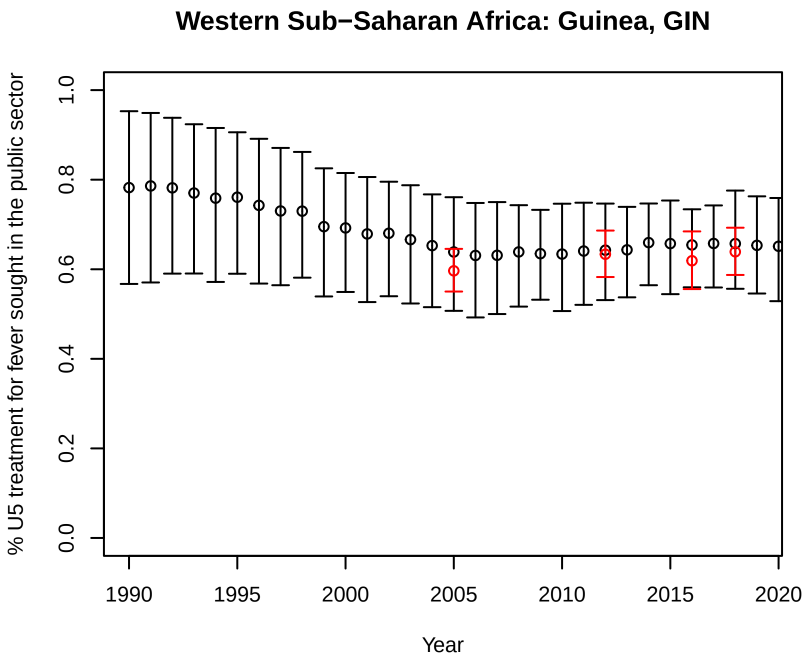
Western Sub−Saharan Africa: Guinea, GIN
% U5 treatment for fever sought in the public sector
0.0
0.2
0.4
0.6
0.8
1.0
1990
1995
2000
2005
2010
2015
2020
Year

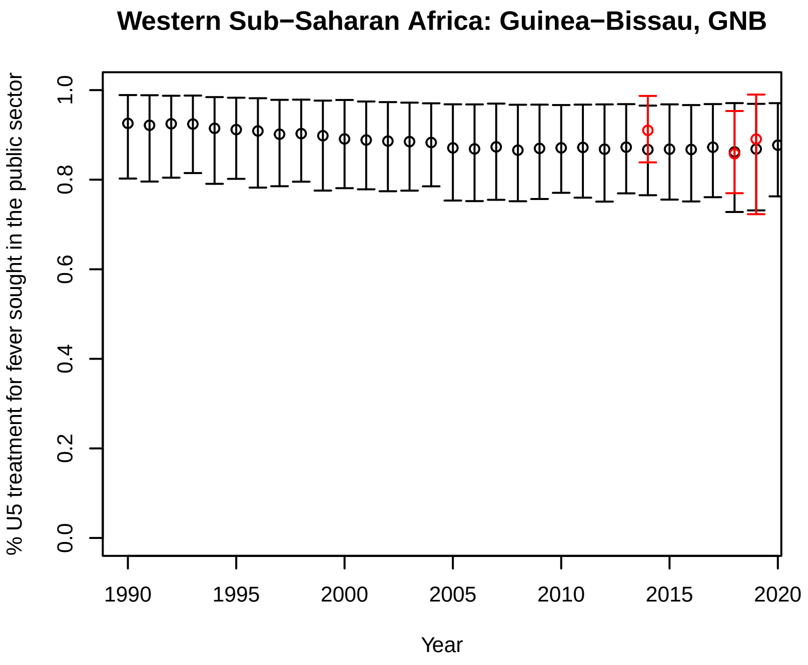
Western Sub−Saharan Africa: Guinea−Bissau, GNB
% U5 treatment for fever sought in the public sector
0.0
0.2
0.4
0.6
0.8
1.0
1990
1995
2000
2005
2010
2015
2020
Year

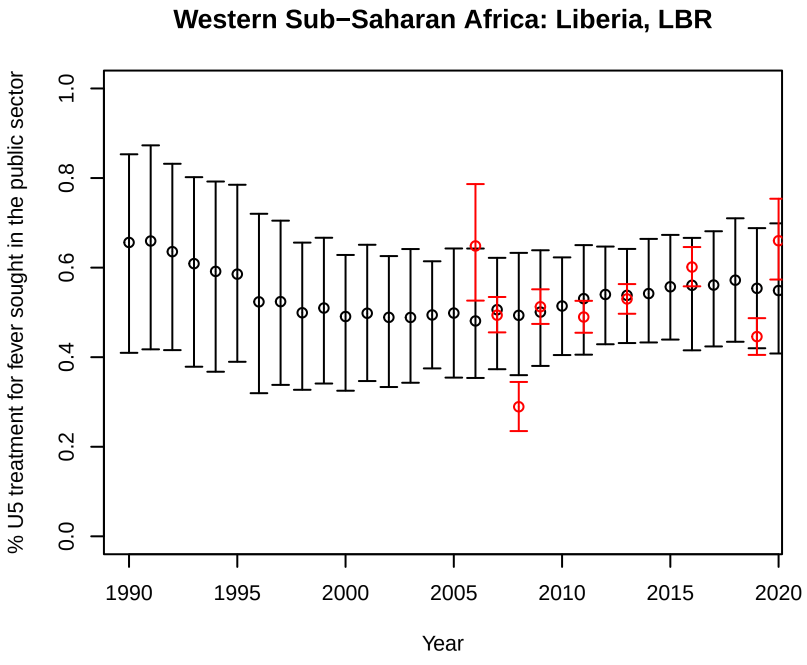
Western Sub−Saharan Africa: Liberia, LBR
% U5 treatment for fever sought in the public sector
0.0
0.2
0.4
0.6
0.8
1.0
1990
1995
2000
2005
2010
2015
2020
Year

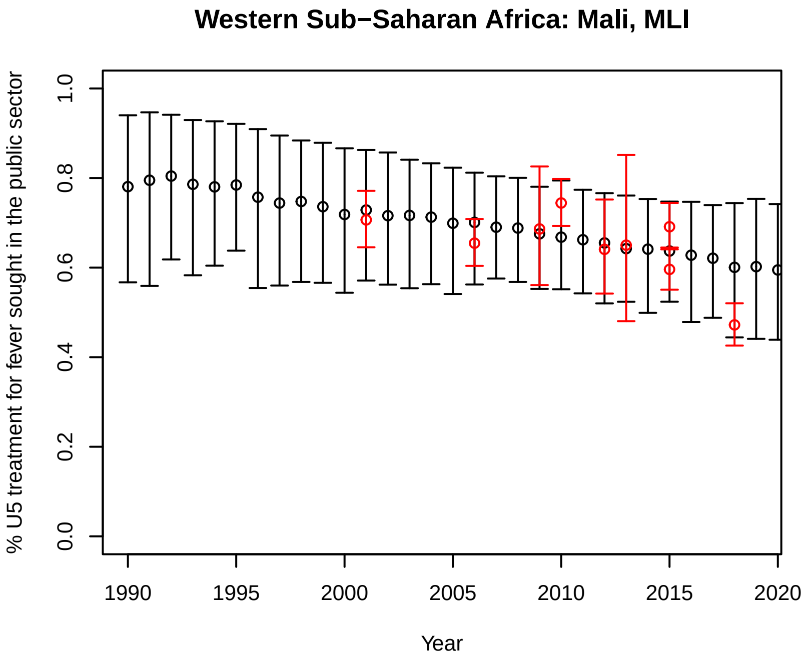
Western Sub−Saharan Africa: Mali, MLI
% U5 treatment for fever sought in the public sector
0.0
0.2
0.4
0.6
0.8
1.0
1990
1995
2000
2005
2010
2015
2020
Year

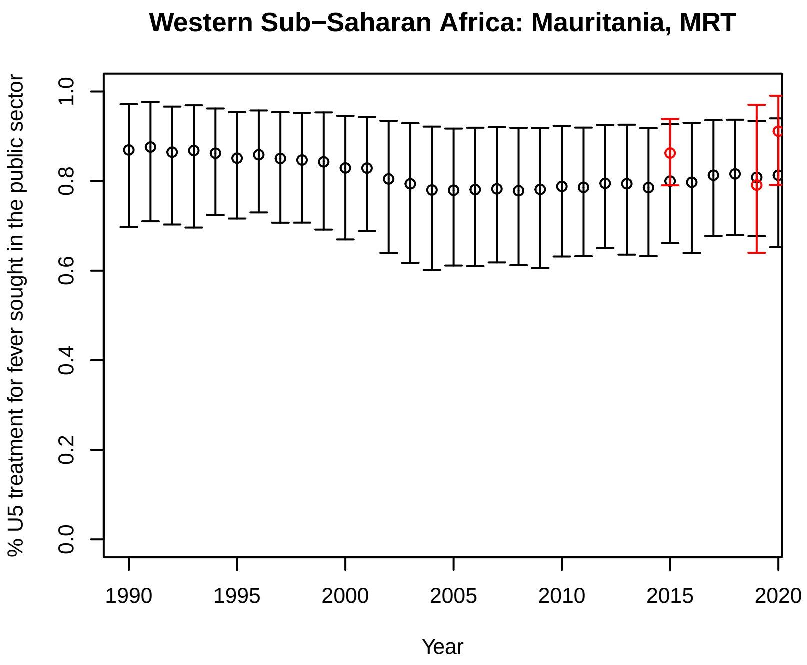
Western Sub−Saharan Africa: Mauritania, MRT
% U5 treatment for fever sought in the public sector
0.0
0.2
0.4
0.6
0.8
1.0
1990
1995
2000
2005
2010
2015
2020
Year

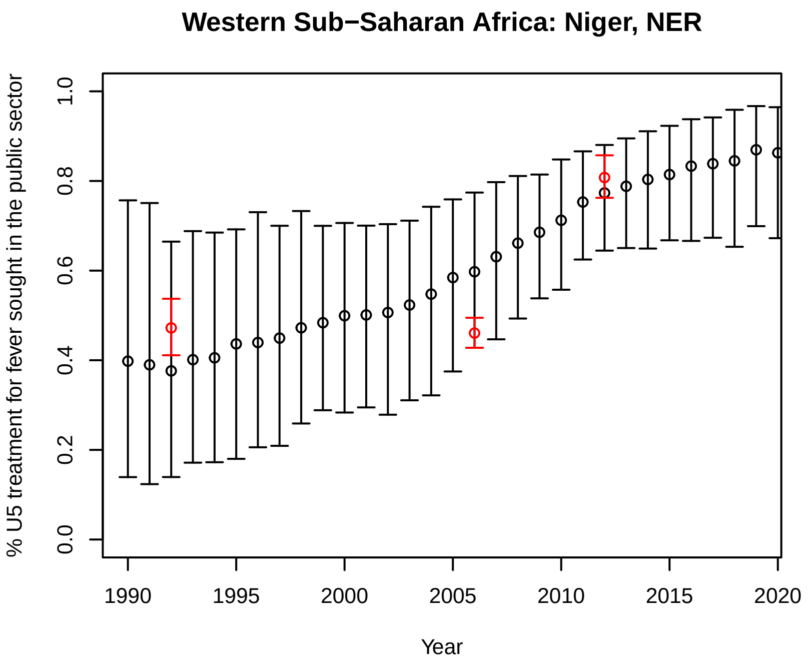
Western Sub−Saharan Africa: Niger, NER
% U5 treatment for fever sought in the public sector
0.0
0.2
0.4
0.6
0.8
1.0
1990
1995
2000
2005
2010
2015
2020
Year

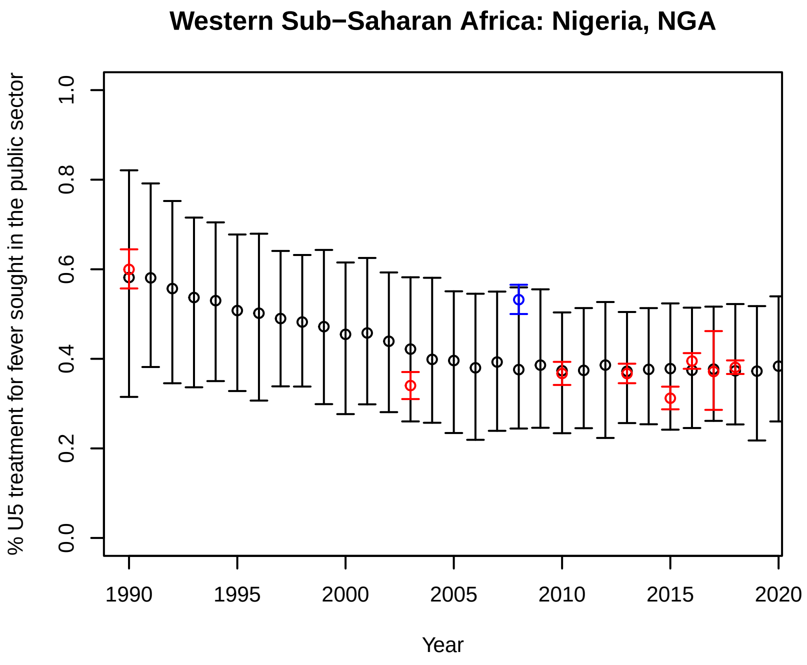
Western Sub−Saharan Africa: Nigeria, NGA
% U5 treatment for fever sought in the public sector
0.0
0.2
0.4
0.6
0.8
1.0
1990
1995
2000
2005
2010
2015
2020
Year

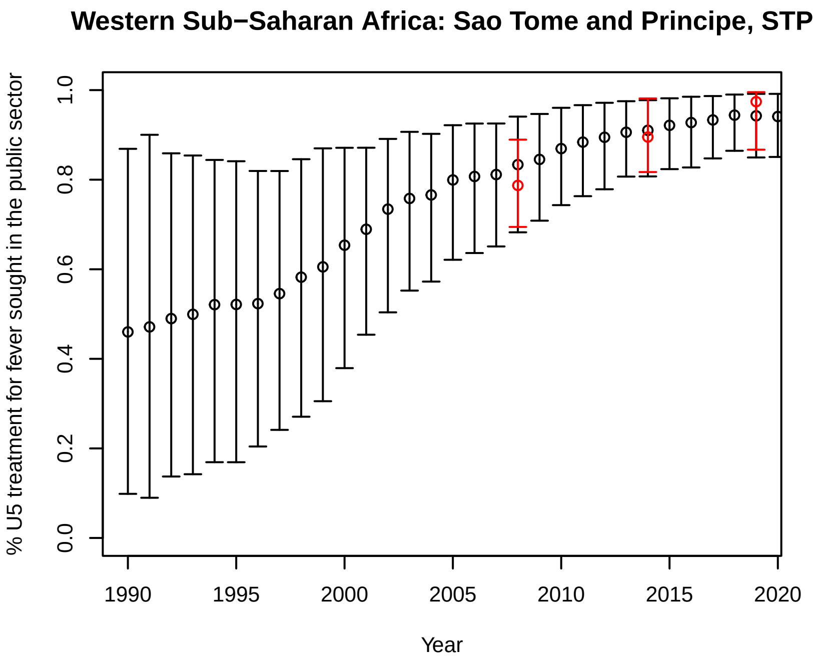
Western Sub−Saharan Africa: Sao Tome and Principe, STP
% U5 treatment for fever sought in the public sector
0.0
0.2
0.4
0.6
0.8
1.0
1990
1995
2000
2005
2010
2015
2020
Year

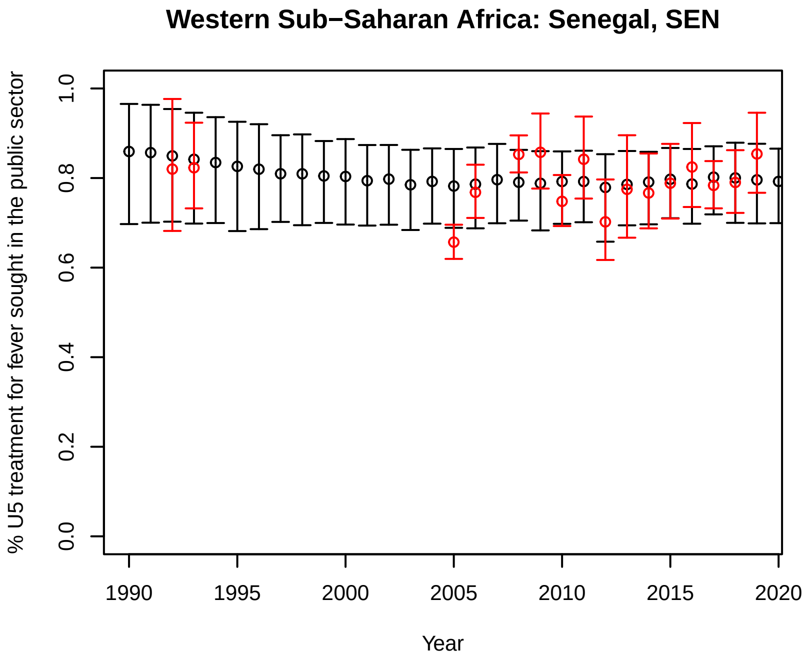
Western Sub−Saharan Africa: Senegal, SEN
% U5 treatment for fever sought in the public sector
0.0
0.2
0.4
0.6
0.8
1.0
1990
1995
2000
2005
2010
2015
2020
Year

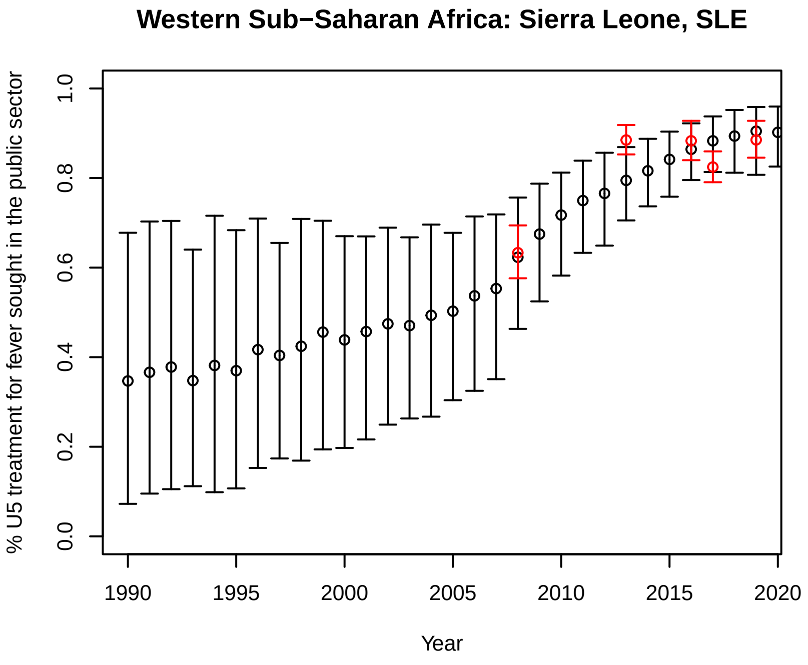
Western Sub−Saharan Africa: Sierra Leone, SLE
% U5 treatment for fever sought in the public sector
0.0
0.2
0.4
0.6
0.8
1.0
1990
1995
2000
2005
2010
2015
2020
Year

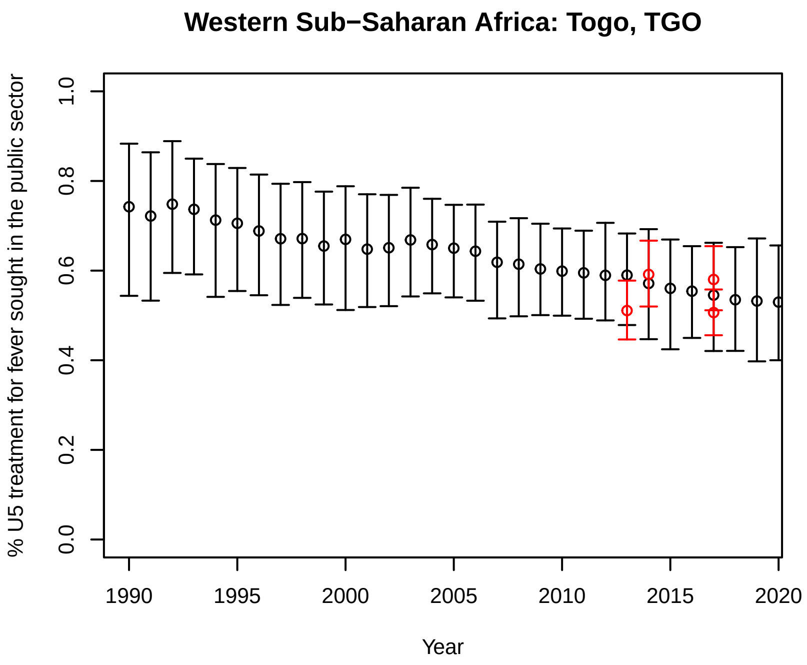

Western Sub−Saharan Africa: Togo, TGO
% U5 treatment for fever sought in the public sector
0.0
0.2
0.4
0.6
0.8
1.0
1990
1995
2000
2005
2010
2015
2020
Year
