# Supplementary material for: Trends in treatment-seeking for fever in children under five years old in 151 countries from 1990 to 2020
Source: PLOS Glob Public Health. 2023 Aug 23;3(8):e0002134. doi: 10.1371/journal.pgph.0002134 (PMC10446233; doi:10.1371/journal.pgph.0002134)
Supplement: S1 Appendix — A detailed description of the modelling process employed to derive the estimates presented in this study. (DOCX) [file pgph.0002134.s006.docx]

**S1 Appendix for: Trends in treatment-seeking for fever in children under five years old in 151 countries from 1990 to 2020**

This document contains the modelling details for the treatment-seeking rates for children under five with fever at any point of care (any treatment-seeking) and fraction seeking treatment in the public sector (public fraction).

# The data

Treatment-seeking data was obtained from Demographic Health Surveys (DHS), Malaria Indicator Surveys (MIS), AIDS Indicator Surveys (AIS) and Multiple Indicator Cluster Surveys (MICS), for all countries in Africa, Latin America, the Caribbean, and Asia (excluding Russia, Australasia, and Western Sahara). The surveys included in this study were conducted between 1990 and 2021.The data collected was limited to children under 5 years old for which the response to: ‘Has (NAME) been ill with a fever at any time in the last 2 weeks?' was ‘YES’. With this limit in place, we looked at the questions ‘Did you seek advice or treatment for the fever from any source?’ and ‘Where did you get treatment from?’. All location responses were classified into no treatment, nonmedical treatment (e.g., traditional practitioner, friend, priest), any medical treatment, and public medical treatment. The classified responses were then aggregated by year of the interview to national levels using sampling weights, resulting in national-level estimates of treatment-seeking rates in the year of the survey.

All countries included in this study were grouped into geographical regions and super-regions (see Figure 1 in the main paper).

A number of outliers were identified during dataset inspection and removed prior to the modelling process. For the ‘any medical treatment model’, the outliers were: Nigeria 2008 DHS, Democratic Republic of The Congo 2001 MICS, Timor-Leste 2016 DHS, Ghana 2011 MICS, Republic of Congo 2012 DHS. For the public fraction model, the following surveys were removed: Nigeria 2008 DHS, Democratic Republic of The Congo 2001 MICS, Rwanda 2005 DHS, Gambia 2000 MICS, Mozambique 2008 MICS and Georgia 2018 MICS.

# The covariates

Based on a previous literature review by Battle et.al [1], we tested the following IHME covariates (these have been normalised using transformations selected via the Box-Cox transformation):

- ANC1_coverage_prop: Proportion of pregnant women receiving any antenatal care from a skilled provider,
- ANC4_coverage_prop: Proportion of pregnant woman receiving 4 or more antenatal care visits including 1 or more from a skilled provider,
- DTP3_coverage_prop: Fraction of children born in a given country-year who have received 3 doses of DTP3,
- hospital_beds_per1000: Number of hospital beds per 1000 people,
- IFD_coverage_prop: Percent of women giving birth in a health facility,
- LDI_pc: Gross domestic product per capita that has been smoothed over the preceding 10 years,
- measles_vacc_cov_prop: Fraction of children born in a given country-year who have received a measles vaccination,
- SBA_coverage_prop: Percent of women giving birth with a skilled birth attendant (mainly nurses, doctors, midwives),
- GDPpc_id_b2010: GDP per capita base 2010 international dollars,
- prop_urban: Urbanicity,
- he_cap: Health expenditure per capita,
- ind_health: The proportion of the employed population ages 15-69 working in health and social work (according to ISIC classifications),
- education_all_ages_and_sexes_pc: Education (years per capita) aggregated by age (15+) and sex,
- log_the_pc: Log-transformed national-level estimates for total health expenditure per capita.

Several population-weighted static covariates were also considered:

- DMSP_nighttime: Population weighted DMSP nighttime lights in 2010 [2]
- VIIRS_nighttime: Population weighted VIIRS nighttime lights in 2012 [3]
- accessibility: Population weighted time to the nearest city in 2015 [4]
- healthcare accessibility: Population weighted time to the nearest health facility in 2019 [5].

Note that nighttime lights were considered as a proxy for urbanicity. Since French Guiana and Mayotte do not have IHME covariates, we used the covariates corresponding to Suriname and Comoros for them respectively.

# The models

Generalised additive mixed models (GAMMs) were used to smooth and estimate the rates using region-specific intercepts and trends, unit-level random effects as well as non-linear relationships with socio-economic covariates from the Institute for Health Metrics and Evaluation (IHME) and the Malaria Atlas Project (MAP).

Two separate GAMMs were constructed for any treatment-seeking rates as well as the fraction seeking treatment at a public health facility (public fraction). The public treatment-seeking rates were later computed by multiplying the two. Each GAMM took the form of:

|  | $logit\left( Y \right)=\beta_{country}+\beta_{region}+s_{region}\left( year \right)+\sum s_{k}\left( x_{k,} \right)+\epsilon$, | (1) |
| --- | --- | --- |

where Y denotes the estimated any treatment-seeking rate or the estimated public fraction. *β_region_* is a region-specific intercept, *β_country_* is the country random effect, *x_k_* is the mean of the *k*^th^ covariate, and $\epsilon$ denotes the Gaussian random error. Splines, which are denoted by *s,* allowed for non-linear region-specific temporal trends and effects of the covariates. To prevent overfitting and to facilitate interpretation, the maximum basis dimensions of the splines were set to 5 for the region-specific trends and 3 for the non-linear covariate effects. The logit transformation was chosen to map the rates and fractions from (0, 1) to real numbers for modelling purposes.

# Model fitting

To select our models, we separate the data into training and test sets. If the region had more than eight data points, we selected ~70% randomly to include in the training set. Otherwise, we included all data corresponding to the region in the training data. Because the data from pre 2000s was limited, to ensure that 1990s data doesn’t get lost in random sampling, we split the dataset into pre and post 2000s before randomly choosing ~70% points from each subset.

Model selection was conducted using the training data. Since there was not enough data to properly estimate all region-specific intercepts and trends, we only included the model components if they were significant. For the intercepts, this was judged based on their relative levels and p-values; for the trends, this was judged by cross-referencing the plots of the estimated smooth terms with their estimated p-values. With the region-specific intercepts and trends in place, we fit one GAMM per covariate. A set of covariates was collated by ranking the Akaike Information Criterion (AIC) values of their individual fitted models and including a covariate if they have less than 60% correlation with the covariates which are already chosen. In this way, we prioritised the covariates most highly associated with the modelled variables while reducing multicollinearity.

The GAMM with the selected region-specific intercepts and trends, as well as the reduced set of covariates formed a metamodel. Subsets of the metamodel were ranked based on AICs (AIC with a correction for small sample sizes) using the ‘MuMIn' R package [6]. For both any treatment-seeking and public fractions, the models with the lowest AICs were chosen.

The estimated terms of the best any treatment-seeking and public fraction model are given in Table 1 and Table 2, respectively. The plots of the corresponding splines are shown in Fig 1 and Fig 2.

**Table 1.** **Summary of best any treatment-seeking model with pruned region temporal trends and region factors as well as random effects for the units.**

| **A. Regional intercepts** | **Estimate** | **Std. Error** | **t-value** | **p-value** |
| --- | --- | --- | --- | --- |
| Other | 0.0413 | 0.1585 | 0.2607 | 0.7945 |
| Andean Latin America | 0.7111 | 0.3281 | 2.1676 | 0.0310 |
| Caribbean | 0.4409 | 0.2040 | 2.1616 | 0.0315 |
| Central Latin America | 0.4442 | 0.2018 | 2.2012 | 0.0285 |
| Central Sub-Saharan Africa | 0.5018 | 0.2168 | 2.3146 | 0.0214 |
| Eastern Sub-Saharan Africa | 0.5295 | 0.1422 | 3.7238 | 0.0002 |
| North Africa and Middle East | 0.5428 | 0.1731 | 3.1350 | 0.0019 |
| South Asia | 0.9877 | 0.2303 | 4.2889 | <0.0001 |
| Southeast Asia | 1.0880 | 0.1584 | 6.8677 | <0.0001 |
| Western Sub-Saharan Africa | 0.3112 | 0.1117 | 2.7851 | 0.0057 |
| **B. Temporal trends** | **edf** | **Ref.df** | **F-value** | **p-value** |
| Other | 1.0000 | 1.0000 | 2.1620 | 0.1426 |
| Armenia | 1.0000 | 1.0000 | 28.8634 | <0.0001 |
| Central Sub-Saharan Africa | 1.0000 | 1.0000 | 12.2633 | 0.0005 |
| Eastern Sub-Saharan Africa | 1.0000 | 1.0000 | 17.5572 | <0.0001 |
| Latin America and Caribbean | 1.0000 | 1.0000 | 5.2352 | 0.0229 |
| Niger | 1.0000 | 1.0000 | 32.4614 | <0.0001 |
| Nigeria | 2.5821 | 2.5821 | 5.3119 | 0.0141 |
| North Africa and Middle East | 1.6267 | 1.6267 | 5.2548 | 0.0467 |
| South Asia | 1.0000 | 1.0000 | 9.7128 | 0.0020 |
| Zimbabwe | 1.0000 | 1.0000 | 5.3095 | 0.0219 |
| **C. Covariate smooth terms** | **edf** | **Ref.df** | **F-value** | **p-value** |
| ANC1_coverage_prop | 1.0000 | 1.0000 | 9.8151 | 0.0019 |
| he_cap | 1.0000 | 1.0000 | 5.3696 | 0.0212 |

**Table 2. Summary of best public fraction model with pruned region temporal trends and region factors as well as random effects for the units.**

| **A. Regional intercepts** | **Estimate** | **Std. Error** | **t-value** | **p-value** |
| --- | --- | --- | --- | --- |
| Other | 0.2215 | 0.1831 | 1.2100 | 0.2273 |
| Central Asia | 1.4889 | 0.4671 | 3.1875 | 0.0016 |
| Central Sub-Saharan Africa | 0.7454 | 0.3437 | 2.1685 | 0.0310 |
| Eastern Sub-Saharan Africa | 1.1260 | 0.2122 | 5.3059 | <0.0001 |
| Latin America and Caribbean | 0.9135 | 0.2122 | 4.3262 | <0.0001 |
| South Asia | -0.8865 | 0.3645 | -2.4319 | 0.0156 |
| Southern Sub-Saharan Africa | 0.6298 | 0.3730 | 1.6887 | 0.0924 |
| Western Sub-Saharan Africa | 0.8048 | 0.1746 | 4.6100 | <0.0001 |
| **B. Temporal trends** | **edf** | **Ref.df** | **F-value** | **p-value** |
| Other | 1.6009 | 1.6009 | 2.0189 | 0.2855 |
| Eastern Sub-Saharan Africa | 1.0001 | 1.0001 | 8.6479 | 0.0035 |
| Latin America and Caribbean | 1.0103 | 1.0103 | 30.1683 | <0.0001 |
| Niger | 1.0104 | 1.0104 | 3.3276 | 0.0674 |
| North Africa and Middle East | 1.0020 | 1.0020 | 5.6612 | 0.0179 |
| Pakistan | 1.0000 | 1.0000 | 6.8878 | 0.0092 |
| Tanzania | 1.0004 | 1.0004 | 5.1042 | 0.0247 |
| Western Sub-Saharan Africa | 1.4903 | 1.4903 | 5.9552 | 0.0348 |
| **C. Covariate smooth terms** | **edf** | **Ref.df** | **F-value** | **p-value** |
| prop_urban | 1.0017 | 1.0017 | 4.8771 | 0.0280 |
| IFD_coverage_prop | 1.7917 | 1.7917 | 5.3823 | 0.0040 |
| hospital_beds_per1000 | 1.8518 | 1.8518 | 5.9352 | 0.0176 |


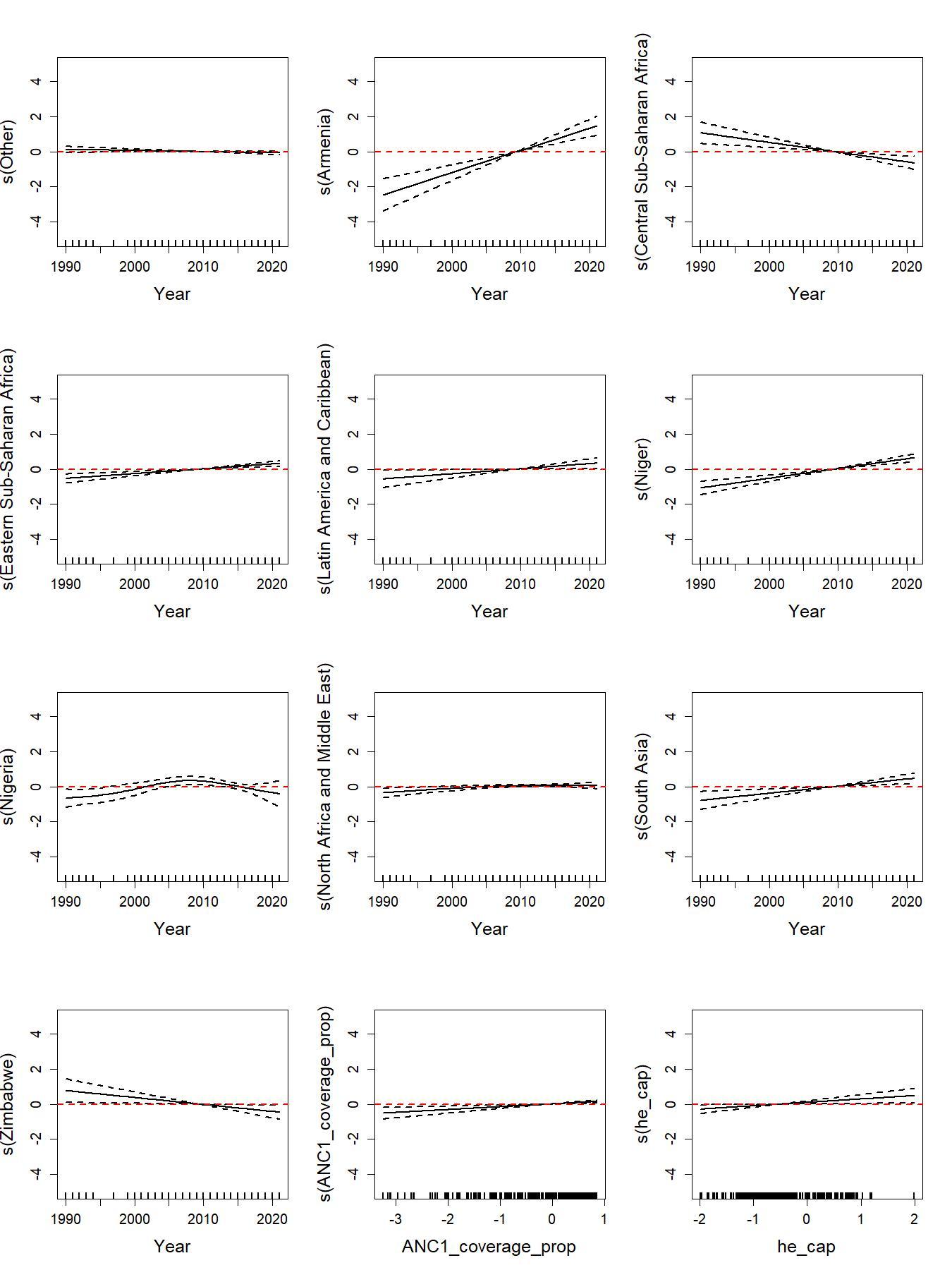


**Fig 1. Any treatment-seeking: Estimated region-specific temporal trends and effects of the covariates.**


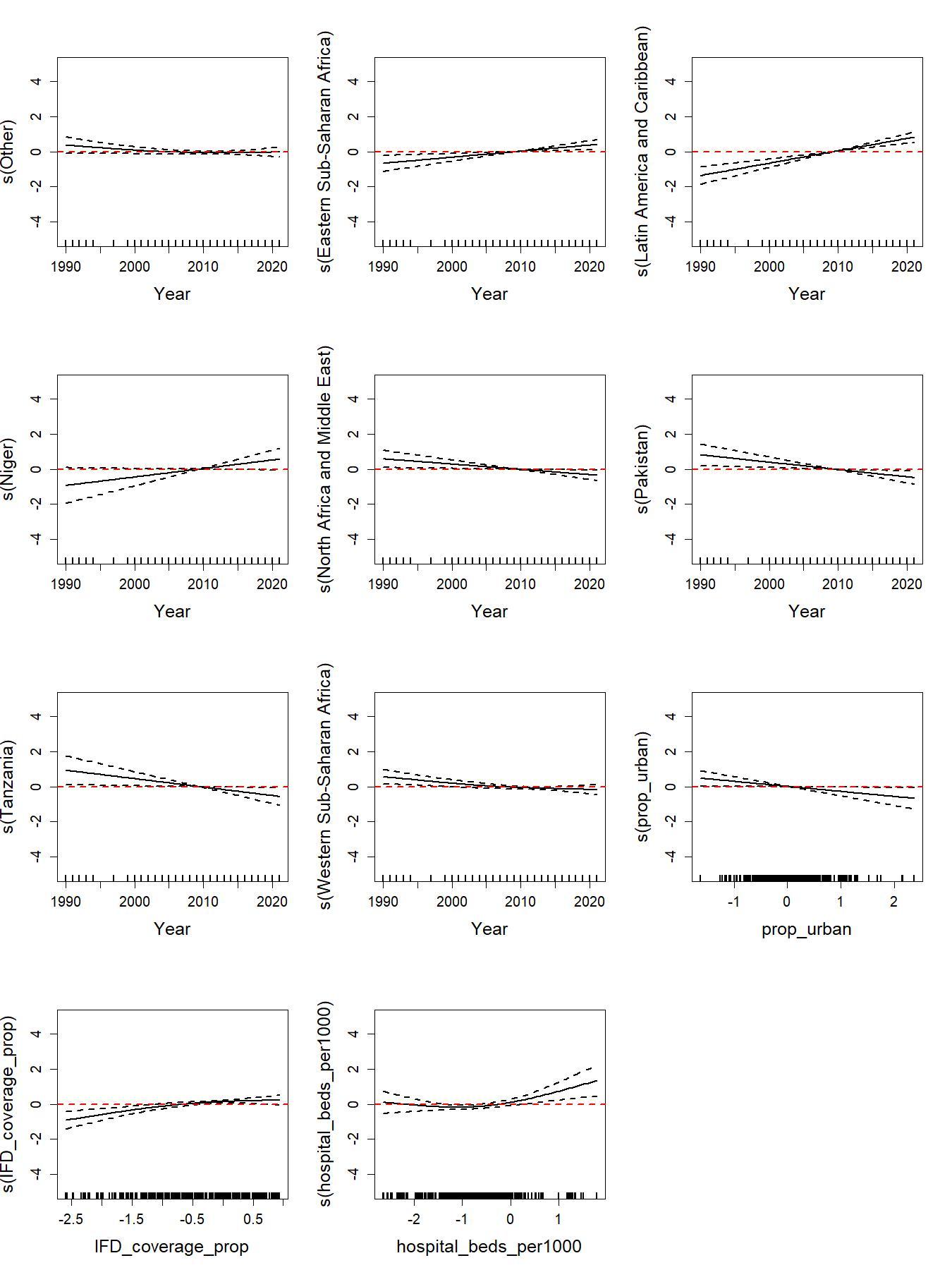


**Fig 2. Public fraction: Estimated region-specific temporal trends and effects of the covariates.**

For any treatment-seeking, there was a positive linear temporal trend in Eastern Sub-Saharan Africa, Latin America and Caribbean, and South Asia. A positive linear trend was also identified in Niger and a few nearby countries (Burkina Faso, Sierra Leone, and Mali), and so these were grouped into a ‘Niger’ region (the region was named after the country with the most data supporting the particular trend). In a similar fashion, we identified a separate ‘Nigeria’ trend, consisting of Nigeria, Benin, Chad and Sao Tome and Principe, which was a non-linear trend, rising until 2008 and slightly declining moving towards 2020. Linear negative trends were estimated for Central Sub-Saharan Africa and for Zimbabwe, for which a separate temporal trend was estimated due to the distinctiveness of its trend relative to other countries in Southern Sub-Saharan Africa. Similarly, we also estimated a separate temporal trend for Armenia, as it was the only country in Central Asia for which the data supported the existence of a strong positive linear trend. A significant positive non-linear temporal trend was also identified for North Africa and the Middle East. The following additional groupings were applied after a close inspection of the dataset: Central Latin America was grouped with the ‘Other’ trend, all countries in South Asia except for Nepal and Bangladesh were grouped with the ‘Other’ trend, and so were Guyana, Jordan, Malawi, Kenya, Madagascar, Uganda, and Gambia – this was done as the observed trend in those countries appeared to be flat. Finally, Lesotho was grouped with Eastern Sub-Saharan Africa due to its closeness and similarity of the observed data trends. In terms of covariates, the model estimated a positive linear relation of the proportion of pregnant women receiving any antenatal care from a skilled provider and health expenditure per capita with any medical treatment-seeking rate for childhood fever.

For public fractions, a positive linear trend was only estimated for Latin America and Caribbean, and Eastern Sub-Saharan Africa. Additionally, a ‘Niger’ region distinct from the one identified for any treatment (i.e., consisting of Niger, Chad, Sao Tome and Principe and Sierra Leone), was also identified to have a positive linear trend. For the remaining regions, negative trends were estimated: North Africa and the Middle East, Western Sub-Saharan Africa, Tanzania (consisting of only Tanzania) and ‘Pakistan’ (consisting of Pakistan, Nepal, and Indonesia). The following additional groupings were applied from the dataset inspection: Andean Latin America was grouped with the ‘Other’ trend, as was Ethiopia, Madagascar, Mozambique, and Rwanda. Zimbabwe was grouped with Eastern Sub-Saharan Africa, and Mali was grouped with North Africa and Middle East. The covariates selected for the final public fraction model were urbanicity (with a negative linear relationship to the logit of public fractions), the proportion of women giving birth at health facilities (with a non-linear positive relationship) and hospital beds per 1000 people (with a positive non-linear relationship above a certain threshold).

Note that the region-specific trends (Fig 1 and Fig 2) in these models may not correspond directly to the trends in the region-averaged estimates since those account for changes in the covariates and populations. From the Normal Q-Q plots in Fig 3 we see that while there are some slight deviations at the tails, the estimated random effects at the unit level have the vast majority of their distributions matching that of the assumed Gaussian distributions.


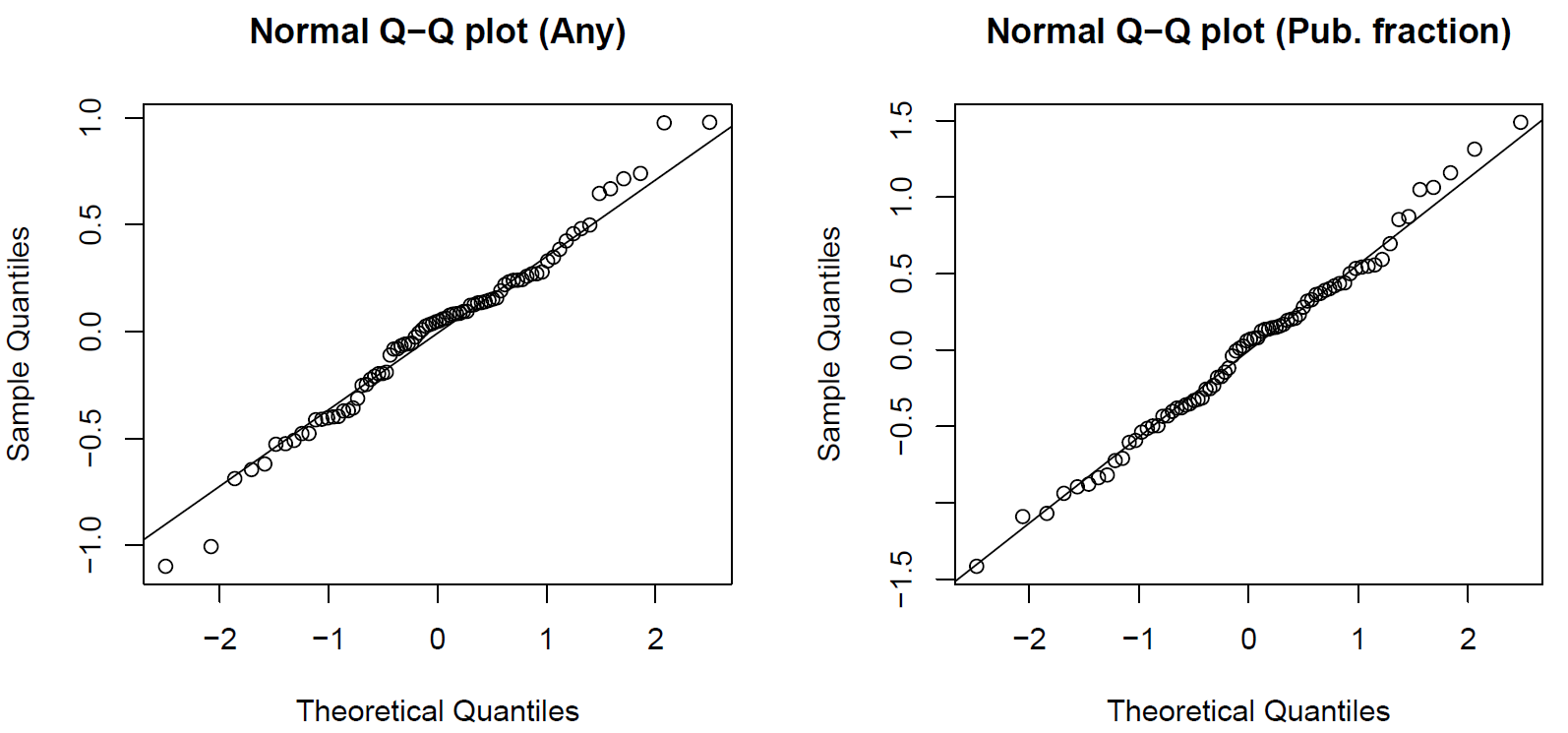


**Fig 3. Normal quantile-quantile plots of the residuals from the any treatment-seeking and the public fraction models.**

# Model evaluation

Plots of the model residuals against the fitted values as well as the histograms of the residuals are shown in Fig 4. Since there is no obvious relation between the residuals and the fitted values and the residuals are relatively symmetrically distributed, the model assumptions do not seem to be violated. The reasonable model fits are also seen through the plots of the fitted against the observed values in Fig 5.


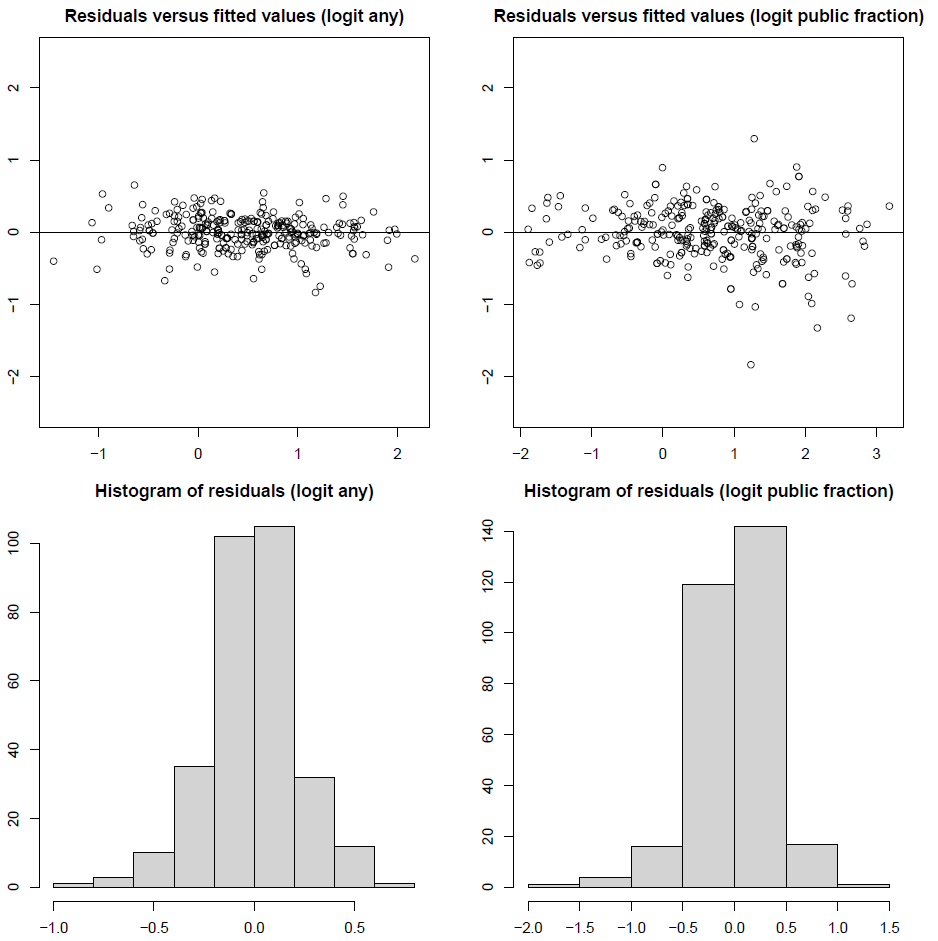


**Fig 4. Plots of the model residuals against the fitted values as well as the histograms of the residuals for the any treatment-seeking and the public fraction models.**


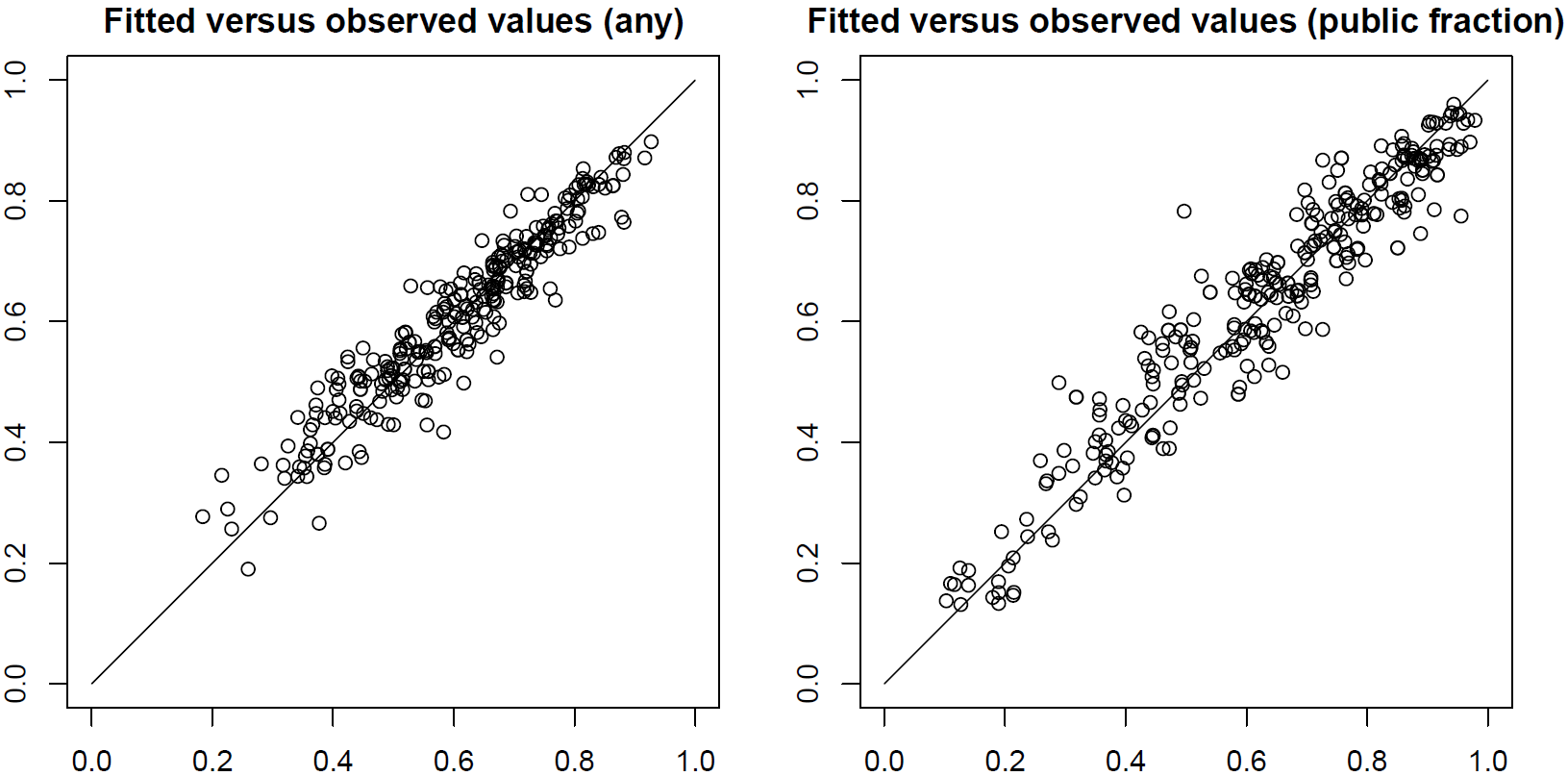


**Fig 5. Plots of the fitted values against the observed values for the any treatment-seeking rates and public fractions.**

For an initial model evaluation, we use the default regional trends and set the random effect to zero if the test unit does not appear in the training data. The any treatment-seeking model had a training error of 4.82% and a test error of 7.88% while the public fraction model had a training error of 6.31% and a test error of 10.94%. The training and test errors are of similar magnitude. This suggests that there is negligible over-fitting, and we can use the model structure for predicting time series of treatment-seeking values.

# Prediction

Since it is difficult to tell if the default regional trend and a random effect of zero is reasonable when we have no data or just one data point for a unit, we use the proximity in covariate space to match such units to their “nearest-neighbour'' (NN) countries within the same super-region and use their regional trends and random effect values.

To select a temporal trend for units with less than two data points, or the data points spanning a period of less than 5 years, we match each unit to a country in the same IHME super-region based on a principal component (PCA) analysis on the differences in the chosen covariates for the any/public treatment-seeking models between the years 2000, 2010 and 2020. Similarly, we select the random effect value for units without any data from the random effects estimated for units with data in the same IHME super region. PCA and NN on the significant covariates in the years 2000, 2010 and 2020 was used. The matched units for regional trends and random effects based are tabulated in Table S5.

To account for covariate and input data variability, 100 samples of the covariates and input data were used to fit the same model configurations 100 separate times. From each fitted model, we simulate 100 realisations for the estimates. The final any treatment-seeking and public fraction estimates and uncertainty intervals were obtained by combining 100*100 = 10,000 estimates for each unit-year pair. Plots of the final estimates for the any treatment-seeking and public treatment-seeking rates are given in Fig S2 and S3.

# References

1. Battle KE, Bisanzio D, Gibson HS, Bhatt S, Cameron E, Weiss DJ, et al. Treatment-seeking rates in malaria endemic countries. Malar J. 2016;15: 20. doi:10.1186/S12936-015-1048-x

2. Earth Observation Group. DMSP nighttime lights. Golden, Colorado 80401, USA; 2021. Available from: https://eogdata.mines.edu/products/dmsp/

3. Earth Observation Group. VIIRS Nighttime Light. [cited 20 Oct 2022]. Available from: https://eogdata.mines.edu/products/vnl/

4. Weiss DJ, Nelson A, Gibson HS, Temperley W, Peedell S, Lieber A, et al. A global map of travel time to cities to assess inequalities in accessibility in 2015. Nature. 2018;553: 333–336. doi:10.1038/nature25181

5. Weiss DJ, Nelson A, Vargas-Ruiz CA, Gligorić K, Bavadekar S, Gabrilovich E, et al. Global maps of travel time to healthcare facilities. Nat Med. 2020;26: 1835–1838. doi:10.1038/s41591-020-1059-1

6. Barton K. MuMIn: multi-model inference. R package version 1.40.4. In: 2018 [Internet]. [cited 20 Oct 2022]. Available from: http://mumin.r-forge.r-project.org/
